# From Carbon Nitrides to COFs: Opportunities and Prospects in Photocatalytic CO_2_ Reduction

**DOI:** 10.1002/adma.202506961

**Published:** 2025-08-29

**Authors:** Wei Che, Songlin Zhao, Woo Jin Byun, Tao Tao, Jong‐Pil Jeon, Qiannan Zhao, Yanhua Shao, Jian Li, Jeongwon Kim, Jae Sung Lee, Jong‐Beom Baek

**Affiliations:** ^1^ School of Materials and Energy Guangdong University of Technology Guangzhou 510006 P. R. China; ^2^ School of Energy and Chemical Engineering Ulsan National Institute of Science and Technology (UNIST) Ulsan 44919 Republic of Korea; ^3^ Emergent Hydrogen Technology R&D Centre Ulsan National Institute of Science and Technology (UNIST) Ulsan 44919 Republic of Korea; ^4^ Graduate School of Carbon Neutrality Ulsan National Institute of Science and Technology (UNIST) Ulsan 44919 Republic of Korea

**Keywords:** functional linkage, light‐responsive materials, photocatalytic CO_2_ conversion, polymeric photocatalysts, structural regulation

## Abstract

The continuing increase in atmospheric carbon dioxide (CO_2_), a major greenhouse gas and accelerating climate change are driving demand for innovative mitigation strategies. The photocatalytic CO_2_ reduction reaction (PCO_2_RR) presents a promising and sustainable route to convert CO_2_ into useful hydrocarbons and fuels utilizing sunlight, thereby mitigating CO_2_ emissions. This review examines the developmental aspects of light‐driven CO_2_ conversion using organic polymeric photocatalysts, focusing on carbon nitrides (CNs), covalent triazine frameworks (CTFs), and covalent organic frameworks (COFs). These materials are verified to possess great potential for PCO_2_RR, because they offer tunable band gaps, large surface areas, efficient light absorption, and remarkable activity and selectivity in photocatalysis. In this review, the comprehensive analysis of photocatalytic materials (e.g., CNs, CTFs, and COFs) are thoroughly discussed, along with their mechanisms, historical advancements, and urgent roles in PCO_2_RR. Strategies for enhancing the CO_2_ photoreduction efficiency of CNs, CTFs, and COFs are also highlighted. Each organic material brings distinct advantages. The review also addresses critical challenges, such as improving efficiency and managing charge transport dynamics for PCO_2_RR. Finally, the review underscores the need to develop scalable CO_2_ conversion applications, advance organic photocatalyst material science, and support sustainable energy conversion technologies.

## Graphical abstract

To underscore the potential of polymeric photocatalysts for CO_2_ photoreduction—from carbon nitrides to covalent organic frameworks—the table of contents graphic presents recent breakthroughs in the design of light‐responsive organic photocatalysts. These include strategies such as junction engineering, non‐metallic site tuning, enhancement of crystallinity, metal incorporation, structural modulation, and optimization of molecular linkages.

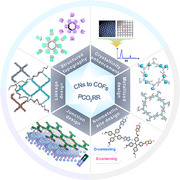

## 1 Introduction

Surging levels of atmospheric CO_2_ from industrial activities and fossil fuel combustion are driving climate changes, including global warming and ocean acidification. Addressing these challenges requires the development of innovative technologies, especially those that can reduce CO_2_ emissions and provide sustainable alternative sources of energy. Among the various strategies, the photocatalytic CO_2_ reduction reaction (PCO_2_RR) stands out as a highly promising approach for transforming CO_2_ into valuable chemicals using solar energy, a renewable and abundant resource.^[^ ^1^ ^]^ The light‐driven catalysis on semiconductors was first brought to prominence by Fujishima and Honda in the early 1970s with their discovery of water hydrolyzing using a TiO_2_ electrode under the UV spectrum. This breakthrough laid the groundwork for various photocatalytic systems, including those aimed at CO_2_ transformation processes.^[^ ^2^ ^]^ From then on, extensive work has focused on developing new photocatalysts to improve their effectiveness as solar energy harvesters across a broader spectrum range, with a focus on visible light.^[^ ^3^ ^]^ It is worth noting that polymeric materials, such as crystalline organic semiconductors, have emerged as extraordinary photocatalysts for PCO_2_RR in recent years. Their unique properties, including tunable electronic structures, efficient absorption of visible light, and high stability, render them ideal platforms for the production of solar fuels from CO_2_ photoreduction.^[^ ^4^ ^]^ For example, melon‐based “graphitic carbon nitride” (g‐C_3_N_4_, g‐CN), synthesized from nitrogen (N)‐rich organic precursors with graphitic sp^2^‐hybridized C and N atoms, is a widely studied polymeric photocatalyst with excellent CO_2_ reduction photoactivity. Its structure allows for various modifications, including doping and defect engineering, which further enhance its performance.^[^ ^5^ ^]^

Recent research on the development of organic photocatalysts has attracted significant interest because they offer distinct advantages, including tunable electronic properties, structural robustness, and environmental compatibility. These features, particularly found in polymeric materials from carbon nitrides (CNs), covalent triazine frameworks (CTFs), and covalent organic frameworks (COFs), facilitate electron transfer processes and generate reactive species under light‐driven CO_2_ reduction. Moreover, their flexible frameworks allow for the engineering of targeted functional sites capable of CO_2_ capture under light to produce carbonaceous compounds. In photocatalysis, the interaction between CO_2_ molecules and organic substrates is complex, which involves the generation of charge carriers, CO_2_ adsorption and activation, subsequent photo‐induced electron transfer, and final product formation. Gaining insight into the reaction pathways that happen on the organic substrates is crucial to optimizing photocatalytic performance. Early research focused on pristine organic materials, but over time, more sophisticated designs have been developed, such as heterojunctions, element doping, and metal/polymer composites, which have enhanced the efficiency and selectivity of the reaction.^[^ ^6^ ^]^ Despite these advances achieved in polymeric photocatalysts for CO_2_ chemical fixation, several challenges still need to be researched. One of the key issues is the limited light absorption ability of these materials, which restricts their overall efficiency. Furthermore, ensuring that solar energy is stored in certain desired products, such as hydrocarbons, is a significant hurdle. Also, the stability of the photocatalyst under prolonged irradiation and in the presence of reactive intermediates is another key challenge that needs to be addressed to enable organic photocatalysts viable for real‐world applications.

In this review, the fundamental mechanisms involved in the PCO_2_RR process are presented, along with a historical perspective and the urgent need for advanced CO_2_ fixation technologies. Strategies for enhancing the PCO_2_RR efficiency of CNs, CTFs, and COFs have been highlighted. These strategies focus on various designs, including junction engineering, non‐metallic site design, crystallinity enhancement, different metallization methods (such as metal nanoparticles, complexes, and single atoms) interacting with organic frameworks, structured topography control, and functional linkage design. Despite the progress made in this field, critical issues to scaling up PCO_2_RR technologies remain that need to be addressed. There are five categories to be considered. 1) Improving light absorption, charge transfer efficiency, and product selectivity are ongoing challenges. 2) Ensuring that the carbon detected as a product originates from CO_2_ rather than contaminants in the system is critical for reliable results. 3) Developing photocatalysts that operate efficiently without sacrificial agents is vital for large‐scale applications, yet most current systems still rely on sacrificial agents, which are not practical for such use. 4) Promoting scalable applications is key to making solar‐driven CO_2_ conversion feasible for real‐world implementation. 5) Data‐driven insights, a current research hotspot, are shaping the design of polymeric photocatalysts in accelerating solar‐driven CO_2_ fixation. Finally, we hope that this review highlights the importance of the developments aimed at advancing organic photocatalyst science toward CO_2_ photoconversion and will enable sustainable carbon fix technologies to flourish.

## 2 Fundamental and Developmental Aspects of PCO_2_RR

### 2.1 Fundamental Aspects for PCO_2_RR

#### 2.1.1 Fundamental Mechanism for PCO_2_RR

**Figure 1:**
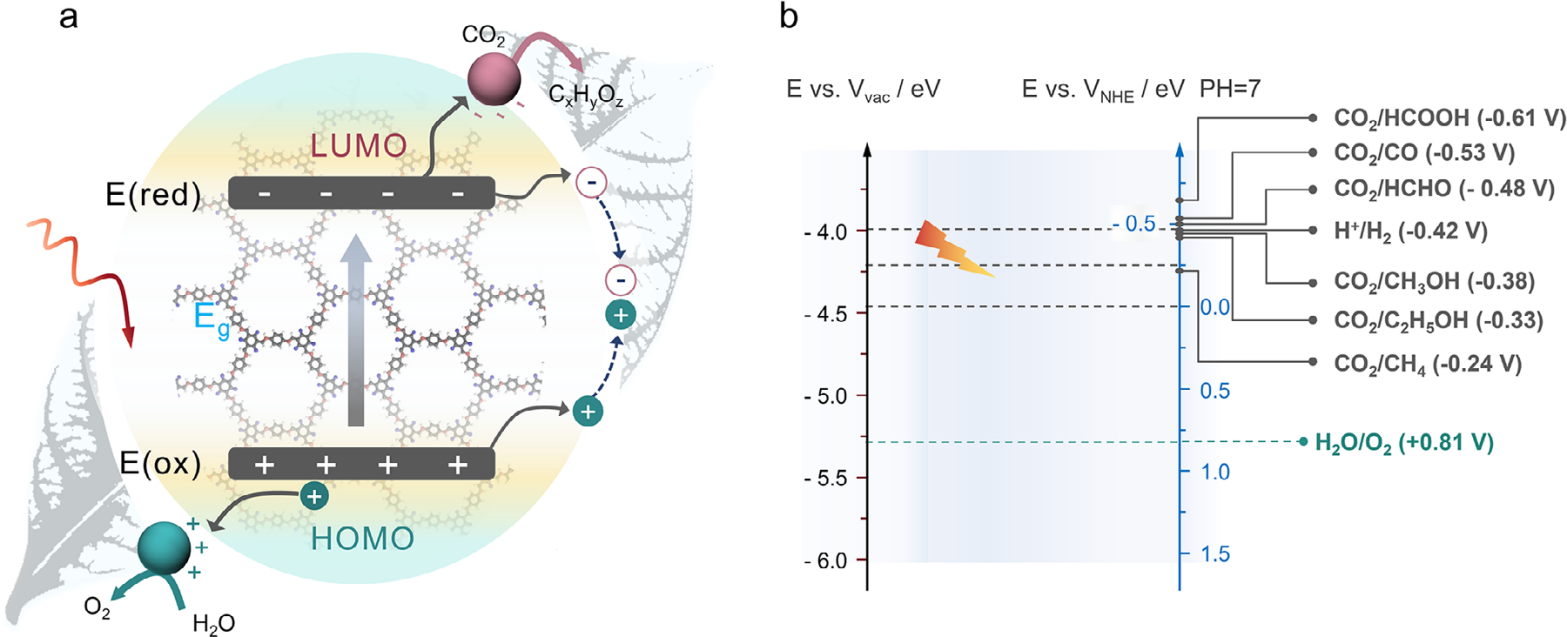
a) Photocatalytic CO_2_ reduction system with semiconductor catalysts. b) Diagram of a typical photocatalytic CO_2_ reduction process, illustrating the relationship between redox potentials and the corresponding products.

Photocatalytic CO_2_ conversion involves a series of steps that need careful management to ensure efficiency. Initially, photons are absorbed by the semiconductor photocatalyst, leading to the excitation of electrons from the valence band (VB) to the conduction band (CB). This process generates electron–hole pairs, which play a crucial role in driving the reductive electron transfer during CO_2_ conversion. As shown in **Figure** **1**a, these charge carriers must be effectively separated and moved to the photocatalyst surface to drive CO_2_ reduction and oxidize a sacrificial agent (typically water).^[^ ^7^ ^]^ Complex processes that occur at the interface of photocatalysts under light excitation result in the transformation of CO_2_ into useful chemicals like carbon monoxide (CO), methane (CH_4_), methanol (CH_3_OH), formic acid (HCOOH), and ethanol (C_2_H_5_OH) etc. However, matching the redox potentials for the potential CO_2_ reduction reaction (CO_2_RR) and ensuring that the light absorber delivers sufficient energy to drive the process remain significant challenges—particularly when integrating the half‐reactions of the CO_2_RR and water oxidation reaction (WOR) into a coupled overall process. Figure 1b illustrates the intricate relationship between the redox potentials of photogenerated carriers and potential products within the integrated CO_2_RR and WOR photo redox systems. The overall efficiency largely depends on the photocatalyst's ability to absorb a broad spectrum of light wavelengths, efficiently separate/transport charge carriers, and provide reactive sites for the process of CO_2_ reduction. Semiconductor materials with a suitable band gap, such as modified TiO_2_, carbon nitrides, and other polymeric semiconductors, are of significant interest for efficient PCO_2_RR.^[^ ^8^ ^]^

#### 2.1.2 Factors Influencing PCO_2_RR Efficiency

Several factors are critical for converting solar photon energy into carbonaceous chemicals during PCO_2_RR. One key factor is the photocatalyst bandgap, which determines light absorption capacity. Another important factor is minimizing the charge carrier recombination rate to maximize the availability of electrons and holes for redox reactions. Additionally, regulating CO_2_ adsorption and activation on the organic surfaces of polymeric materials is necessary to enhance photoreduction efficiency. Besides, the reaction environment, including pH, temperature, and co‐catalysts, also significantly impacts the reaction pathways to generate the target product.^[^ ^9^ ^]^ Herein, we summarize five main factors that affect the light‐driven CO_2_ reduction process. 1) Photon absorption and charge excitation: The process begins with the act of photon absorption by a semiconductor. Upon absorbing light with an energy greater than the bandgap (E_g_), the photoexcited electrons in the VB position jump to the CB level, resulting in the generation of separated electrons and holes. The efficiency of this initial step depends on the E_g_ of the photocatalysts and their light‐harvesting ability over a broad spectrum. 2) Charge migration: Once carriers are generated, effective spatial charge separation is essential to minimize their recombination and the resulting heat loss. The separated electrons and holes migrate to distinct sites on the polymer surface, where redox reactions take place. Surface modifications, such as co‐catalyst deposition, can greatly enhance charge mobility, thereby improving overall photocatalytic performance. 3) CO_2_ adsorption and activation: CO_2_ activation generally involves bending or distorting the normally linear CO_2_ molecule, making it more prone to reduction. The catalyst's surface characteristics, such as morphology and reactive sites, are crucial in determining how effectively CO_2_ is adsorbed and activated. 4) Reduction reaction pathways: Typically, the reaction occurs through multiple proton‐coupled electron transfer (PCET) steps, where electrons reduce CO_2_, while holes oxidize H_2_O/sacrificial donor.^[^ ^10^ ^]^ Different products can be generated during PCO_2_RR, each with its specific half‐reaction and redox potential. Thus, PCO_2_RR may proceed through multiple pathways simultaneously, yielding various products like CH_4_/CO or CH_3_OH/HCOOH.^[^ ^11^ ^]^ 5) To clarify the key parameters and underlying catalytic mechanisms, it is essential to first identify the reactive sites where chemical transformations take place.

#### 2.1.3 Economic Viability of PCO_2_RR

The light‐driven carbon dioxide reduction reaction process emulates the natural photosynthetic mechanism, whereby solar energy is harnessed to transform carbon dioxide and water molecules into valuable and complex chemical compounds. **Table** **1** lists the major products of the PCO_2_RR, together with their corresponding redox potentials, annual production volumes, market prices, and primary applications.^[^ ^12^, ^13^, ^14^ ^]^ The economic viability of this technology mainly hinges on the efficiency of the photocatalytic systems and the market value of the resulting products. Prices for these products vary based on market demand, purity, and regional factors. In 2023, the price of carbon monoxide was relatively low because of its simple composition and abundant supply. The price of methane fluctuates with global natural gas prices, averaging ≈$3 to $6 per million BTUs. Methanol, a widely traded commodity, averages ≈$400 to $600 per ton, while formic acid, often used in specialty applications, can range from $500 to $1,200 per ton depending on purity and usage. In this regard, PCO_2_RR holds the promise of mitigating CO_2_ emissions while supplying critical resources for the chemical and energy sectors.^[^ ^13^ ^]^ This dual capability of reducing CO_2_ emissions while generating renewable fuels makes it advantageous for both addressing environmental issues and fulfilling energy demands.

**Table 1:** The value of major products from photocatalytic CO_2_ reduction.

Product	Half‐reaction of CO_2_RR	Redox potential [V]	Annual production [10^9^ kg]	Market price [US$ per kg]	Applications
CO	CO_2_ + 2e^−^ + 2H^+^ → CO + H_2_O	−0.52	32	0.1	Fischer‐Tropsch synthesis, alkene carbonylation, metallurgy
HCOO^−^	CO_2_ + 2e^−^ + H^+^ → HCOO^−^	−0.41	0.7	0.9	Preservative, antibacterial agent in livestock feed, leather, and textile industry
HCHO	CO_2_ + 4e^−^ + 4H^+^ → HCHO + H_2_O	−0.48	23	0.4	Resin production, polyfunctional alcohols, disinfection
CH_3_OH	CO_2_ + 6e^−^ + 6H^+^ → CH_3_OH + H_2_O	−0.38	85	0.6	Fuel additive, energy source, production of formaldehyde, acetic acid, and methyl tert‐butyl ether
CH_4_	CO_2_ + 8e^−^ + 8H^+^ → CH_4_ + 2H_2_O	−0.24	2,650	0.4	Fuel, syngas production
CH_3_COO^−^	2CO_2_ + 8e^−^ + 7H^+^ → CH_3_COO^−^ + 2H_2_O	−0.29	12	1.4	Solvent, food industry, production of vinyl acetate, acetic anhydride, and esters
CH_3_CHO	2CO_2_ + 10e^−^ + 10H^+^ → CH_3_CHO + 3H_2_O	−0.36	1.0	2.0	Production of 2‐ethyl‐1‐octanol, pentaerythritol
C_2_H_5_OH	2CO_2_ + 12e^−^ + 12H^+^ → C_2_H_5_OH + 3H_2_O	−0.33	81	1.1	Fuel, solvent, beverages, disinfectants
C_2_H_4_	2CO_2_ + 12e^−^ + 12H^+^ → C_2_H_4_ + 4H_2_O	−0.35	214	1.2	Production of ethylene oxide, ethylene dichloride, ethylbenzene, polyethylene
C_2_H_6_	2CO_2_ + 14e^−^ + 14H^+^ → C_2_H_6_ + 4H_2_O	−0.27	134	0.2	Ethylene production, energy generation, cryogenic refrigeration systems

### 2.2 Historical Perspective with a Focus on Polymer‐Based Photocatalysts

#### 2.2.1 Comparative Analysis of Polymeric Materials

**Figure 2:**
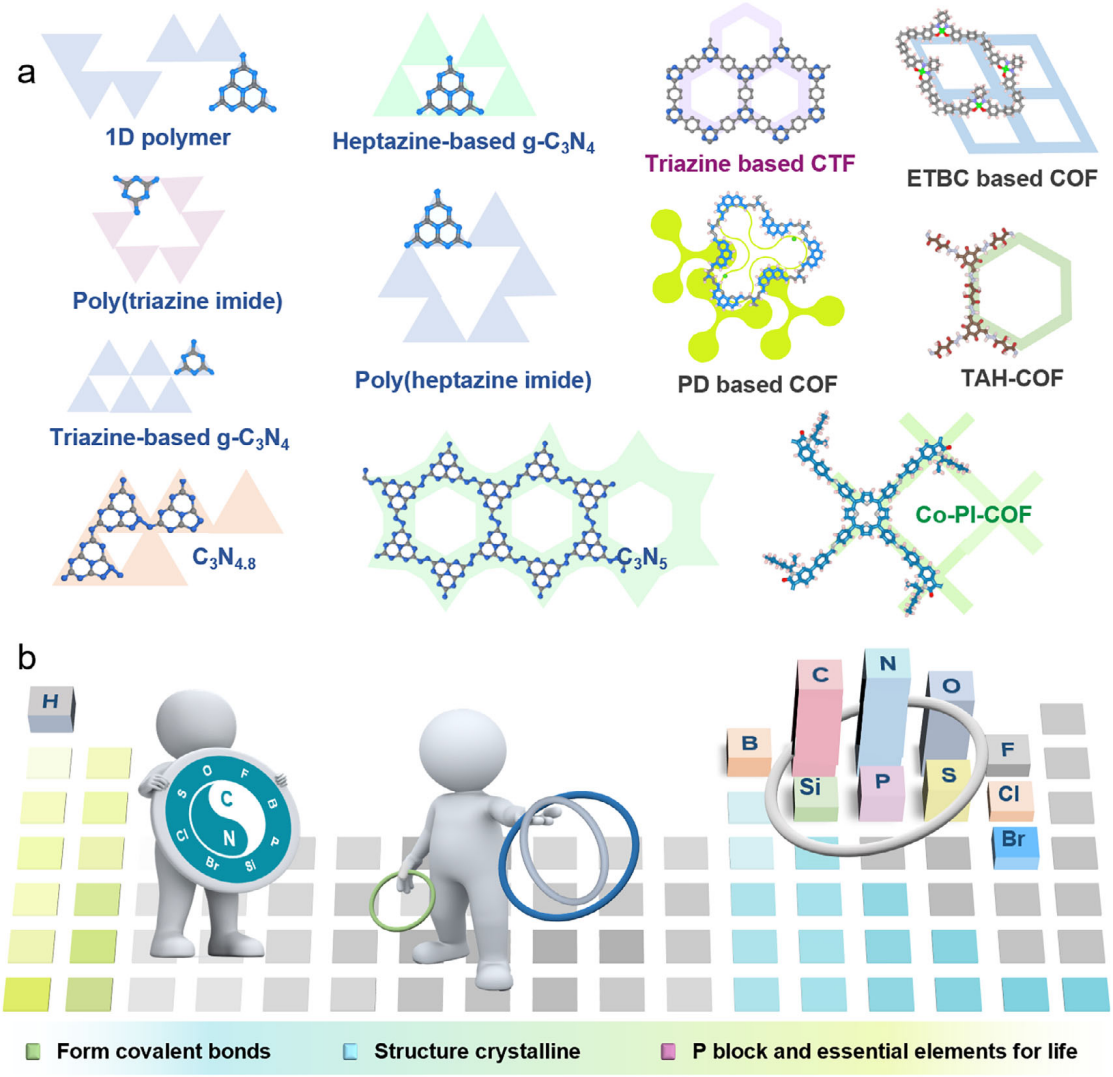
a) Structural characteristics and diversity among carbon‐nitrogen‐based covalent organic materials, including CNs, CTFs, and COFs. b) The commonalities among CNs, CTFs, and COFs: ordered periodic local structures formed via covalent bonds, good crystallinity, and chemical compositions rich in p‐block and essential elements for life.

As illustrated in **Figure** **2**a, CNs possess the simplest structure, mainly existing in layered forms with relatively limited variations. CTFs possess a certain level of structural diversity, though their framework is largely constrained by triazine‐rich units. Their strong π–π conjugation is advantageous for enhancing light absorption. Compared to CNs and CTFs, COFs exhibit the greatest structural diversity, allowing the construction of various topologies through the combination of diverse monomers and linkages. Moreover, COFs can be precisely tailored with π‐conjugated structures and various functional groups to tune their bandgaps for visible‐light utilization. These polymeric frameworks, while sharing common features in elemental composition and covalent bonding, also show distinct differences, offering great potential for PCO_2_RR.

Specifically, CNs are characterized by their local structural order, typically composed of s‐triazine (C_3_N_3_) and tri‐s‐triazine (C_6_N_8_) motifs (commonly referred to as g‐C_3_N_4_). Notably, g‐C_3_N_4_ is mainly built from heptazine (tri‐s‐triazine) units. Recently, other 2D CNs such as poly(triazine imides) (PTI) and poly(heptazine imides) (PHI) semiconductors have work as promising candidates for photocatalytic CO_2_ capture. Besides, based on the structural variations, there are additional four 3D carbon nitrides—α‐C_3_N_4_, β‐C_3_N_4_, cubic C_3_N_4_, and pseudo‐cubic C_3_N_4_—have been theoretically proposed based on structural variations.^[^ ^15^, ^16^ ^]^ Most recently, in addition to the crystalline PTI and PHI frameworks, other new types of crystalline carbon nitrides, such as C_3_N_4.8_ and C_3_N_5_, which typically feature periodic heptazine or heptazine‐like units, have been developed and shown excellent potential for PCO_2_RR.^[^ ^17^, ^18^, ^19^ ^]^ For example, C_3_N_5_, a nitrogen‐rich carbon nitride material formed by two s‐heptazine units bridged with an azo linkage, have been reported and served as an effective photocatalyst for PCO_2_RR.^[^ ^20^, ^21^ ^]^ To add to that, both theoretical and experimental studies have shown that heteroatom doping of heptazine (C_6_N_8_) motifs (e.g., replacing partial C or N atoms) can induce charge localization, thereby enhancing PCO_2_RR performance for the highly selective CH_4_ generation.^[^ ^22^, ^23^ ^]^ For example, Lin's group reported that doping heptazine (C_6_N_8_) structures with p block elements such as B, P, O, or S results in substitution at the pyridinic nitrogen site. The obtained two catalysts composed of C_6_N_7_S_1_ and C_6_N_7_O_1_ motifs facilitate CO_2_ activation and the hydrogenation of COOH* into CO* to produce the CO product.^[^ ^24^ ^]^ Previous studies show that triazine units are photoactive centers and have been widely designed within polymeric materials to mimic catalytic centers like those in chloroplasts.^[^ ^25^ ^]^ Therefore, CTFs, which possess ordered structures with high nitrogen content and are mainly composed of triazine cores (C_3_N_3_), have thus been employed to imitate these catalytic centers, thereby enhancing the photocatalytic reduction of CO_2_.^[^ ^26^ ^]^ Also, their properties can be further tuned via heteroatom functionalization.^[^ ^27^ ^]^ Compared with CNs and CTFs, COFs not only incorporate triazine units but also allow greater structural flexibility. They include diverse elements (B, C, N, O, F, Si, P, S, Br) and covalent bonds (C–S, P–C, B–O, B–N, etc.), offering design versatility for optimizing light absorption, charge transfer, and CO_2_ affinity.^[^ ^28^, ^29^, ^30^ ^]^

As shown in Figure 2a and **Table** **2**, from CNs and CTFs to COFs, these frameworks share notable structural similarities. CNs generally have low to moderate crystallinity, while CTFs and COFs exhibit moderate to high crystallinity. All of these organic semicontors are covalent frameworks with ordered periodic structures. Beyond structural similarities, recent advances in backbone design and elemental diversification have yielded frameworks rich in p‐block elements. Incorporating B, O, N, S, and P enhances optoelectronic properties and catalytic activity, thus expanding their potential in PCO_2_RR (Figure 2b). Therefore, this review highlights carbon‐nitrogen‐based covalent organic materials, including CNs, CTFs, and COFs, offering a comprehensive discussion on their development, synthesis, structure, and photocatalytic CO_2_ reduction applications.

**Table 2:** Comparison of characteristics and photocatalytic CO_2_ reduction applications of the polymeric materials among CNs, CTFs, and COFs.

Materials	Crystallinity	Framework type	Band tunability	Synthesis	PCO_2_RR application
CTFs	Moderate to high crystalline	Triazine‐centered linkages	Potentially tunable for PCO_2_RR, but reliance on monomer structure	Trimerization of nitriles Cyanogen‐based polymerization Imine/aldehyde‐amine coupling	Utilized
COFs	Moderate to high crystalline	Functionally versatile monomers‐based motifs	Tunable for CO_2_RR redox, but limited for overall CO_2_RR‐WOR requirements	Boronate ester formation Hydrazone formation Schiff base condensation Olefin metathesis	Widely utilized
CNs	Low to moderate, high crystalline	1) Triazine‐based type Triazine‐based g‐C_3_N_4_ Triazine‐based PTI 2) Heptazine‐based type Heptazine‐based g‐C_3_N_4_ Heptazine‐based PHI	CB position is applicable to CO_2_RR and VB position for WOR can be tuned via heteroatom doping or copolymerization	1) Trimerization from triazine‐ or heptazine‐based monomer 2) Polymerization of C, N, S‐containing precursors: thiourea, urea, dicyandiamide	Widey utilized

#### 2.2.2 History of Polymeric Materials as Photocatalysts for CO_2_ Reduction

**Figure 3:**
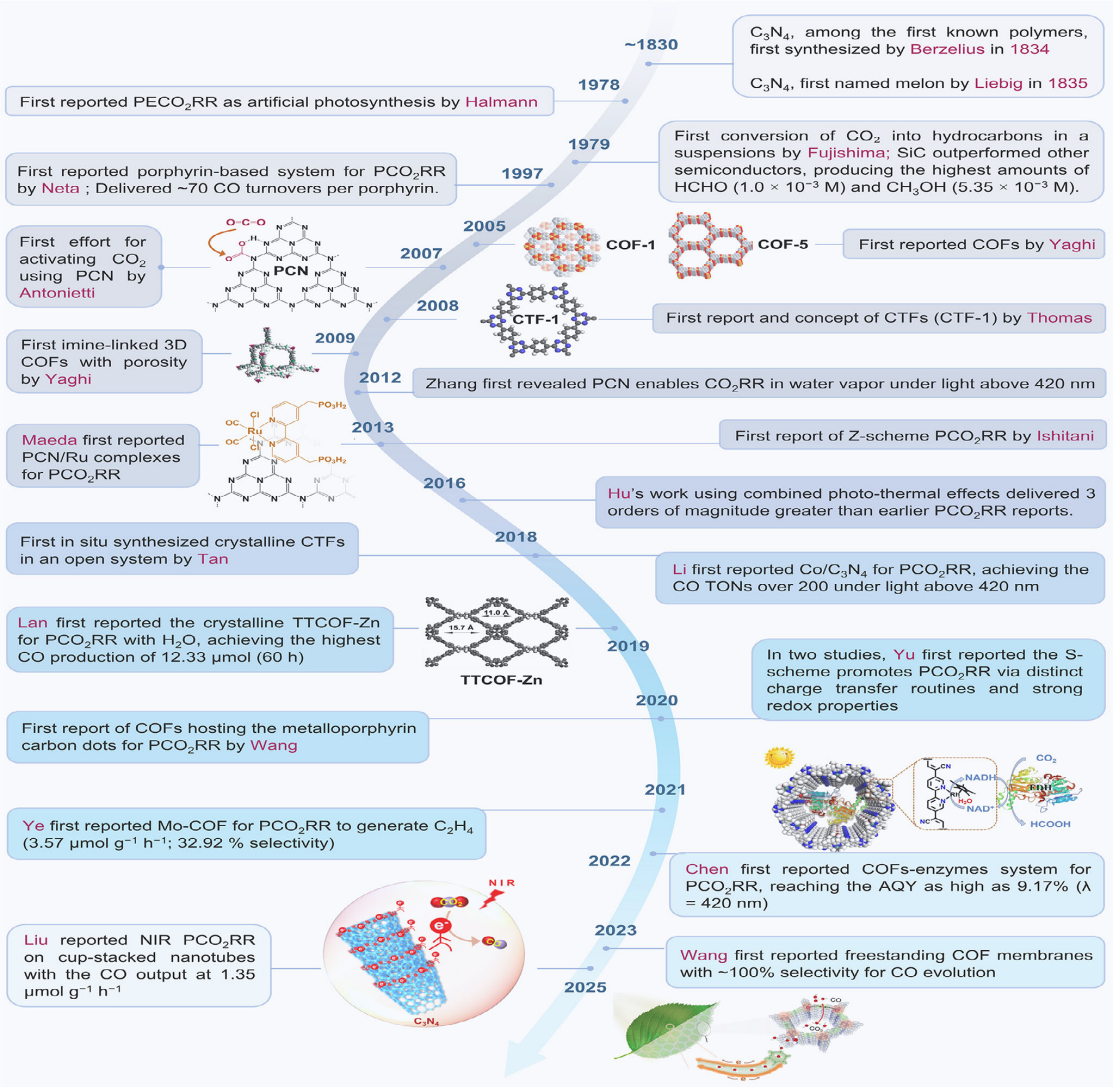
Historical trajectory of PCO_2_RR research, annotated with representative performance benchmarks achieved by different porous frameworks platforms, from CN to CTFs and COFs.

The evolution of photocatalytic CO_2_RR technologies has spanned nearly two centuries, beginning with the discovery of g‐C_3_N_4_ and continuing with breakthroughs in the design and functionality of polymeric photocatalysts, including CTFs and COFs. This timeline highlights the persistent efforts to refine materials capable of efficiently converting CO_2_ using solar energy in pursuit of sustainable, carbon‐neutral solutions. As illustrated in **Figure** **3**, the past records of polymeric photocatalysts begins in 1834, when Berzelius first successfully synthesized polymeric carbon nitride (PCN), which was later given the name “melon” by Liebig in 1835.^[^ ^31^, ^32^ ^]^ These early milestones set the stage for developing N‐rich materials, which were eventually explored for catalysis. In 1978, Halmann was the first to successfully demonstrate the photoelectrochemical reduction of CO_2_, introducing the groundbreaking concept of “artificial photosynthesis”, which has since inspired extensive research in renewable energy conversion.^[^ ^33^ ^]^ The milestone was soon followed by Fujishima's 1979 report on CO_2_ photoreduction using semiconductors in aqueous suspension, establishing the viability of semiconductor‐based photocatalysts.^[^ ^34^ ^]^ The early 21st century saw a significant leap with the application of PCN for PCO_2_RR, with Antonietti's team making the first attempt to activate CO_2_ using PCN materials in 2007.^[^ ^35^ ^]^ This was followed by Zhang's report in 2012, showcasing the successful use of PCN for PCO_2_RR.^[^ ^36^ ^]^

Parallel to the progress with PCN, the introduction of CTFs and COFs opened a novel category of porous, crystalline materials. In 2005, Yaghi reported the first COFs, laying the groundwork for integrating light‐responsive and catalytic functions within a tunable framework.^[^ ^37^ ^]^ Shortly after, in 2008, the concept of CTFs was first introduced by Thomas, which further enhanced the structural and chemical stability of photocatalytic materials due to their N‐rich triazine units.^[^ ^38^ ^]^ In 2009, Yaghi reported the first example of crystalline imine‐linked 3D COFs with permanent porosity, pioneering advancements in porous materials.^[^ ^39^ ^]^ Both CTFs and COFs began to attract attention for their potential to improve CO_2_ reduction across a broad spectrum. Later, the field advanced with the development of frameworks with catalytic centers by integrating metal complexes.^[^ ^40^, ^41^ ^]^ Another critical advance came in 2013 when Ishitani introduced the Z‐scheme concept for PCO_2_RR, which improved the separation of charge carriers for enhancing overall conversion efficiency.^[^ ^42^ ^]^ Then, many works demonstrated the remarkable potential of these hybrid materials (COFs/Re complex) in boosting CO_2_ photoreduction efficiency.^[^ ^43^, ^44^, ^45^ ^]^ Further, Hu demonstrated the first photocatalytic CO_2_ reduction to CH_4_ utilizing synergistic photothermal effects in 2016, marking a significant advance in combining light and heat for efficient CO_2_ conversion.^[^ ^46^ ^]^ Lan et al. were the first to utilize crystalline TTCOFs‐Zn in 2019 for PCO_2_RR using pure H_2_O as the electron donor. This system delivered a notable CO output of 12.33 µmol within 60 h, representing a pivotal development toward CO2 photoreduction by using COFs photocatalysts.^[^ ^47^ ^]^ Recently, by Yu and Fan in 2020, the first introduction of S‐scheme heterojunctions for PCO_2_RR has emerged and work efficiently for improving charge separation and light absorption.^[^ ^48^, ^49^ ^]^

More recently, innovative approaches—such as enhancing structural crystallinity, hybridizing with carbon dots, metals, or enzymes, and achieving efficient PCO_2_RR in pure water—have greatly expanded the functional scope and practical potential of these organic semiconductors.^[^ ^50^, ^51^, ^52^, ^53^, ^54^, ^55^ ^]^ For example, an advanced hybrid plasmonic system by coupling cup‐stacked carbon nanotubes with graphitic carbon nitride (CNN), exhibited superior near‐infrared PCO_2_RR. Under near‐infrared irradiation, CNN achieves nearly complete CO_2_ reduction to CO at 1.35 µmol g^−1^ h^−1^. The unique edge‐plane structure significantly boosts local plasmonic fields and sustains interfacial electronic states. The performance improvement stems from fully accessible edge sites enabling targeted hot‐electron transfer and efficient charge extraction, representing a breakthrough in metal‐free plasmonic NIR photocatalysts.^[^ ^56^ ^]^ With continuous innovation, polymeric frameworks are becoming key enablers of efficient and sustainable CO_2_ reduction. Mapping out their historical trajectory and breakthrough milestones offers valuable insight into how these materials have evolved—demonstrating not only the technological advancements but also the increased structural and functional complexities.

### 2.3 Urgent Need for Polymer‐Based Photocatalysts for PCO_2_RR

**Figure 4:**
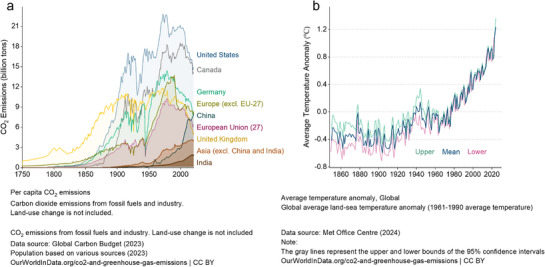
a) Annual CO_2_ emissions by world region. “CO_2_ emissions dataset: our sources and methods” published online at OurWorldinData.org. b) Global average anomaly in land‐sea temperatures, measured with the long‐term average temperature. Data adapted from the National Ocean and Atmospheric Administration (NOAA) website. Copyright 2024, NOAA.

Human activities, particularly fossil fuel consumption, have significantly boosted CO_2_ emissions, driving global warming, ocean acidification, and more frequent extreme weather events. As shown in **Figure** **4**a, CO_2_ emissions have risen steadily across regions, with the US, China, and Europe being the largest historical contributors. Despite global emission reduction efforts, the overall trend remains upward, indicating the need for more effective mitigation strategies. Figure 4b illustrates the direct link between rising CO_2_ levels and global temperature increases. Without significant action, these trends will lead to severe impacts on ecosystems, weather, and society. Achieving the ambitious climate goals of agreements like the Paris Agreement requires both reducing CO_2_ emissions and developing CO_2_‐to‐fuels technologies.^[^ ^57^ ^]^ Therefore, reducing greenhouse gases while generating renewable energy aligns with carbon neutrality goals. Given steadily rising emissions and the slow rate of clean energy adoption, developing efficient, scalable, and cost‐effective PCO_2_RR systems is urgent.

**Figure 5:**
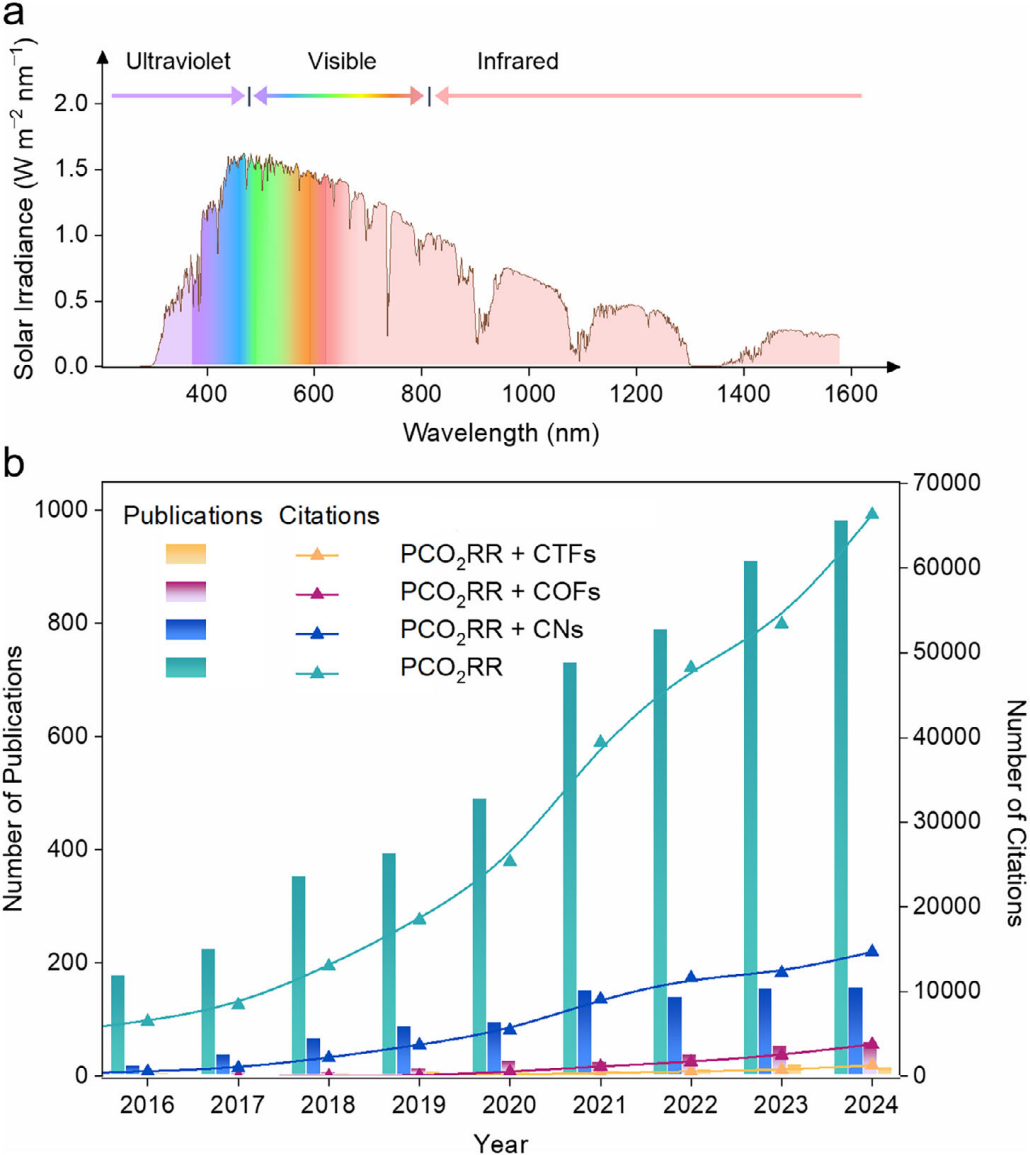
a) The solar spectrum consists of infrared, visible, and ultraviolet irradiation (below 380 nm). b) Publication and citation statistics of CO_2_ photoreduction reports for the topics “photocatalytic CO_2_ reduction reaction” and combined with the topics of “carbon nitrides”, “covalent organic frameworks”, and “covalent triazine frameworks”, respectively. (Web of Science, collected by timespan from 1970 to Jul 08, 2025).

Advancing the efficiency of solar‐driven CO_2_ conversion is an urgent priority in research. Sunlight is the primary energy source for driving CO_2_RR, particularly visible light (400–700 nm), accounting for a significant proportion of the solar spectrum. Materials that absorb in this range can effectively utilize light to enhance CO_2_ conversion efficiency. Compared to visible light, NIR light (> 780 nm) is also important as it can penetrate deep into materials to activate the photocatalyst, further boosting CO_2_ fixation efficiency (**Figure** **5**a).^[^ ^58^ ^]^ Accordingly, developing polymer photocatalysts with a broader absorption spectrum that covers both visible and near‐infrared light is essential for achieving more efficient CO2 conversion. Early research on the PCO_2_RR focused on inorganic materials, but organic polymers were of great interest due to their tunable light‐harvesting matrix, structural flexibility, and low cost. These organic semiconductors were also attractive in photocatalysis due to their superior ability to absorb visible light. Over the past decade, interest in carbon‐nitrogen‐based covalent materials for CO_2_ reduction has grown sharply, as evidenced by increasing publications and citations (Figure 5b). Among them, carbon nitrides in particular exhibit both stability and durability, ideal for long‐term photocatalysis. COFs and CTFs, with their versatile structures and large surface areas, enhance charge separation and light‐driven CO_2_ conversion. Compared to CTFs and COFs, CNs exhibit a significantly higher publication volume in the field of PCO_2_RR, which can be attributed to several factors. First, carbon nitrides are one of the earliest reported organic semiconductors, initially applied in photocatalysis (Figure 3). As a result, carbon nitrides had already been extensively studied and widely used before the widespread development of CTFs and COFs, laying a foundational role in CO_2_ conversion area. Second, C3N4 possesses a suitable bandgap (≈2.7 eV), enabling visible‐light‐driven reactions. Its conduction band position aligns well with the redox potential required for CO_2_ reduction. Additionally, it offers good chemical stability and contains abundant triazine and heptazine units that facilitate CO_2_ adsorption and activation. Furthermore, the simplicity and reproducibility of CNs synthesis have further contributed to its prominence as a widely studied material.^[^ ^31^, ^32^, ^35^ ^]^ In contrast, CTFs and COFs generally require complex monomer designs, multi‐step synthesis, and harsh reaction conditions. This trend signals rapid scientific progress and a bright future for these organic photocatalysts in global carbon reduction efforts.

## 3 Strategies to Enhance the PCO_2_RR Activity of Polymer‐Based Photocatalysts

**Figure 6:**
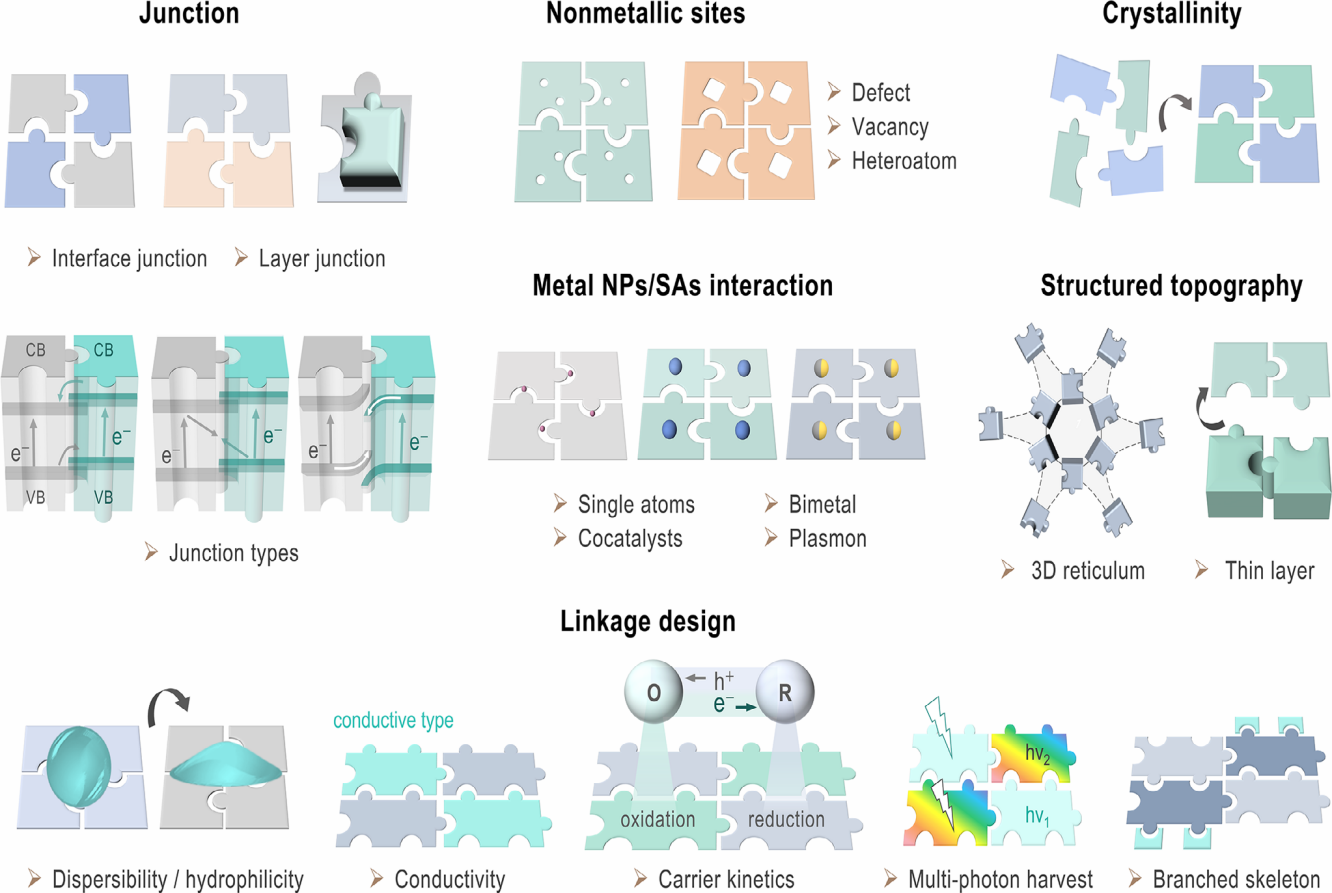
Schematic strategies to strengthen activity for PCO_2_RR via polymer‐based photocatalysts.

Although carbo‐nitrogen‐based covalent organic catalysts have garnered significant attention in photocatalysis, particularly for CO_2_ chemical fixation, achieving high activity, selectivity, and long‐term durability remains a major challenge. To address these issues, various approaches have been proposed and are generally grouped into two categories: synthesis design strategies and performance‐oriented targets. In terms of synthesis design strategies, greater emphasis should be placed on developing diverse approaches to create highly efficient photocatalysts capable of driving PCO_2_RR. These include junction engineering, incorporation of non‐metallic active sites, integration of metal nanoparticles (NPs) or single atoms (SAs), control of crystallinity, structural topography, and branched framework strategies. Such designs are aimed at enhancing CO_2_ conversion efficiency by generating abundant active sites, optimizing band structures, and tailoring surface properties to steer the reaction toward desired products. For instance, constructing heterojunctions facilitates charge separation and transport, thereby reducing recombination of photoexcited carriers. Engineering active sites through defect and vacancy creation, along with the integration of metal‐based components, can further improve charge separation and expand catalytic functionality. Enhanced crystallinity also provides well‐defined pathways for charge carriers, contributing to increased energy‐conversion efficiency. On the performance‐oriented side, key design objectives include improving dispersibility and hydrophilicity, enhancing electrical conductivity, accelerating carrier dynamics, and enabling multi‐photon harvesting. Given the inherent complexity of photochemical CO_2_ reduction, high‐performance catalysts must address multiple factors simultaneously, such as efficient light harvesting, charge generation and separation, strong CO_2_ adsorption affinity, multi‐electron transfer ability, and active site integration. Strategies like framework modification can enhance hydrophilicity and conductivity, ultimately boosting overall photocatalytic performance. These approaches aim to refine light capture, promote charge‐transfer kinetics, and optimize active sites for CO_2_ reduction. Taken together, the strategies, illustrated in **Figure** **6**, provide design strategies for advancing polymeric photocatalysts for PCO_2_RR. This multi‐angle perspective underscores the diverse and complementary methods currently employed in the field, aiming to achieve higher efficiency, improved selectivity, and extended operational stability.

### 3.1 Junction Design

#### 3.1.1 In‐Plane Junction Design

**Figure 7:**
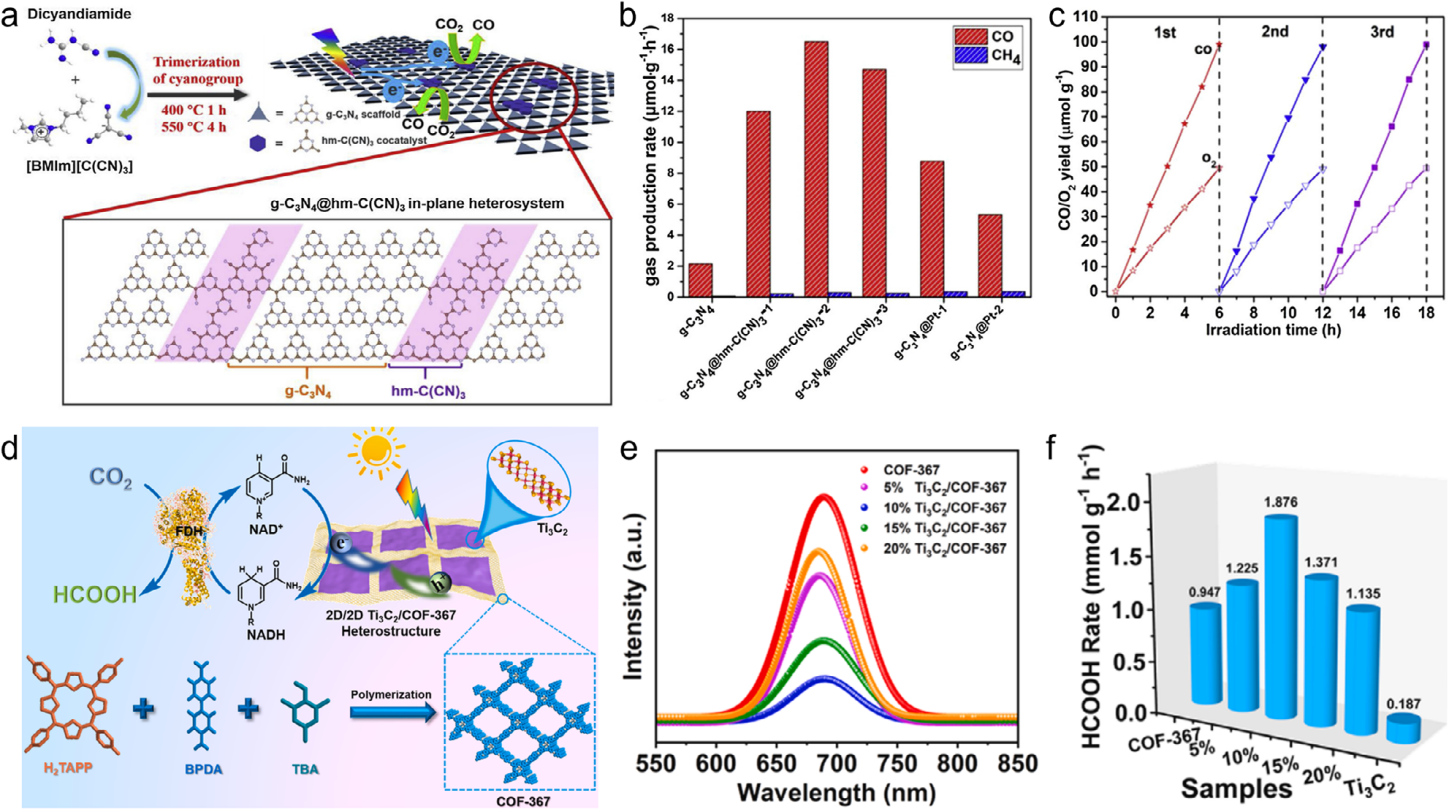
a) Synthesis of g‐C_3_N_4_@hm‐C(CN)_3_ via pyrolytic route. b) Gas production. c) Photostability tests.^[^ ^62^ ^]^ Copyright 2019 Elsevier B.V. d) Fabrication of Ti_3_C_2_/COF‐367 and corresponding NADH production for holoenzyme‐assisted co‐conversion to produce HCOOH. e) PL spectra. f) HCOOH generation rate.^[^ ^63^ ^]^ Copyright 2024 Elsevier B.V.

Structure‐defined in‐plane heterogeneous catalysts have been widely recognized for their capacity to direct charge flow within the plane, manage charge separation at interfaces.^[^ ^59^, ^60^, ^61^ ^]^ As an example, a metal‐free in‐plane junction combining g‐C_3_N_4_ (g‐CN) and half‐metallic COFs (C(CN)_3_) has been conceptually devised. The no‐slot link by covalent bonds from C(CN)_3_ and g‐CN forms a 2D π‐conjugated hybrid, realizing an unobstructed transfer of electrons from g‐CN to C(CN)_3_ via the internal electric field (**Figure** **7**a).^[^ ^62^ ^]^ The close integration of C(CN)_3_ with g‐CN may open up possibilities for achieving metal‐free photocatalysis aimed at PCO_2_RR. As shown in Figures 7b,c, this system achieves excellent CO production (16.5 µmol g^−1^ h^−1^), while maintaining high selectivity of over 98% and exhibiting exceptional photostability throughout the reaction. Further analysis indicated that the exceptional CO_2_ conversion performance stems from the following factors: (i) the half‐metallic nature of hm‐C(CN)_3_ catalysts provides an electric field across the 2D plane, facilitating charge transfer dynamics; (ii) the π‐conjugated feature with electron accumulation in the hm‐C(CN)_3_ part significantly enhances CO_2_ trapping and accumulation; and (iii) the carbonates and carboxylates formed on this in‐plane junction confirms an enhanced ability to activate CO_2_ through chemical processes.

#### 3.1.2 Layer Junction Design

The organic frameworks show tremendous potential in PCO_2_RR and warrant in‐depth exploration. However, excessive charge accumulation within the COFs diminishes their catalytic efficiency. Introducing abundant hetero‐interfaces is an effective way to address this, as it enables the transfer of the photo‐generated electrons, thereby enhancing photocatalytic efficiency. Beyond planar junctions mentioned above, the organic semiconductors are also ideal for constructing 2D/2D layered heterostructures for improving the generation of photo‐carriers. For instance, Hu et al. developed a photoconversion platform by synthesizing 2D/2D MXene/crystalline COF‐367 hybrid structures, effectively converting CO_2_ into HCOOH (Figure 7d).^[^ ^63^ ^]^ As shown, the steady‐state photoluminescence (PL) spectra of the samples provide clear evidence of efficient spatial charge separation (Figure 7e). Among all the samples, the peak intensity of the 10% Ti_3_C_2_/COF‐367 was the lowest, indicating a significant reduction in the radiative recombination of photogenerated carriers. At the same time, Figure 7f reveals that a Ti_3_C_2_ content of 10% is the optimal composition for the hybrids, achieving exceptionally high HCOOH yield (1.88 mmol g^−1^ h^−1^ with almost 100% selectivity).

#### 3.1.3 Junction Types

**Figure 8:**
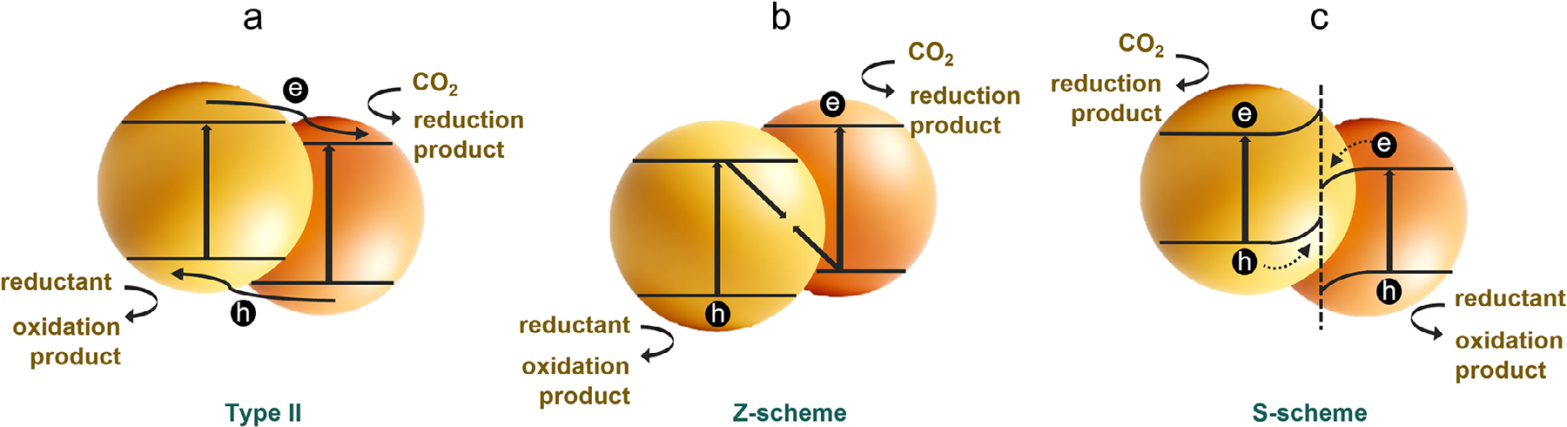
Illustration of type‐II, Z‐scheme, and S‐scheme designs according to charge transfer routines.

The type of junction in photocatalytic systems is critical to engineering the charge transport pathway, which directly impacts the overall performance of the PCO_2_RR. Different junction architectures have been meticulously developed to optimize the charge transfer behaviors, each offering unique advantages that vary with the particular photocatalytic systems being used. In this part, we mainly describe the classic heterosystems (**Figure** **8**): Type‐II, Z‐scheme, and S‐scheme heterojunctions. While both Z‐scheme and S‐scheme configurations aim to spatially separate photogenerated electrons and holes for enhanced redox capability, their mechanisms differ in key aspects. In a traditional Z‐scheme system, electrons in the conduction band of the reduction photocatalyst recombine with holes in the valence band of the oxidation photocatalyst, typically mediated by a solid or redox shuttle interface. In contrast, the S‐scheme establishes an internal electric field at the junction of two asymmetric semiconductors with staggered band alignment, which selectively preserves high‐energy electrons and holes by facilitating spatially directional charge migration. This configuration not only maintains strong redox potentials but also enhances photovoltage without external mediators.^[^ ^48^, ^49^ ^]^

##### Type‐II junction

In type‐II heterojunctions, the positions of the CB and the VB of the semiconductor are offset and staggered with those of the polymer‐based photocatalyst. This configuration, as shown in Figure 8a, enables effective spatial charge separation, driven by the internal electric field at the interface. However, this spatial separation often comes at the cost of reduced redox potential, which may limit the overall photocatalytic efficiency. Currently, several type‐II junctions have been reported for improving PCO_2_RR, including UiO‐66/polymer catalysts,^[^ ^64^ ^]^ TiO_2_/MnO_x_/polymer‐based composites,^[^ ^65^ ^]^ and BiOBr nanosheets (NSs)/polymer‐based heterojunctions.^[^ ^66^ ^]^ As an example, Xu et al. demonstrated the anchoring of CsPbBr_3_ quantum dots (QDs) onto porous g‐C_3_N_4_ NS with an NH_x_‐rich feature, forming a type‐II heterojunction through N‐Br chemical bonding.^[^ ^67^ ^]^ The N‐Br interaction at the interface promoted efficient charge separation and prolonged the lifetime of carriers. Under visible illumination, this photocatalyst demonstrated excellent durability and a superior CO output at 149 µmol g^−1^ h^−1^ in ACN/H_2_O mixture.

##### Z‐Scheme Junction

**Figure 9:**
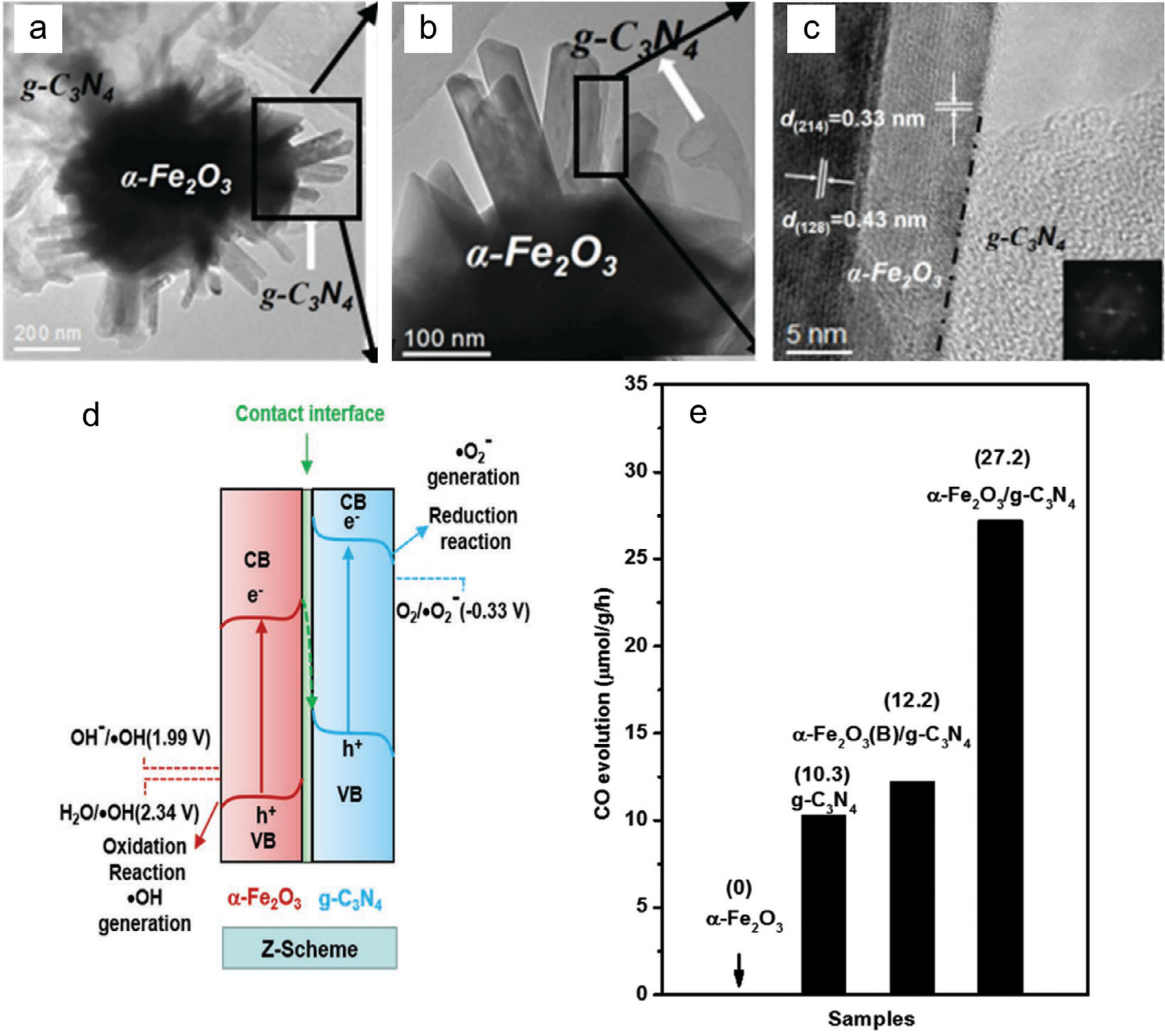
a–c) TEM and HRTEM images over *α*‐Fe_2_O_3_/PCN. d) Z‐scheme band energy alignment as the dominant factor in the photocatalytic pathway. e) Activity tests over PCN, *α*‐Fe_2_O_3_, and *α*‐Fe_2_O_3_/PCN.^[^ ^71^ ^]^ Copyright 2018 Wiley‐VCH Verlag GmbH.

The Z‐scheme junctions, initially inspired by natural photosynthesis processes, are designed to maintain high redox potentials while achieving spatial charge separation (Figure 8b). Unlike type‐II systems, Z‐scheme allows the combination of excited electrons from one semiconductor with generated holes from another, preserving high‐energy charge carriers on the respective semiconductors, which are critical for driving reduction and oxidation reactions. Since this concept was put forward, Z‐scheme designs have been categorized into traditional (liquid phase), indirect (all‐solid‐state), and direct systems.^[^ ^68^, ^69^ ^]^ Traditional Z‐schemes use redox couples in the liquid phase, while indirect systems employ a solid‐state electron mediator.^[^ ^70^ ^]^ In contrast, direct Z‐scheme systems enable charge transfer directly between semiconductors without the need for mediators. For instance, Wong et al. prepared *α*‐Fe_2_O_3_/PCN materials, a direct Z‐scheme junction with a hierarchical urchin‐like feature, using impregnation and hydrothermal methods.^[^ ^71^ ^]^ The TEM images reveal the close contact between *α*‐Fe_2_O_3_ and PCN, which is essential for the Z‐scheme process (**Figure** **9**a–d). The observed enhancement in CO_2_ absorption is due to the alkaline characteristics of iron as well as the synergistic function among the different components. As a result, the CO output rate of the *α*‐Fe_2_O_3_/PCN composites came up to 27.2 µmol g^−1^ h^−1^, significantly surpassing the pristine carbon nitrides (Figure 9e). Additionally, other systems, such as BiVO_4_/PCN,^[^ ^72^ ^]^ semiconductor/COF,^[^ ^73^ ^]^ Sn_2_S_3_/polymer,^[^ ^74^ ^]^ ZnO/polymer,^[^ ^75^ ^]^ and Cu_2_O/polymer composites,^[^ ^69^ ^]^ have also been developed to enhance CO_2_RR performance.

##### S‐Scheme Junction

**Figure 10:**
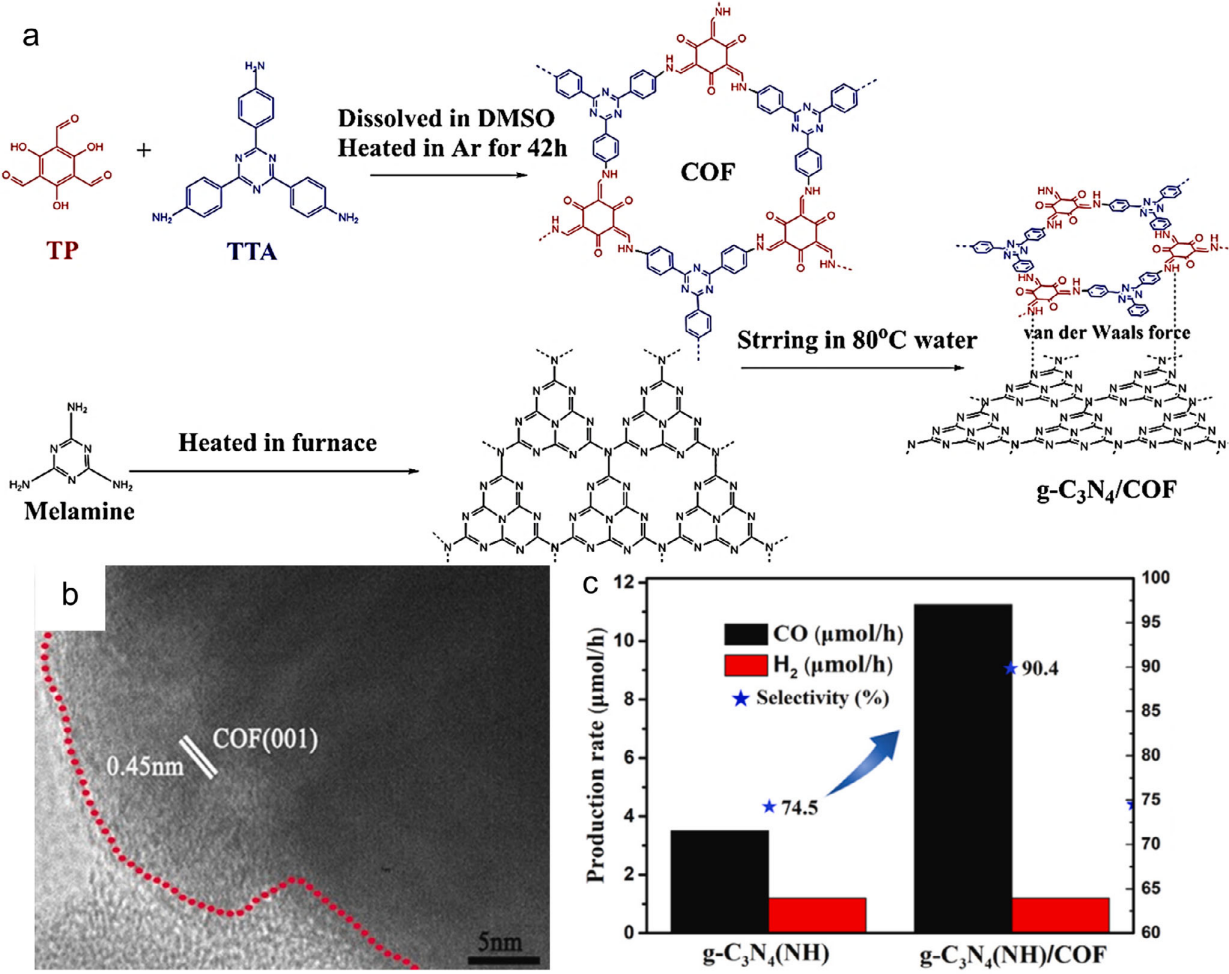
a) Illustration of the synthesis for the PCN/COF van der Waals junction. b) HRTEM image of PCN/COF. c) Photocatalytic yields using the catalysts of PCN/COF.^[^ ^76^ ^]^ Copyright 2021 Elsevier B.V.

To address the limitations inherent in the traditional and indirect Z‐scheme systems, Yu et al. introduced S‐scheme (step‐scheme) designs, which utilize the unique electronic properties of different semiconductors to enhance carrier separation and boost the photocatalytic efficiency of polymeric catalysts (Figure 8c). S‐scheme systems have been effectively constructed using various semiconductors, such as TiO_2_, InVO_4_, and COFs, in conjunction with polymer‐based photocatalysts. A noteworthy example is the van der Waals heterojunction developed by Ye et al., which was constructed by integrating defective polymeric materials with COFs through a self‐assembly approach. (**Figure** **10**a).^[^ ^76^ ^]^ The HRTEM image confirms the close interfaces between the two components (Figure 10b). Additionally, density functional theory (DFT) and experimental analysis indicated that the N vacancies within the framework facilitate electron transfer through the S‐scheme mechanism. The PCN/COF heterojunctions exhibited improved CO_2_‐to‐CO output. In conclusion, the π‐π interactions from these polymer matrixes and the well‐integrated interface greatly boost activity compared to single‐component systems (Figure 10c).

### 3.2 Nonmetallic Site Design

**Figure 11:**
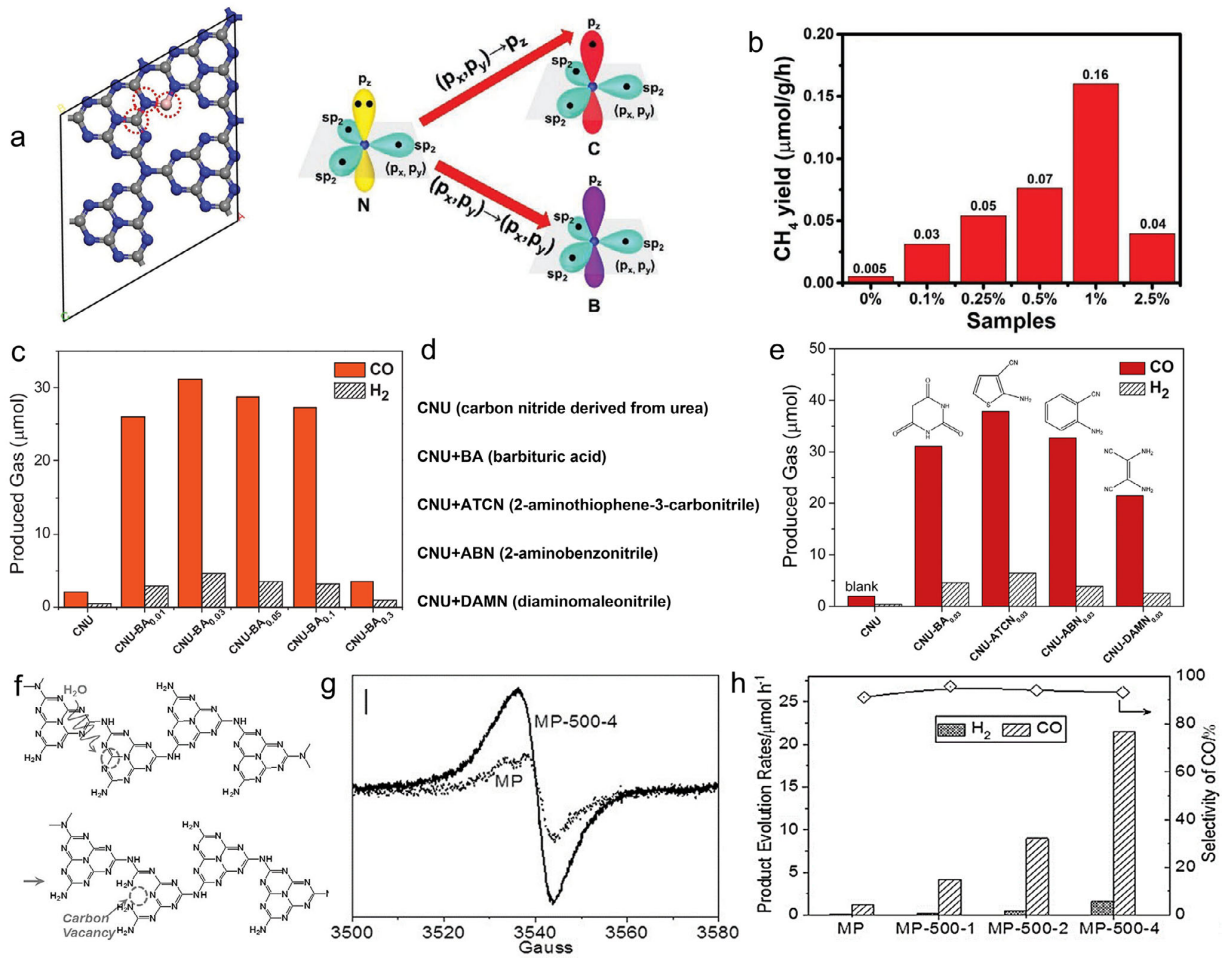
a) Electron excitation from N to B in B‐doped PCN is more favorable than from N to C in PCN. b) CH_4_ yield.^[^ ^22^ ^]^ Copyright 2019 Wiley‐VCH Verlag GmbH. c) Effect of BA content in the samples on CO_2_‐to‐CO output. d) CNU with other comonomers (e.g., ATCN, ABN, DAMN) modification. e) Performance of monomer‐modified CNU.^[^ ^77^ ^]^ Copyright 2015 Elsevier B.V. f) C vacancy formation by etching. g) EPR spectra confirming the presence of C vacancies. h) CO evolution.^[^ ^78^ ^]^ Copyright 2019 Wiley‐VCH Verlag GmbH.

Nonmetallic site design in polymeric photocatalysts, especially using heteroatoms or molecular doping, as well as vacancy/defective engineering, not only provides a highly effective approach for optimizing electronic properties, but also generates reactive sites during the PCO_2_RR. In this section, both elemental and molecular‐level doping types have shown significant promise in enhancing performance. As supported by DFT calculations, B‐doped PCN has been demonstrated to facilitate easier electron excitation from N to B, compared to the excitation from N to C in a pristine framework (**Figure** **11**a). This modification enhances carrier migration and localization with a significantly higher CH_4_ yield than pure PCN (Figure 11b).^[^ ^22^ ^]^ For molecular doping, another effective strategy is to incorporate the organic molecules into the π‐conjugated structure of carbon nitride to alter its intrinsic electronic properties. For example, the catalysts, derived from the copolymerization of barbituric acid (BA), have been validated by solid‐state ^13^C NMR tests, showing a signal at 94 ppm. This modification shifted the materials’ absorption spectra from 425 nm to 520 nm, indicating enhanced photon capture after engineering. Consequently, the barbituric‐acid‐modified materials exhibited a high CO production (31.1 µmol h^−1^), much higher than the pristine (Figure 11c).^[^ ^77^ ^]^ Further, changing the units (e.g., 2‐aminothiophene‐3‐carbonitrile, ATCN; 2‐aminobenzonitrile, ABN; diaminomaleonitrile, DAMN) to polymerize with urea, the obtained samples with different doping at the molecular level also show remarkably enhanced activity for CO_2_ conversion (Figure 11d,e). In particular, the ATCN‐doped catalysts showed high efficiency with a CO formation of 37.9 µmol, 19 times that of the pristine. The results show that incorporating comonomers to create nonmetallic active sites significantly enhances PCO_2_RR performance, offering organic protocols to tailor carbon nitrides for CO_2_ reduction. Additionally, vacancy/defective engineering within the PCN matrix have been demonstrated its effective in increasing the catalytic activity. These vacancies act as active sites that can trap and stabilize electrons, thus facilitating the photocatalytic reduction of CO_2_. One prominent example involves the introduction of C vacancies in PCN (MP‐CVs) through a steam‐etching process (Figure 11f). Electron paramagnetic resonance (EPR) spectra confirmed the presence of C vacancies, which serve as centers for electron trapping and provide long‐lived carriers necessary for the reduction reaction (Figure 11g).^[^ ^78^ ^]^ The presence of these vacancies was also shown to improve CO_2_ adsorption, as evidenced by the larger and higher‐temperature‐shifted CO_2_ temperature‐programmed desorption (CO_2_‐TPD) peaks compared to those of pristine PCN. The enhanced CO_2_ adsorption and lowered activation energy barrier led to a significantly higher CO evolution rate, which was 45 times higher than that of PCN (Figure 11h). Similarly, N vacancies not only boost CO_2_ reduction by promoting exciton dissociation into free charges but also act as active sites for CO_2_ adsorption and activation.^[^ ^79^ ^]^

### 3.3 Crystallinity Enhancement

**Figure 12:**
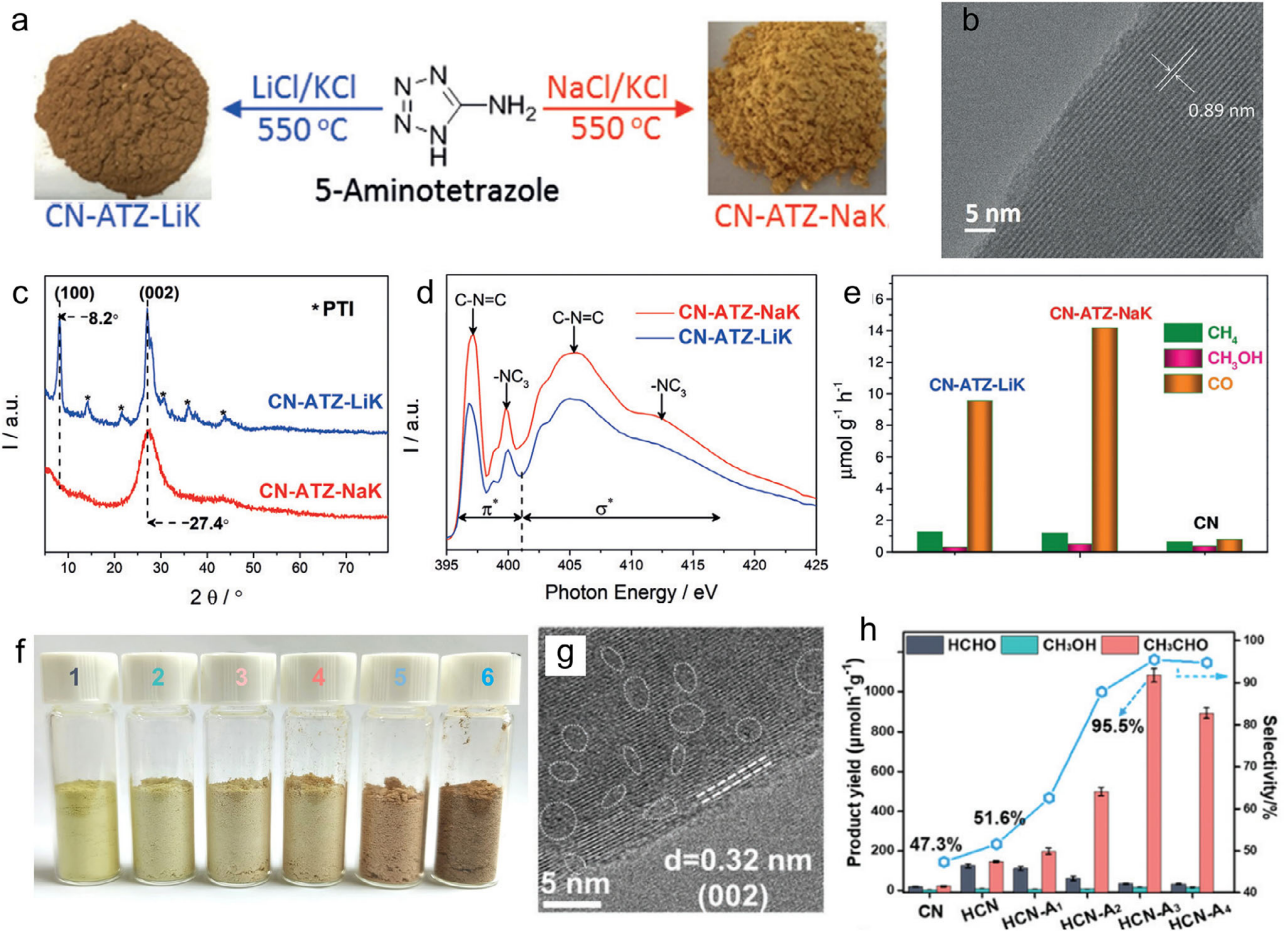
a) Synthesis of PHI by using 5‐amino tetrazole. b) HRTEM images. c) XRD pattern of the constructed PHI. d) N‐K edge XANES of the samples. e) Photocatalytic activity over the samples.^[^ ^80^ ^]^ Copyright 2019 Wiley‐VCH Verlag GmbH. f) Digital pictures of the samples: CN (1), HCN (2), HCN‐A_1_ (3), HCN‐A_2_ (4), HCN‐A_3_ (5), and HCN‐A_4_ (6). g) HRTEM images revealing clear lattice fringes of HCN‐A_3_. h) CO_2_ reduction rates and the products selectivity over the samples.^[^ ^81^ ^]^ Copyright 2022 The Royal Society of Chemistry.

Enhanced crystallinity minimizes bulk defects, narrows the band gap, and improves charge transfer—all important for efficient PCO_2_RR. Recently, Wang et al. demonstrated the synthesis of crystalline carbon nitride (CCN) from 5‐aminotetrazole (ATZ) using salt melt method (**Figure** **12**a).^[^ ^80^ ^]^ The constructed poly‐heptazine‐imide (PHI) CCN, with a high degree of crystalline feature, was confirmed through HRTEM image (clear lattice fringes observed) and XRD patterns (sharp peaks observed) (Figure 12b,c). Moreover, electron energy loss spectra (EELS) indicated a positive shift in the binding energy of the C‐*K* edge in the CN‐ATZ‐NaK structure, reflecting extended conjugation and enhanced crystallinity. As shown in Figure 12d, both samples showed similar spectra in the π* and σ* regions, suggesting that different salt melt modifications primarily influence local packing and grain boundaries rather than altering the core structure of polymers. These enhancements improved optical absorption and electronic properties, significantly boosting PCO_2_RR performance (Figure 12e). Another method to achieve high crystallinity in PCN involves hydrothermal pre‐treatment. Wang and colleagues employed this approach to create a locally crystallized PCN designed for CO_2_RR to acetaldehyde (CH_3_CHO).^[^ ^81^ ^]^ In this work, the PCN sample modified with amino‐2‐propanol (AP), named HCN‐A, was synthesized through hydrothermal treatment. (Figure 12f). These samples were modified with different AP concentrations: 0.1, 0.2, 0.5, and 1.0 mL, denoted as HCN‐A_1_, HCN‐A_2_, HCN‐A_3_, and HCN‐A_4_, respectively. Taking HCN‐A_3_ as an example, HRTEM images revealed locally crystallized structures with distinct lattice fringes. The synthesized samples of high crystallinity promoted the hydrogenation of the *OCCHO intermediate while inhibiting the formation of *CHO to *CH_2_O species, which facilitated the proton‐coupled electron transfer process required to produce CH_3_CHO. Consequently, the locally crystallized HCN‐A_3_ demonstrated an outstanding CH_3_CHO production rate of 1083.5 µmol g^−1^ h^−1^, making it a promising candidate for advanced CO_2_ photocatalysis.

### 3.4 Metal NPs/Complexes/Single‐Atom Based Polymeric Materials

The metal NPs/single‐atom‐based polymeric photocatalysts can be classified into four main parts: metal NPs/polymer, metal complexes/polymer, bimetallic NPs/polymer, and metal single‐atom/polymer catalysts. From the perspective of enhancing photocatalysis, the mechanisms can be broadly classified into three categories: the cocatalyst effect, the plasmonic effect, and the contribution of active sites.^[^ ^82^, ^83^ ^]^ Thus, these modifications that contribute to improved PCO_2_RR performance are discussed in the Subsections below: 3.4.1. Cocatalyst Design, 3.4.2. Plasmonic Effect, 3.4.3. Hosting Metal Complexes on Polymeric Materials, and 3.4.4. Single‐Metal Atom Supported Polymeric Materials.

#### 3.4.1 Cocatalyst Design

**Figure 13:**
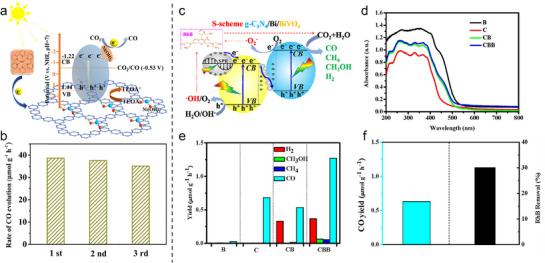
a) Illustration of the photocatalytic CO_2_ reduction and b) CO evolution over Ni(OH)_2_/CTF‐1 (Ni_0.5_‐CTF‐1).^[^ ^84^ ^]^ Copyright 2021 Elsevier Inc. c) Photocatalytic carbon cycling over the ternary S‐scheme of g‐C_3_N_4_/Bi/BiVO_4_. d) UV–Vis DRS. e) Gaseous evolution in water vapor. f) CO yield and RhB removal over sample CBB.^[^ ^85^ ^]^ Copyright 2020 Elsevier B.V.

One common strategy to increase the photocatalytic performance of PCN is to deposit metal NPs onto its surface. This method leverages the different work functions between the metal NPs and PCN, leading to band bending at the interface and forming a Schottky layer, which directionally drives charge migration. Recently, Bi et al. exemplified this approach by constructing a Ni(OH)_2_‐modified CTF‐1 composite (termed Ni(OH)_2_‐CTF‐1), where Ni(OH)_2_ functions as a cocatalyst ensemble for superior CO_2_ conversion.^[^ ^84^ ^]^ This work offers a novel pathway for developing noble‐metal‐free cocatalysts on CTF and deepening insights into PCO_2_RR mechanisms (**Figure** **13**a,b). Among them, the optimal Ni(OH)_2_‐CTF‐1 materials (named Ni_0.5_‐CTF‐1, loading ratio at 0.5 wt%) achieved an optimal CO generation at 38.66 µmol g^−1^ h^−1^—an improvement of ≈33 times over bare CTF‐1. This performance improvement arises from the directional separation and transfer of carriers facilitated by the addition of the Ni(OH)_2_ cocatalyst.

#### 3.4.2 Plasmonic Effect

The design of plasmonic catalysts is a significant advancement in photocatalysis, especially for improving the efficiency of PCO_2_RR under visible light. Plasmonic catalysis leverages the unique properties of metal NPs, such as localized surface plasmon resonance (LSPR), which can enhance light absorption and charge separation efficiency. A noteworthy example is the ternary g‐C_3_N_4_/Bi/BiVO_4_ photocatalyst, which was developed to address the challenges in carbon cycling by combining aerobic oxidation and anaerobic reduction processes (Figure 13c). The preparation of this photocatalyst involves the in situ formation of Bi NPs, which are uniformly dispersed within the g‐C_3_N_4_/BiVO_4_ matrix. In Figure 13d, the UV–Vis diffuse reflectance spectra (UV–Vis DRS) show enhanced visible light absorption due to the plasmonic effect generated by Bi NPs. In water vapor systems, this catalyst shows a substantial increase in CO yield while also being effective in Rhodamine B degradation (Figure 13e,f). This bifunctional capability makes the g‐C_3_N_4_/Bi/BiVO_4_ promising candidates for applications toward artificial carbon cycling.^[^ ^85^ ^]^

#### 3.4.3 Hosting Metal Complexes on Polymeric Materials

**Figure 14:**
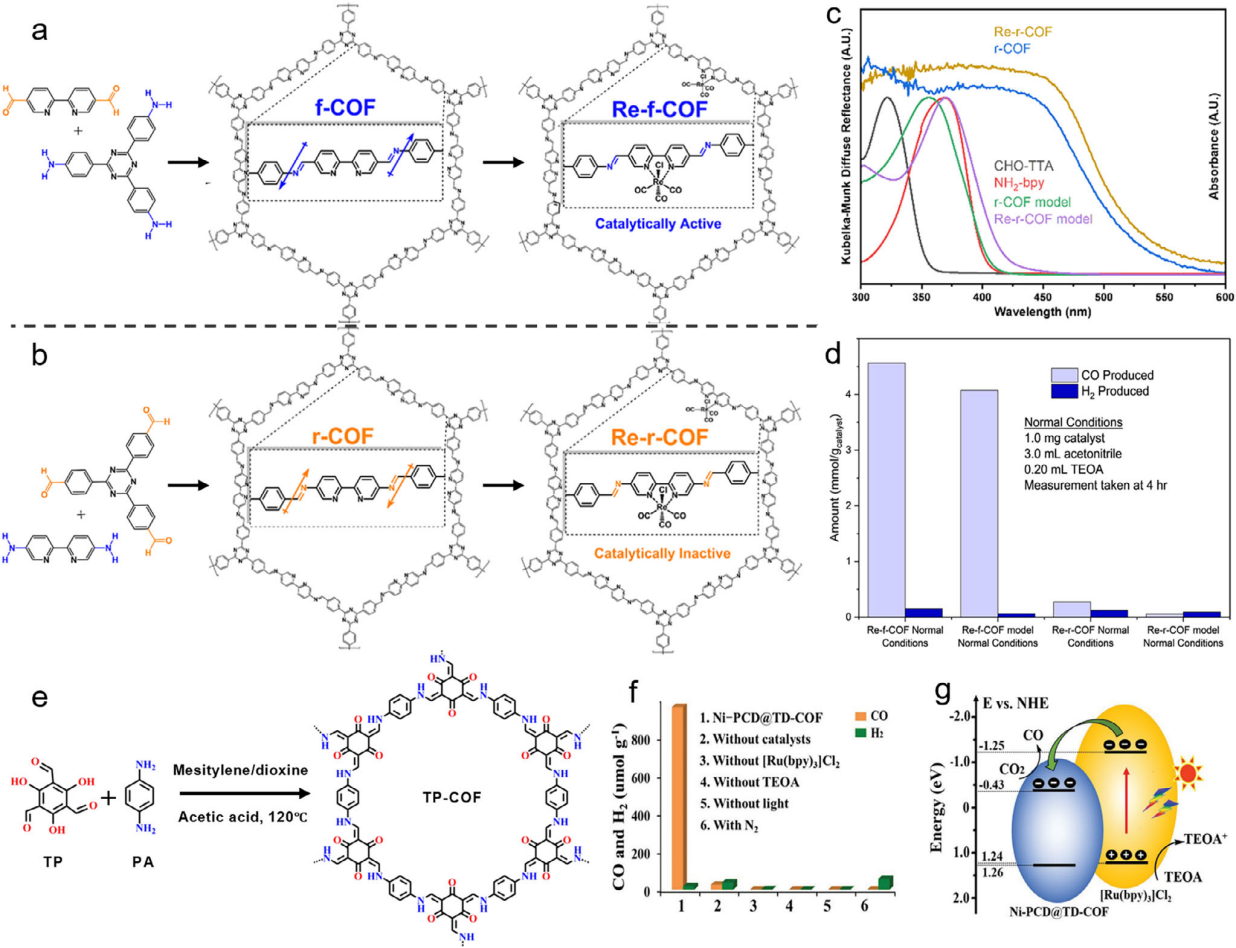
a,b) Synthesis of f‐COF, Re‐f‐COF, r‐COF, and Re‐r‐COF. c) UV–Vis spectra. d) CO production.^[^ ^86^ ^]^ Copyright 2024 American Chemical Society. e) Synthesis of TP‐COF. f) Photocatalytic CO_2_‐to‐CO conversion over NiPCD@TD‐COF under different conditions. g) Energy‐level diagram over [Ru(bpy)_3_]Cl_2_ and Ni‐PCD@TD‐COF.^[^ ^52^ ^]^ Copyright 2020 WILEY‐VCH Verlag GmbH.

Hosting metal complexes on polymeric materials is a commonly used grafting technique to functionalize the organic structures with catalytically active metal species. The parent COF framework were found to play a crucial role in modulating the activity of the embedded metal complexes toward PCO_2_RR. Several studies demonstrated that these hybrid catalysts can efficiently reduce CO_2_ to form CO under light illumination, not only showing the capability of COFs as the platform to incorporate metal centers and but also the stability of the metal complexes is largely enhanced after being incorporated into COFs.^[^ ^45^, ^52^, ^86^ ^]^ For example, Huang and colleagues revealed that Re‐f‐COF and Re‐r‐COF differ in ICT (intramolecular charge transfer) efficiency, attributed to distinct imine orientations—serving as an “ICT Tesla valve” to control charge directionality. **Figure** **14**a,b illustrates the synthesis of f‐COF and r‐COF with embedded Re centers (Re(CO)_5_Cl). This integration enhances photocatalytic performance by offering a stable and efficient environment for Re active sites that work for excellent CO_2_ conversion. In Figure 14c, UV–Vis DRS displays the light absorption spectra of various COF models. After incorporating molecular Re species into the COF backbone, both Re‐f‐COF and Re‐r‐COF exhibit enhanced light absorption. This improved absorption enables more efficient solar energy utilization, thereby boosting photocatalytic CO_2_ fixation efficiency.^[^ ^86^ ^]^ However, only Re‐f‐COF exhibited high activity, achieving a CO production of 6.3 ± 1.2 mmol/g_catalyst_ over 8 h. In contrast, its isomer, Re‐r‐COF, showed negligible activity (0.30 ± 0.05 mmol/g_catalyst_) (Figure 14d). The differences in PCO_2_RR behavior originated from the polarized characteristics of the imine linkage, which plays a decisive role in directing the ICT pathway. Under photoexcitation, the bipyridine group acts as an electron acceptor in the forward imine COF (f‐COF), while acting as an electron donor in the reverse configuration (r‐COF). Consequently, the Re‐functionalized f‐COF demonstrates superior performance during CO_2_ conversion. For another work, Figure 14e provides a schematic overview of the synthesis process for TP‐COF, a COF designed to host metal‐based complexes.^[^ ^52^ ^]^ In this example, the COF is coupled with a Ni phthalocyanine derivative (Ni‐PCD) to form Ni‐PCD@TD‐COF hybrids, which is further integrated with [Ru(bpy)_3_]Cl_2_ to enhance photoreduction activity (Figure 14f). The efficient electron transfer from the Ru complex to the COF structure facilitated effective CO_2_ conversion in the visible light region. Figure 14g provides details of the energy‐level diagram, showing how electrons are transferred from the [Ru(bpy)_3_]Cl_2_ complex to the Ni‐PCD@TD‐COF. As indicated, the integration of metal complexes with COFs opens the avenues for developing advanced photocatalysts with high efficiency and stability, essential for achieving sustainable energy solutions.

**Figure 15:**
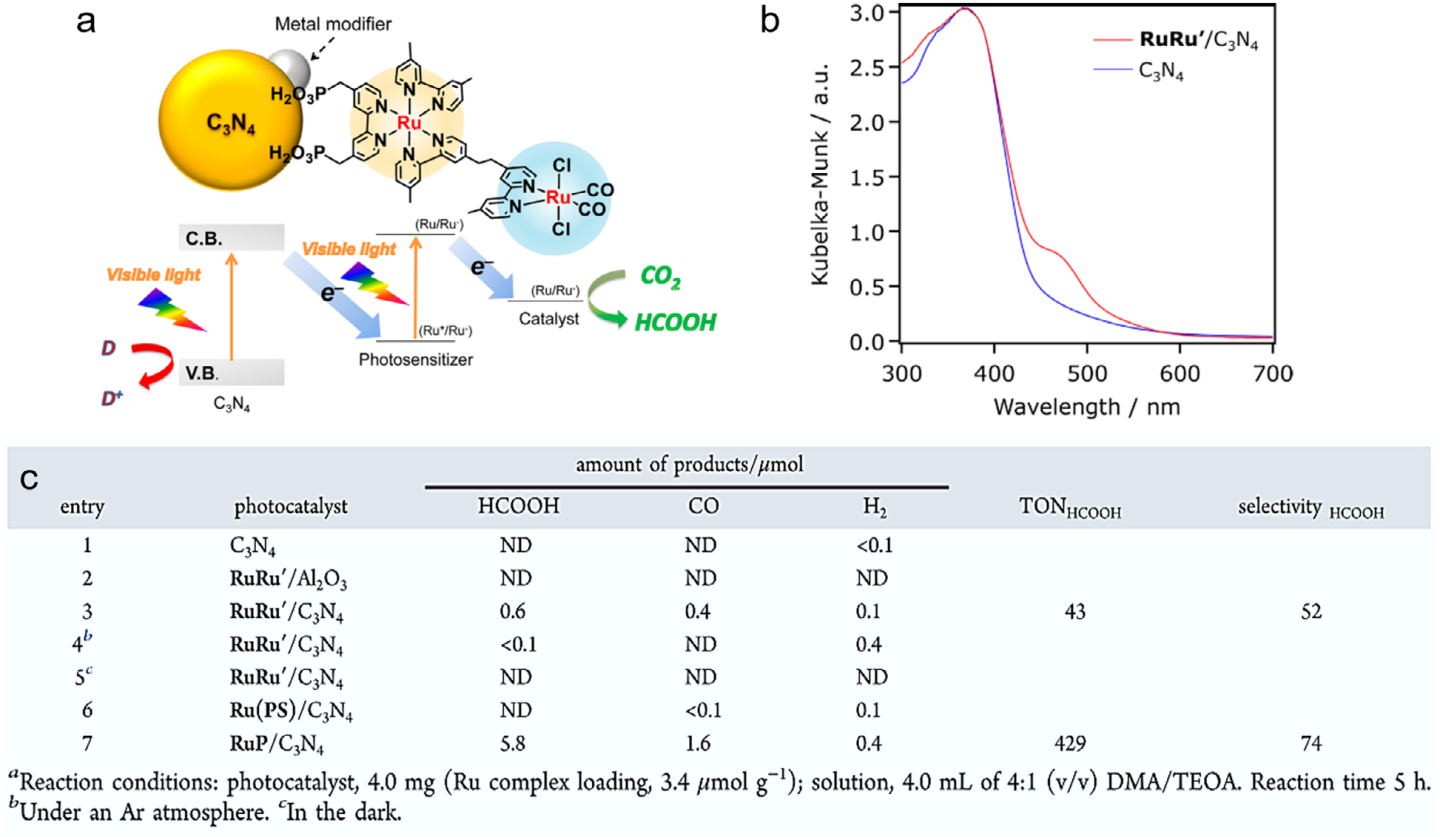
a) The nature‐inspired CO_2_ conversion system by copolymerizing semiconductors with a Ru metal complex. b) UV–vis diffuse reflectance spectra of Ru‐complex‐modified C_3_N_4_ and pristine C_3_N_4_. c) Photocatalytic CO_2_ conversion over diversely modified C_3_N_4_.^[^ ^89^ ^]^ Copyright 2016 American Chemical Society.

In PCO_2_RR systems, polymeric materials have been widely applied due to their excellent visible‐light responsiveness, thermal stability, and convenient synthesis. However, their intrinsic drawbacks—namely the narrow visible‐light absorption range and high exciton recombination rate—limit their practical photocatalytic efficiency. To address these limitations, dye‐sensitization strategies have been extensively adopted to enhance light‐harvesting capabilities.^[^ ^87^, ^88^ ^]^ For example, by co‐polymerizing carbon nitride materials with Ru complexes, the RuP‐sensitized C_3_N_4_ photocatalyst enabled CO_2_ reduction under visible light (**Figure** **15**a,b).^[^ ^89^ ^]^ UV–Vis absorption spectra confirmed the successful integration of the dye, with the co‐polymerized C_3_N_4_ maintaining similar adsorption behavior to pristine semiconductor, while the UV−vis absorption spectra revealed improved visible‐light absorption. As shown in Entry 1 of Figure 15c, pristine C_3_N_4_ exhibited no catalytic activity under the reaction conditions. When combined with the binuclear Ru complex RuRu’, CO_2_ reduction proceeded under visible‐light irradiation, yielding HCOOH (0.6 µmol) and CO (0.4 µmol) with selectivities of 52% and 38%, respectively (Entry 3). In Entry 4 and 5, control experiments confirmed that no products were formed in the absence of either CO_2_ or light. When C_3_N_4_ was co‐polymerized with the mononuclear Ru(PS) complex (serving solely as a photosensitizer model), the resulting Ru(PS)/C_3_N_4_ catalyst produced no detectable product (Entry 6). In contrast, the resulted RuP/C_3_N_4_ materials were capable of generating both HCOOH and CO (Entry 7), with higher product yields than the RuRu’/C_3_N_4_ system. These studies underscore that coupling the semiconductors with appropriate dye molecules can significantly extend the photoresponse range and improve charge separation efficiency via directional charge transfer from the excited dye to the carbon nitride matrix.

#### 3.4.4 Single‐Metal Atom Supported Polymeric Materials

Recently, single‐atom catalysts (SACs) using polymeric materials as support applied in thermal catalysis, electrocatalysis, and photocatalysis have garnered significant attention. The distinctive properties include:^[^ ^90^, ^91^, ^92^ ^]^ i) Isolated metal centers with atomic dispersion achieve 100% active atom utilization. ii) Low‐coordinated metal centers can be tuned with different coordination environments. iii) Quantum size effects of electron confinement at the local centers facilitate an in‐depth exploration of catalysis. iv) Strong and unique metal‐support interactions in the well‐defined organic framework show great potential in enhancing reaction rates and selectivity across the chemical processes.

**Figure 16:**
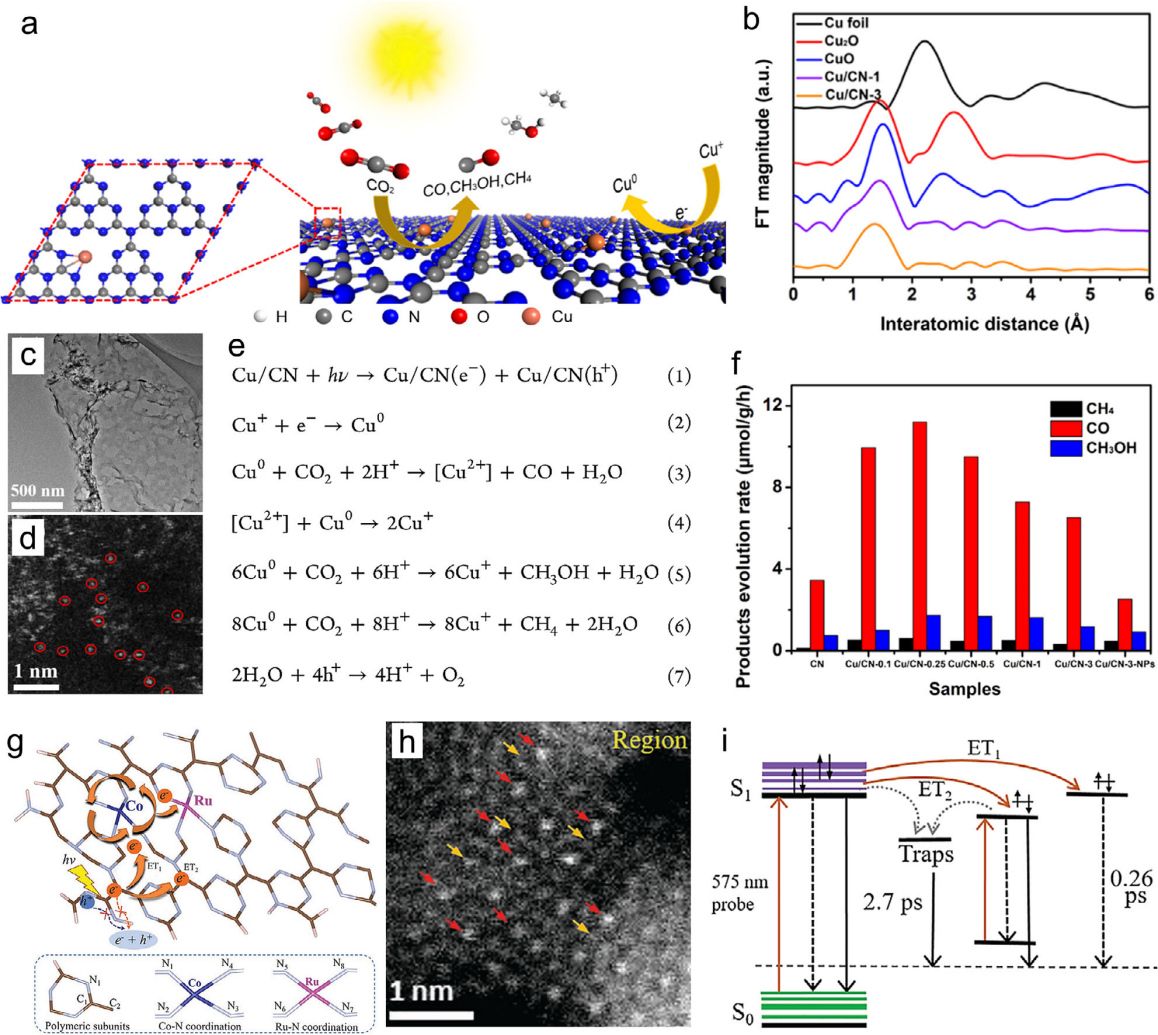
a) Illustration of the Cu/CN for PCO_2_RR. b) FT‐EXAFS of Cu *K*‐edge analysis.^[^ ^93^ ^]^ Copyright 2020 American Chemical Society. c,d) TEM and HADDF‐STEM images of Cu/CN‐3. e) Charge transfer pathways over Cu/CN. f) Photoreduction activity. g) Illustration of CO_2_ adsorption and carriers transfer process in CoRu‐HCNp. ET_1_ and ET_2_ show electron transfer from C_3_N_4_ to dual sites and electron capturing within the π conjugated units. h) HAADF‐STEM image of CoRu‐HCNp, 1 nm. i) Schematics of dynamics on CoRu‐HCNp.^[^ ^92^ ^]^ Copyright 2021 Wiley‐VCH GmbH.

For instance, in **Figure** **16**a, atomic copper‐modified PCN (Cu/CN) materials were prepared through preorganization and condensation methods, resulting in uniformly dispersed Cu single atoms (SAs) on the nanosheet. EXAFS and HADDF‐STEM analysis demonstrated a homogeneous Cu distribution on the polymeric substrates (Figure 16b–d). Additionally, the coordination environment of the SAs has been revealed with an obvious peak ≈1.5 Å, corresponding to the Cu–N bond.^[^ ^93^ ^]^ The Cu SAs act as electron collectors and reactive centers for CO_2_ activation. As displayed in Figure 16e, equations illustrate the charge transfer pathways in Cu/CN hybrid materials. Pure CN exhibits relatively low performance for producing CH_3_OH (0.76 µmol g^−1^) and CH_4_ (0.12 µmol g^−1^) upon a 1‐h test (Figure 16f). For comparison, a series of Cu/CN‐x (x represents Cu contents) materials were developed and tested. Particularly, Cu/CN‐0.25 demonstrated the highest CH_3_OH and CH_4_ production (1.75 and 0.61 µmol g^−1^). As another example, the atomically Co‐Ru bimetal dopants have been introduced into PCN through a self‐seeded process.^[^ ^92^ ^]^ In Figure 16g, within the π conjugated units, ET_1_ and ET_2_ show the photogenerated electron transfer from C_3_N_4_ to dual Co‐Ru sites and electron capturing by surface states, respectively. Further, the HAADF‐STEM analysis confirmed the atomically dispersed Ru and Co species within the PCN (Figure 16h). The coordination environment of these SAs was further clarified through EXAFS analyses, which revealed the presence of Co–N and Ru–N bonds, confirming the successful incorporation of SAs into the PCN framework. Figure 16i illustrates the dynamic carrier transfer process on CoRu‐HCNp during CO_2_ photoreduction. Benefiting from the synergistic interaction between Co and Ru SAs, the functional photocatalyst demonstrates selectivity in CO_2_ adsorption and improved reduction efficiency.

**Figure 17:**
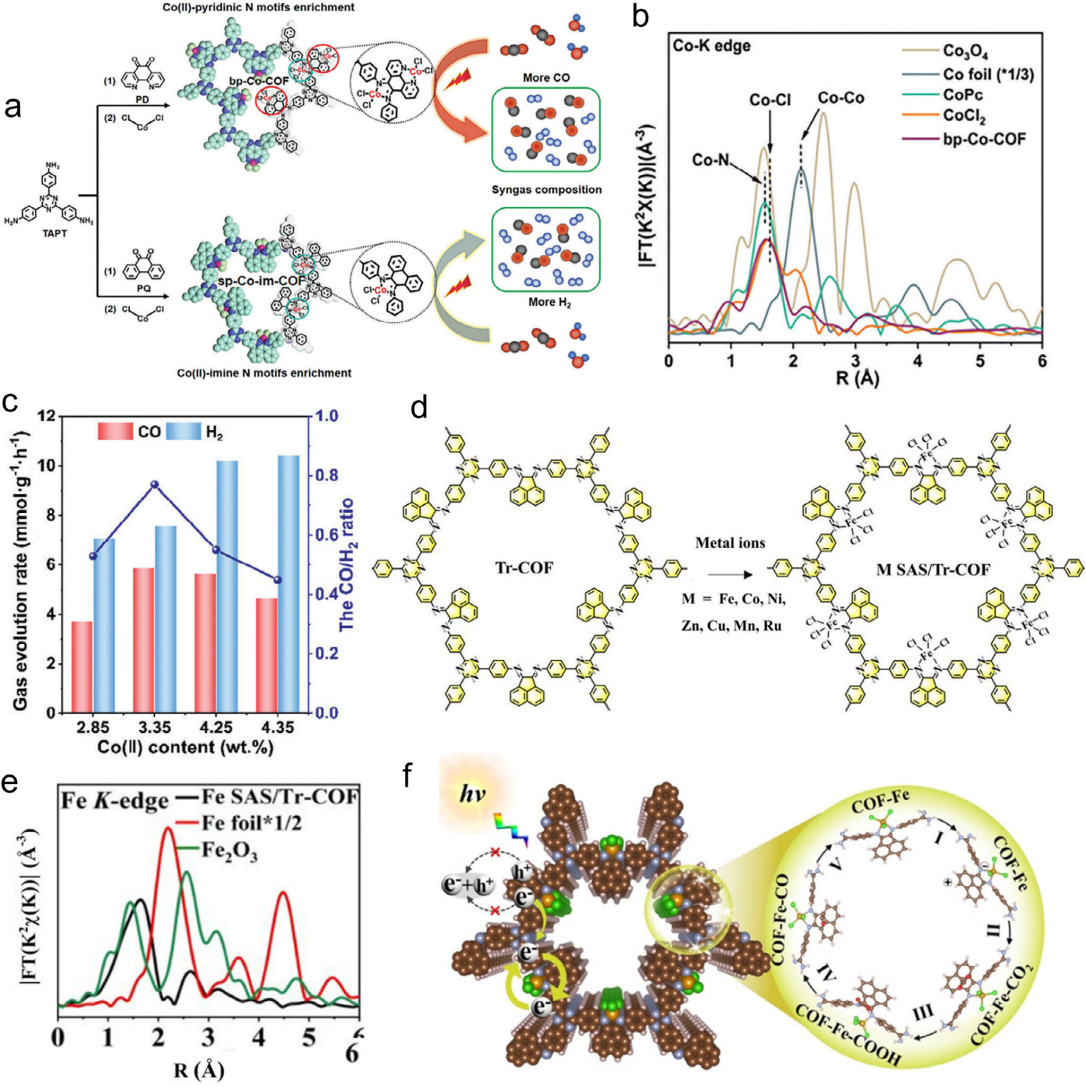
a) Schematic depicting the relationship between Co(II)‐pyridine N and/or Co(II)‐imine N motifs enriched bp‐Co‐COF, sp‐Co‐im‐COF used for CO_2_ reduction. b) Co *K*‐edge of EXAFS spectra. c) Production of syngas using bp‐Co‐COF_x_ samples with different Co content.^[^ ^94^ ^]^ Copyright 2024 Wiley‐VCH GmbH, d) Synthesis of Fe SAS/Tr‐COFs. e) EXAFS spectra of Fe SAS/Tr‐COFs. f) Proposed PCO_2_RR mechanism.^[^ ^95^ ^]^ Copyright 2022 American Chemical Society.

The exploration of metal‐single‐atom interaction within COFs is further elaborated in **Figure** **17**a. The depiction highlights the critical role of Co(II)‐pyridine N motifs and Co(II)‐imine N motifs in the design of bp‐Co‐COF and sp‐Co‐im‐COF structures, emphasizing how these motifs contribute to enhanced photoreduction performance. The Co *K*‐edge Fourier transform (FT) k^3^‐weighted EXAFS spectra provide detailed information on the coordination of the SAs within the frameworks (Figure 17b). This work indicated that the ability to manipulate coordination environments is crucial for tuning the photocatalytic efficiency of the COFs. Figure 17c shows the generated syngas (CO/H_2_ mixture) over bp‐Co‐COF_x_ samples with varying Co contents. This flexibility in adjusting Co content for optimizing CO_2_ conversion processes showcases the adaptability of the materials. These findings are supported by recent studies that demonstrate the role of bipolaronic motifs in creating spatially separated catalytic sites, which are key for syngas photosynthesis from CO_2_.^[^ ^94^ ^]^ Further, the synthesis scheme of Fe SAS/Tr‐COFs (Figure 17d) outlines the preparation methodology, demonstrating how these materials are engineered. The Fe *K*‐edge FT k^3^‐weighted EXAFS spectra of Fe SAS/Tr‐COFs (Figure 17e), along with reference samples, provide local coordination information of Fe SAs. This mechanism highlights how active sites accelerate reaction kinetics, thereby enabling efficient CO_2_ conversion. The deeper insights into the reaction pathways further supported by research focused on engineering single‐atom active sites on COFs to promote PCO_2_RR process (Figure 17f).^[^ ^95^ ^]^

**Figure 18:**
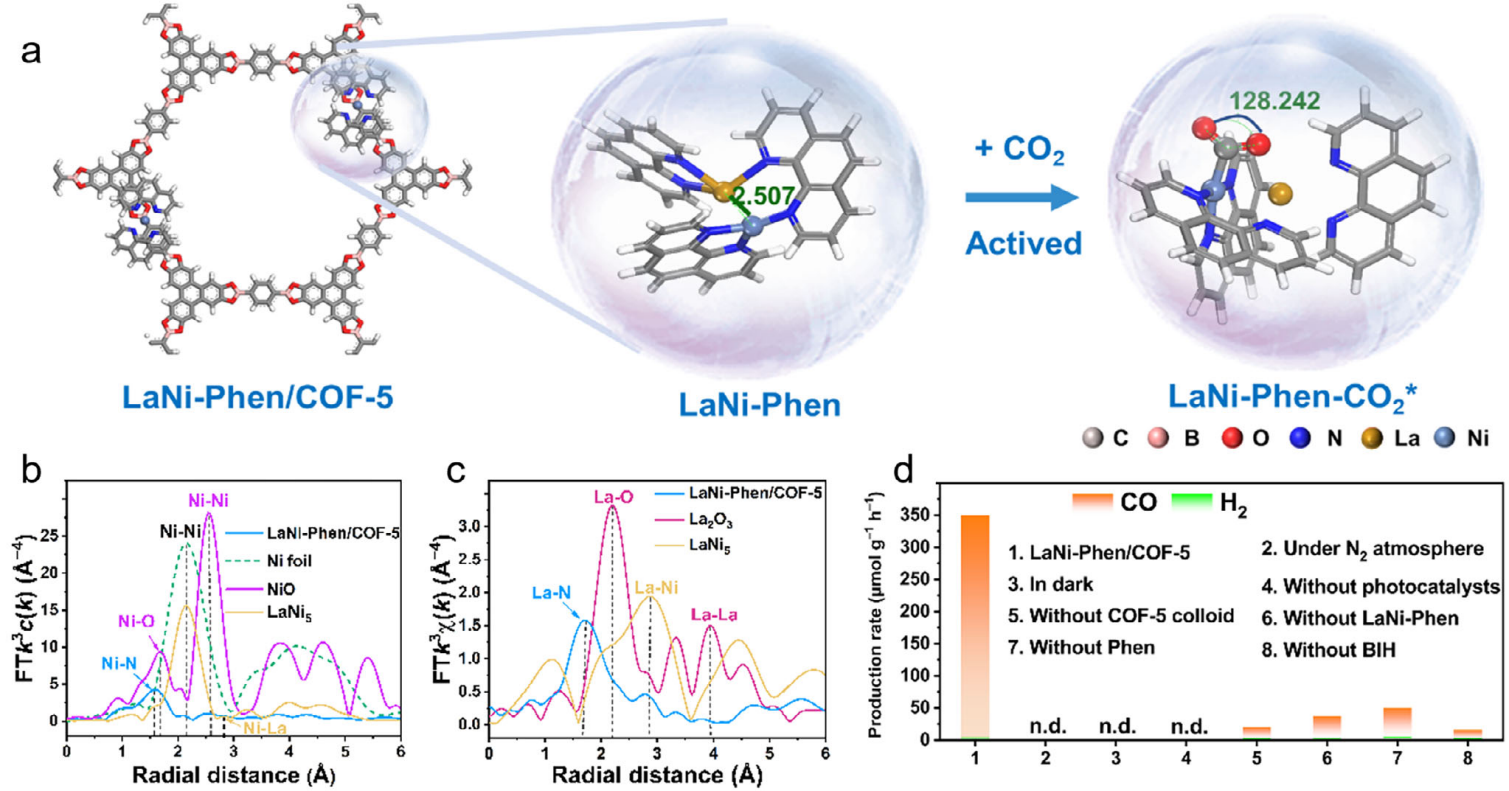
a) Structure diagram of the photocatalyst model with diatomic La and Ni sites for CO_2_ photoreduction. b,c) Structural characterization by Ni K‐edge and La L‐edge extended X‐ray absorption. d) Photocatalytic CO_2_ reduction over LaNi‐Phen/COF‐5 under varied conditions.^[^ ^96^ ^]^ Copyright 2023 The Author(s).

Recent studies have highlighted that bimetallic coordination sites can offer synergistic catalytic effects by implementing dual‐atom level orbital modulation. In particular, La‐Ni bimetallic configurations anchored in COFs have demonstrated high selectivity and activity for photocatalytic CO_2_‐to‐CO conversion. As illustrated in **Figure** **18**a, the heteronuclear La–Ni paired system via charge polarization and cooperative binding enables site‐specific activation of CO_2_. The structural nature of the sites in the LaNi‐Phen/COF‐5 was clearly resolved by X‐ray absorption spectroscopy, with the dominant Ni‐N and La‐N coordination environments, providing evidence for dual‐site stabilization within the COF scaffold (Figure 18b,c). The resultant material exhibited significantly enhanced CO evolution compared to control groups lacking La, Ni, or the COF scaffold (Figure 18d).^[^ ^96^ ^]^ Although not specifically designed for CO_2_ reduction, the photo‐induced construction of heteronuclear dual‐atom catalysts, such as La–Ni pairs, has been explored in other systems as well. For example, a recent study demonstrated that visible‐light‐triggered assembly of dual‐atom sites within conjugated porous scaffolds of on CNs can promote cooperative activation of small molecules, suggesting a promising avenue for multi‐metal supported photocatalyst engineering.^[^ ^97^ ^]^

### 3.5 Structured Topography Design

#### 3.5.1 From Bulk to Ultrathin Nanostructures

**Figure 19:**
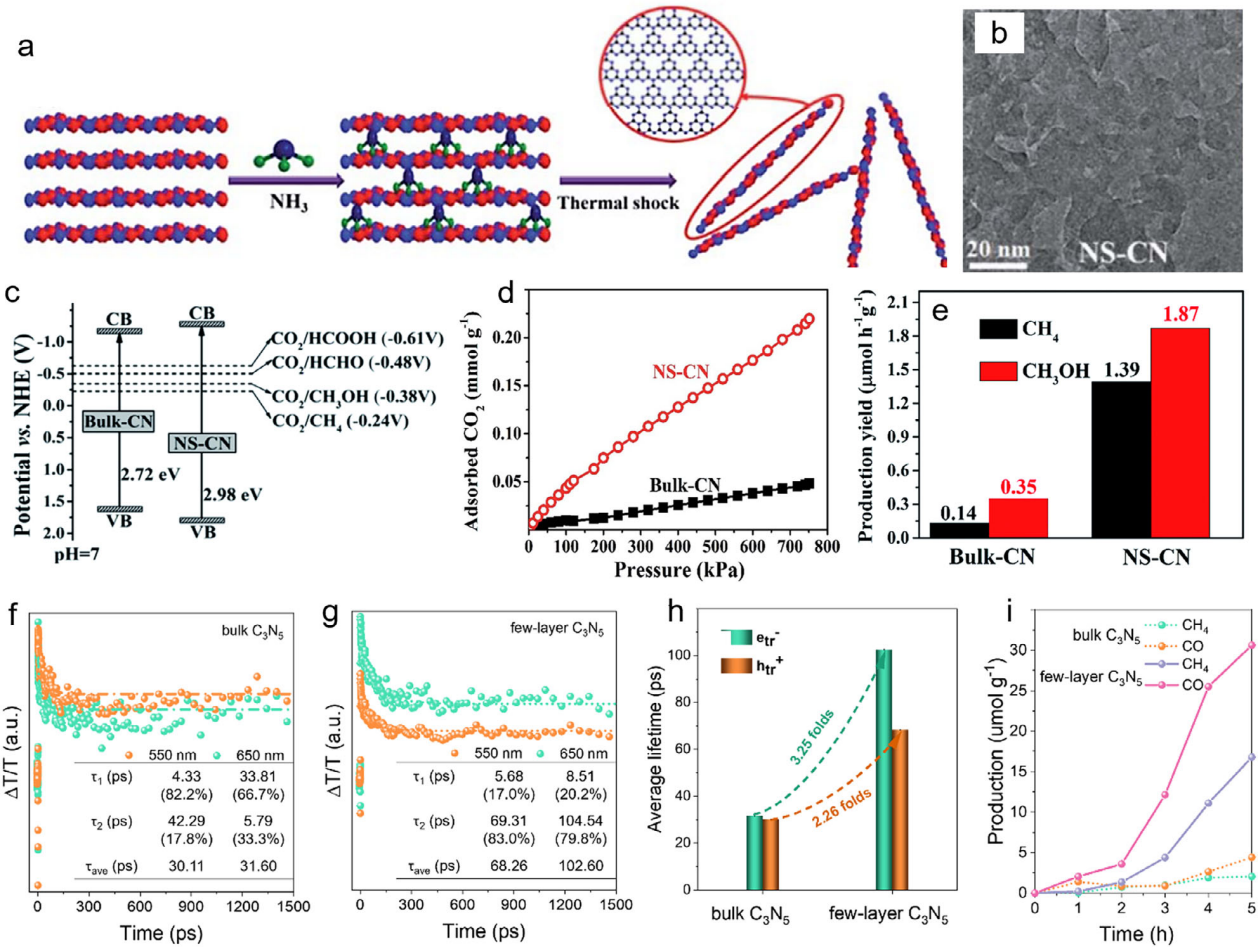
a) Synthesis of the nanosheets via a thermal shock method. b) TEM image of the NS‐CN nanosheets c) Band structure diagram for driving CO_2_ reduction process. d) CO_2_ adsorption measurement. e) Photocatalytic CO_2_‐to‐CO conversion over the bulk and thin carbon nitrides.^[^ ^98^ ^]^ Copyright 2017 The Royal Society of Chemistry. f,g) Transient absorption indicated distinct differences in the electron and hole decay dynamics at 550 and 650 nm between bulk and few‐layer C_3_N_5_. h) Comparisons of the average lifetimes of trapped carriers in bulk and thin C_3_N_5_. i) Photocatalytic CO and CH_4_ generation over the C_3_N_5_ polymers.^[^ ^21^ ^]^ Copyright 2022 American Chemical Society.

The dimensional reduction from bulk to nanosheets morphology enables enhanced light absorption with a broader bandgap, larger specific surface area for high CO_2_ adsorption, and improved charge transport within photocatalysts. For example, Xia et al. prepare ultrathin nanosheets (NS‐CN) from bulk carbon nitride (Bulk‐CN) via thermal shock method in NH_3_ gas (**Figure** **19**a,b).^[^ ^98^ ^]^ The XRD patterns of pristine Bulk‐CN and 2D NS‐CN nanosheets exhibit identical (100) and (002) peaks, indicating the main structures of g‐C_3_N_4_: planar tri‐s‐triazine and periodic stacking of the conjugated aromatic motifs. Notably, shown in Figure 19c, NS‐CN materials exhibits a broader bandgap with the more negative conduction band and more positive valence band than the bulk, suggesting enhanced redox capabilities of its photogenerated carriers. As shown in Figure 19d, NS‐CN shows an enhanced CO_2_ uptake over 4 times that of Bulk‐CN. This is mainly due to enhanced physisorption from its high surface area and porosity, and chemisorption on amine groups from carbon nitrides. N_2_ adsorption‐desorption confirmed increased porosity and surface area of the nanosheets. The ≈2 nm thickness of the catalysts revealed the shortens carrier paths, promoting PCO_2_RR and CH_4_/CH_3_OH production (Figure 19e). With a thickness of ≈1.2 nm, another example of morphology tunes from bulk to few layers C_3_N_5_‐based nanosheets have been constructed through liquid‐phase exfoliation processes.^[^ ^21^ ^]^ As reflected by the prolonged lifetimes of both e^–^ and h^+^ revealed in the transient absorption profiles, the morphological transition from bulk to few‐layer structures substantially enhances charge separation and transfer efficiency. Further, the decay kinetics of e^–^ and h^+^ in both bulk and few‐layer C_3_N_5_ were examined by fitting the transient absorption curves at 550 nm (trapped h^+^) and 650 nm (trapped e^–^) (Figure 19f,g). As summarized, the lifetimes of trapped e^–^ and h+ in few‐layer C_3_N_5_ were markedly prolonged compared to the bulk. Specifically, the average lifetime of e^–^ increases by 3.25 times, while that of h+ extends by 2.26 times (Figure 19h). In light of this enhanced light‐induced carrier kinetics, the main reduction products from few‐layer C_3_N_5_ are CO and CH_4_, with yields reaching 30.6 and 16.8 µmol g^−1^ after 5 h—both significantly higher than the bulk C_3_N_5_.

**Figure 20:**
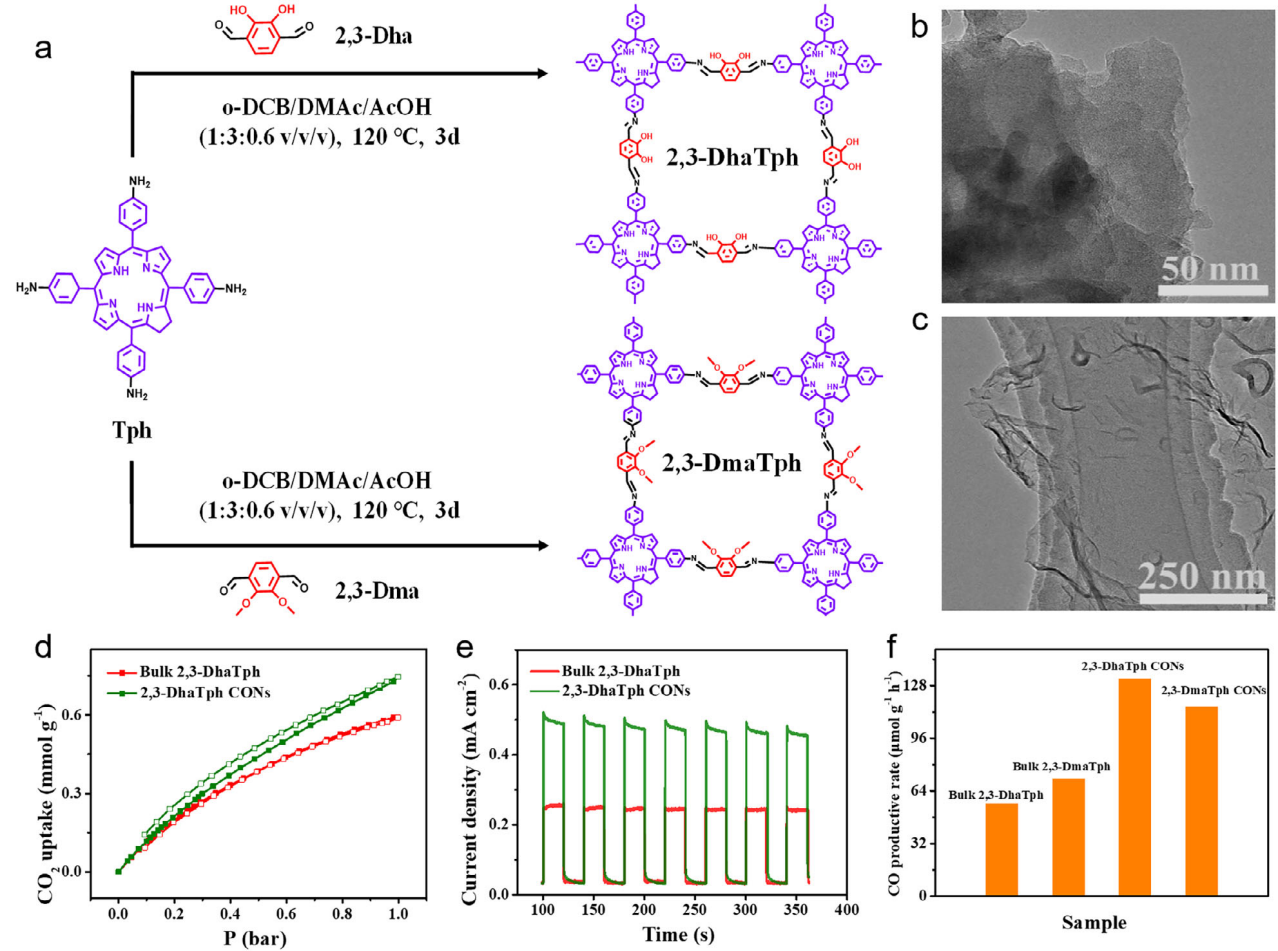
a) Preparation of 2,3‐DhaTph and 2,3‐DmaTph COFs. b,c) TEM images of the bulk and thin 2,3‐DhaTph COF materials. d) CO_2_ adsorption isotherms on both bulk and thin COFs. e) Photocurrents density‐time curves under light irradiation. f) The CO evolution rate over the 2,3‐DhaTph, 2,3‐DmaTph COFs, and 2,3‐DhaTph, 2,3‐DmaTph ultrathin nanosheets.^[^ ^99^ ^]^ Copyright 2022 The Royal Society of Chemistry.

To maximize the exfoliation yield of bulk COFs into 2D ultrathin CONs, the use of ionic liquids (ILs) to facilitate COF exfoliation has been studied (**Figure** **20**a).^[^ ^99^ ^]^ As shown in the TEM images (Figure 20b,c), the lateral size of the bulk 2,3‐DhaTph COF was ≈500 nm, while the exfoliated CONs exhibited few‐layer structures, mostly under 2.0 nm. The optical bandgap of CONs slightly increased after exfoliation (from 1.53 to 1.57 eV), indicating an improved light absorption capacity compared to the bulk COF. In addition to the enhanced CO_2_ adsorption capacity and broader bandgap derived from the intrinsic properties of the ultrathin samples, the superior PCO_2_RR performance of CONs is also attributed to their more accessible photocatalytic active sites, lower charge‐transfer resistance, and suppressed recombination of photogenerated electron–hole pairs (Figure 20d). Among all bulk samples studied, the ultrathin CONs demonstrated the highest photocurrent response during the incident‐photon‐to‐current conversion efficiency tests (Figure 20e). The ultrathin porphyrin‐based CONs showed excellent photocatalytic CO_2_‐to‐CO conversion under visible light in the gas‐solid phase, achieving a CO production rate of 132.2 µmol g^−1^ h^−1^ with nearly 100% selectivity—almost twice that of the bulk catalyst. These results confirm that the enhanced photocatalytic performance of the nanosheet‐based catalysts is primarily due to their improved CO_2_ adsorption capacity, more accessible active sites, lower charge‐transfer resistance, and longer exciton lifetimes—all of which contribute to catalysts’ superior PCO_2_RR performance.

#### 3.5.2 3D Reticular Design

**Figure 21:**
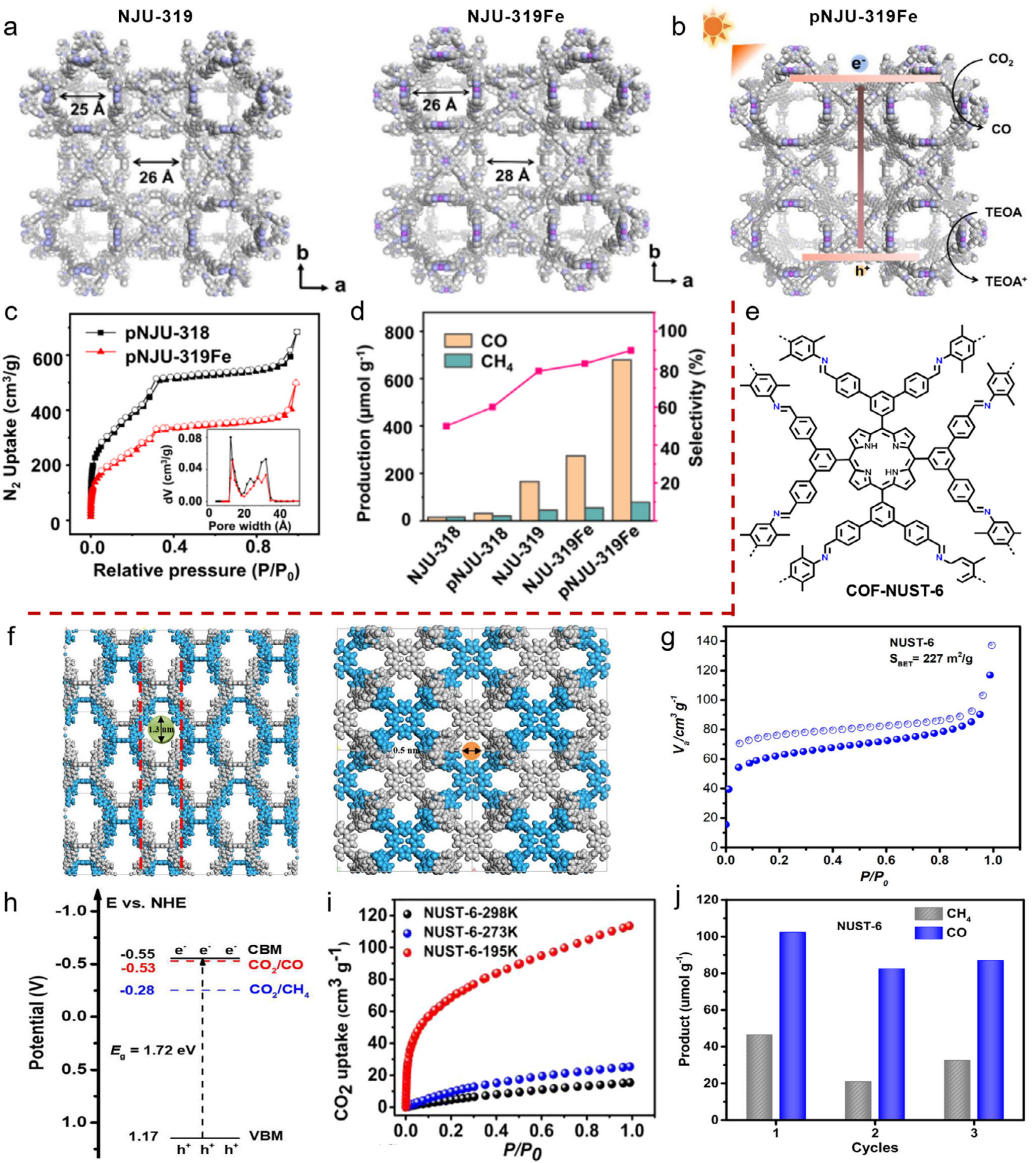
a) Structural models for NJU‐319 and NJU‐319Fe. b) Structural representations of pNJU‐319Fe and the associated PCO_2_RR mechanism. c) N_2_ sorption isotherm curve with the corresponding pore‐size distribution profile. d) CO_2_ photoreduction performances over the samples.^[^ ^100^ ^]^ Copyright 2023 American Chemical Society. e) Reticular framework of NUST‐6 with pcb topologies. f) Pore sizes in NUST‐6 catalysts. g) Porosity and specific surface area of the 3D COFs were evaluated by N_2_ sorption analysis at 77K. h) Porosity and specific surface area of the 3D COFs were evaluated by N_2_ sorption analysis at 77. i) CO_2_ sorption tests of NUST‐6. j) Photocatalytic generation of CO and CH_4_ over NUST‐6.^[^ ^101^ ^]^ Copyright 2022 American Chemical Society.

Compared to the layered stacked 2D morphology, 3D COFs feature interconnected pores and fully exposed functional groups, opening new opportunities for the post‐synthetic modification to obtain the advanced functional materials. Due to their open architecture and accessible active sites, the 3D COFs exhibit high surface area and tunable pore structures, which benefit both CO_2_ diffusion/adsorption and active site accessibility. For example, Lei recently reported a post‐synthetic cyclization strategy in 3D COFs to promote CO_2_ photoreduction ability. The resulting 3D frameworks were constructed by linking hexaphenyltriphenylene (HPTTP) units with porphyrin or iron‐porphyrin linkers or by further post‐synthetic cyclization strategy (NJU‐319, NJU‐319Fe, pNJU‐319Fe).^[^ ^100^ ^]^ In pNJU‐319Fe, the HPTTP units were then cyclized post‐synthetically to form π‐conjugated hexabenzocoronenes. Analysis from N_2_ sorption measurements confirmed the permanent porosity of pNJU‐319Fe with typical type‐IV isotherms. The BET surface areas for NJU‐319, NJU‐319Fe, and pNJU‐319Fe were 1511, 1354, and 951 m^2^ g^−1^, respectively. Pore size distributions revealed dual pore structures, corresponding to small cubic pores (15Å) and large tetragonal channels (30 Å), which matched well with simulated models. During PCO_2_RR test, CH_4_ and CO production reached 11.93 µmol·g^−1^·h^−1^ and 38.99 µmol·g^−1^·h^−1^, respectively (**Figure** **21**a–d). Compared with pristine NJU‐319Fe (26.8 µmol·g^−1^·h^−1^), the cyclized pNJU‐319Fe achieved a 2.5‐fold enhancement in CO production (68.8 µmol·g^−1^·h^−1^), owing to improved π‐conjugation for the enhanced light harvesting. In contrast, metal‐free NJU‐319 produced only 16.5 µmol·g^−1^·h^−1^ of CO, highlighting the catalytic role of Fe‐porphyrin centers. Thus, the 3D porous pNJU‐319Fe serves as an ideal photocatalyst, combining strong visible‐light absorption, active Fe–porphyrin sites, facile site exposure, and excellent mass transport in one material.

In another example, Zhang designed a cubic 3D porphyrin‐based COF (NUST‐6) using an 8‐connected node TTEP and dimethyl‐para‐phenylenediamine (Figure 21e).^[^ ^101^ ^]^ Nonlocal density functional theory (NLDFT) calculations revealed that the crystal NUST‐6 structure possessed the pores with the sizes of ∼5.0 and ∼13.0 Å. NUST‐6 displayed a sharp PXRD pattern, indicating an isoreticular pcb topology (Figure 21f). The type‐I isotherm, performed N_2_ sorption analysis at 77 K, confirmed its microporous nature with a surface area of 227 m^2^ g^−1^ (Figure 21g). For photocatalysis, NUST‐6 demonstrated strong visible‐light absorption with an optical band gap of 1.75 eV. Its conduction band is more negative than CO_2_/CO and CO_2_/CH_4_ redox potentials, indicating thermodynamic favourability for PCO_2_RR. Sorption measurements at 195, 273, and 298 K further confirmed its CO_2_ capture capability, with uptake values of 119.21, 25.40, and 15.42 cm^3^·g^−1^, respectively. For CO_2_‐to‐CO conversion, the metal‐free NUST‐6 catalysts achieved a CO production rate of 7.62 µmol·g^−1^·h^−1^, along with the CH_4_ production rate of 1.28 µmol·g^−1^·h^−1^. Recycle tests over 3 runs demonstrated its excellent chemical stability (Figure 21h–j). These findings underscore the potential of 3D COFs as efficient photocatalysts for solar‐to‐fuel conversion.

### 3.6 Linkage Design

Promoting the polymeric photocatalysts’ activity of light‐driven CO_2_RR requires a multifaceted approach that includes improving their dispersibility, hydrophilicity, charge separation efficiency, and selectivity for CO_2_ conversion over competing reactions such as hydrogen evolution. These enhancements can be achieved through chemical modifications, local skeleton design, and incorporation of functional groups. This section focusing on linkage design explores key methodologies to enhance the efficiency of the catalysts toward PCO_2_RR.

#### 3.6.1 Enhancing Dispersibility/Hydrophilicity

**Figure 22:**
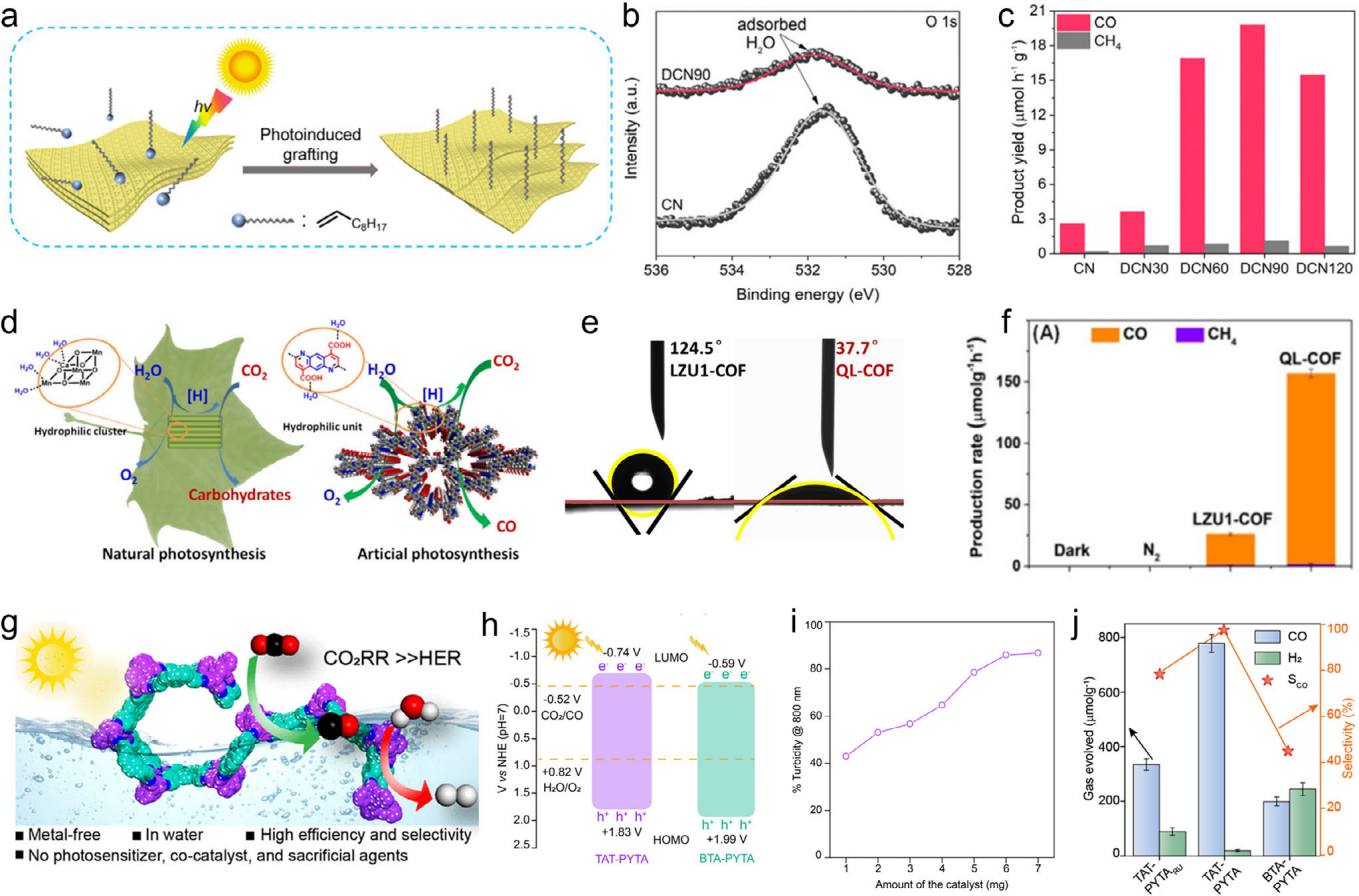
a) Synthesis of alkyl group‐functionalized g‐C_3_N_4_ with controlled hydrophilicity. b) High‐resolution XPS over the samples. c) Performance of alkyl group‐grafted g‐C_3_N_4_ (DCNT, T is the irradiation time).^[^ ^102^ ^]^ Copyright 2022 The Royal Society of Chemistry. d) Artificial photosynthesis using QL‐COF. e) H_2_O contact angles analysis over LZU1‐COF and QL‐COF. f) CO_2_ conversion performance.^[^ ^103^ ^]^ Copyright 2023 The Royal Society of Chemistry.

PCO_2_RR process usually takes place in aqueous or water–organic liquids, improving contact between the catalyst and the water phase is essential. Enhancing hydrophilicity allows the polymeric materials to disperse better, not only exposing more active surface area but facilitating effective interaction between CO_2_/H_2_O and the catalytic sites. Therefore, engineering the organic substrate with enhanced hydrophilicity will help increase the liquid–solid contact area, enabling more stable interfacial interactions and faster kinetics. For polymeric materials like CNs, CTFs, and COFs, they are generally hydrophobic and tend to aggregate in liquids. Therefore, improving catalysts’ dispersibility and hydrophilicity is vital for enhancing their PCO2RR performance. One effective method involves the functionalization of g‐C_3_N_4_ with alkyl groups, allowing for the control of hydrophilicity. **Figure** **22**a illustrates the modification of g‐C_3_N_4_ with the enhanced interaction with H_2_O molecules. The high‐resolution XPS O 1s spectra of CN and DCN90, shown in Figure 22b, demonstrate the differences in surface status between the untreated and alkyl‐functionalized g‐C_3_N_4_. This modification significantly boosts the photocatalytic CO_2_ reduction performance, as evidenced by the comparative chart over various irradiation times (Figure 22c).^[^ ^102^ ^]^ Another example involves the synthesis of hydrophilic COFs materials, such as the 4‐carboxyl‐quinoline COF (QL‐COF). These materials mimic artificial photosynthesis processes and exhibit improved H_2_O contact angles, indicating enhanced hydrophilicity (Figure 22d,e). The obtained QL‐COF demonstrated nearly 100% efficiency in PCO_2_RR to CO using pure water, showcasing its superior performance compared to other materials (Figure 22f).^[^ ^103^ ^]^

#### 3.6.2 Increasing Conductivity

**Figure 23:**
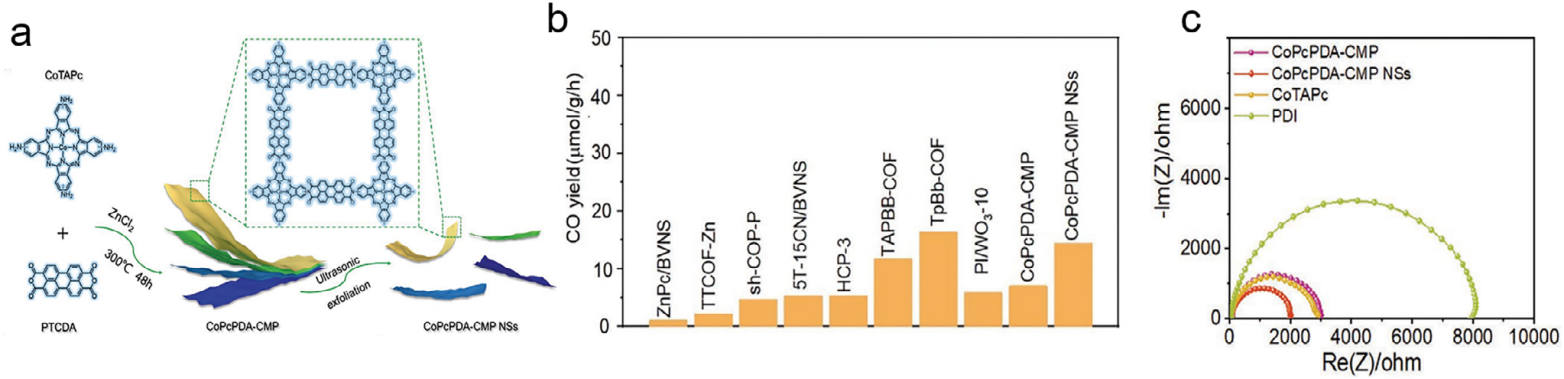
a) Synthesis of CoPcPDA‐CMP NSs. b) Comparison of the activities in CO_2_ reduction with H_2_O as electron donor. c) EIS of CoTAPc, PDI, CoPcPDA‐CMP, and CoPcPDA‐CMP NSs.^[^ ^104^ ^]^ Copyright 2022 Wiley‐VCH GmbH.

Increasing the conductivity of organic photocatalysts is essential to enhance their photocatalytic efficiency that facilitates the efficient transport of photogenerated carriers, thereby reducing recombination losses. As illustrated in **Figure** **23**a, the effective strategy is to synthesize CoPcPDA‐CMP NSs through a controlled polymerization process. When water is used as the electron donor during the catalysis, these NSs exhibited superior performance compared to previously reported organic photocatalysts (Figure 23b). The electrochemical impedance spectroscopy (EIS) data presented in Figure 23c confirm the enhanced conductivity of CoPcPDA‐CMP NSs, which exhibited lower resistance compared to CoTAPc and PDI catalysts. This reduced resistance indicates more efficient charge transport within the material, which is crucial for sustaining higher rates of CO_2_ reduction in pure water system.^[^ ^104^ ^]^

#### 3.6.3 Modifying Carrier Kinetics

**Figure 24:**
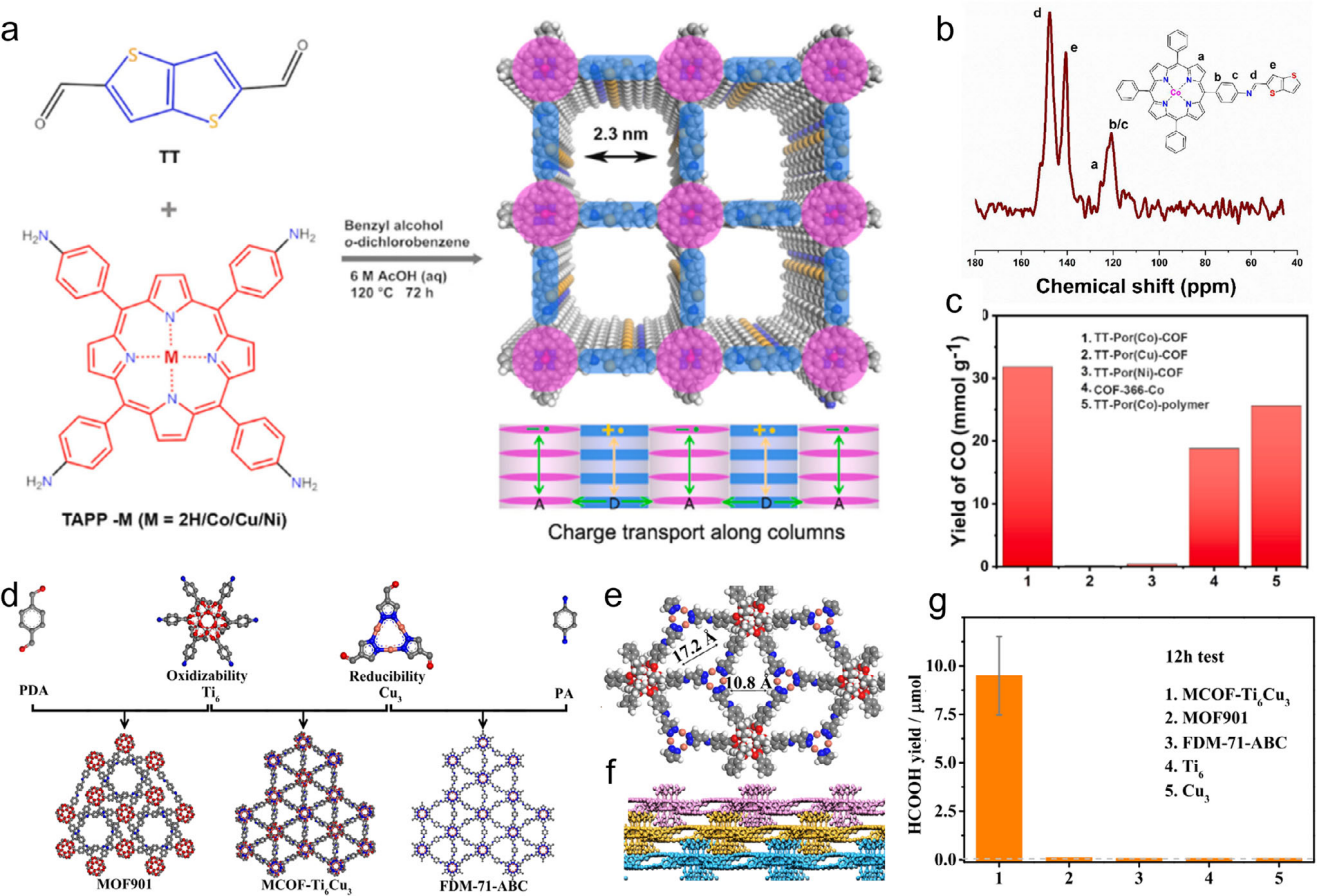
a) Schematic synthesis of TT‐Por(M)‐COF via the condensation of TT and TAPP‐M (M = 2 H/Co/Cu/Ni). b) ^13^C NMR spectra of TT‐Por(Co)‐COF. c) Photocatalytic performance.^[^ ^105^ ^]^ Copyright 2023 Elsevier B.V. d) Schematic illustration of the synthesis of MOF901, FDM‐71‐ABC, and MCOF‐Ti_6_Cu_3_. Top e) and side f) views of the structure of MCOF‐Ti_6_Cu_3_. g) Comparison of HCOOH yields.^[^ ^106^ ^]^ Copyright 2022, The Author(s).

Modifying carrier separation and transport within polymeric photocatalysts is a critical step to further enhance their overall CO_2_‐to‐fuels efficiency. Effective modification of carrier kinetics ensures that the photogenerated carriers can be efficiently separated and then fast transported to the reactive sites to maximize reaction activity. One successful strategy involves the synthesis of TT‐Por(M)‐COF via the condensation of TT and TAPP‐M (M = 2 H/Co/Cu/Ni) (**Figure** **24**a). The ^13^C NMR spectra of TT‐Por(Co)‐COF, as shown in Figure 24b, confirm the structural integrity of the synthesized COFs. Additionally, the incorporation of different metal centers significantly influences photocatalytic performance (Figure 24c). Comparative studies of these COFs reveal varying degrees of CO_2_ reduction efficiency, underscoring the importance of optimizing carrier separation to enhance photocatalytic activity.^[^ ^105^ ^]^ The synthesis of MOF901, FDM‐71‐ABC, and MCOF‐Ti_6_Cu_3_ represents another approach for modifying carrier kinetics. Figure 24d provides a schematic illustration of the synthesis process, while Figure 24e,f offers detailed structural views of MCOF‐Ti_6_Cu_3_. These structural modifications are crucial to facilitate the migration of charge carriers within the photocatalyst. The comparison of HCOOH yields for different samples, shown in Figure 24g, further highlights the impact of these modifications on the efficiency of CO_2_RR.^[^ ^106^ ^]^

**Figure 25:**
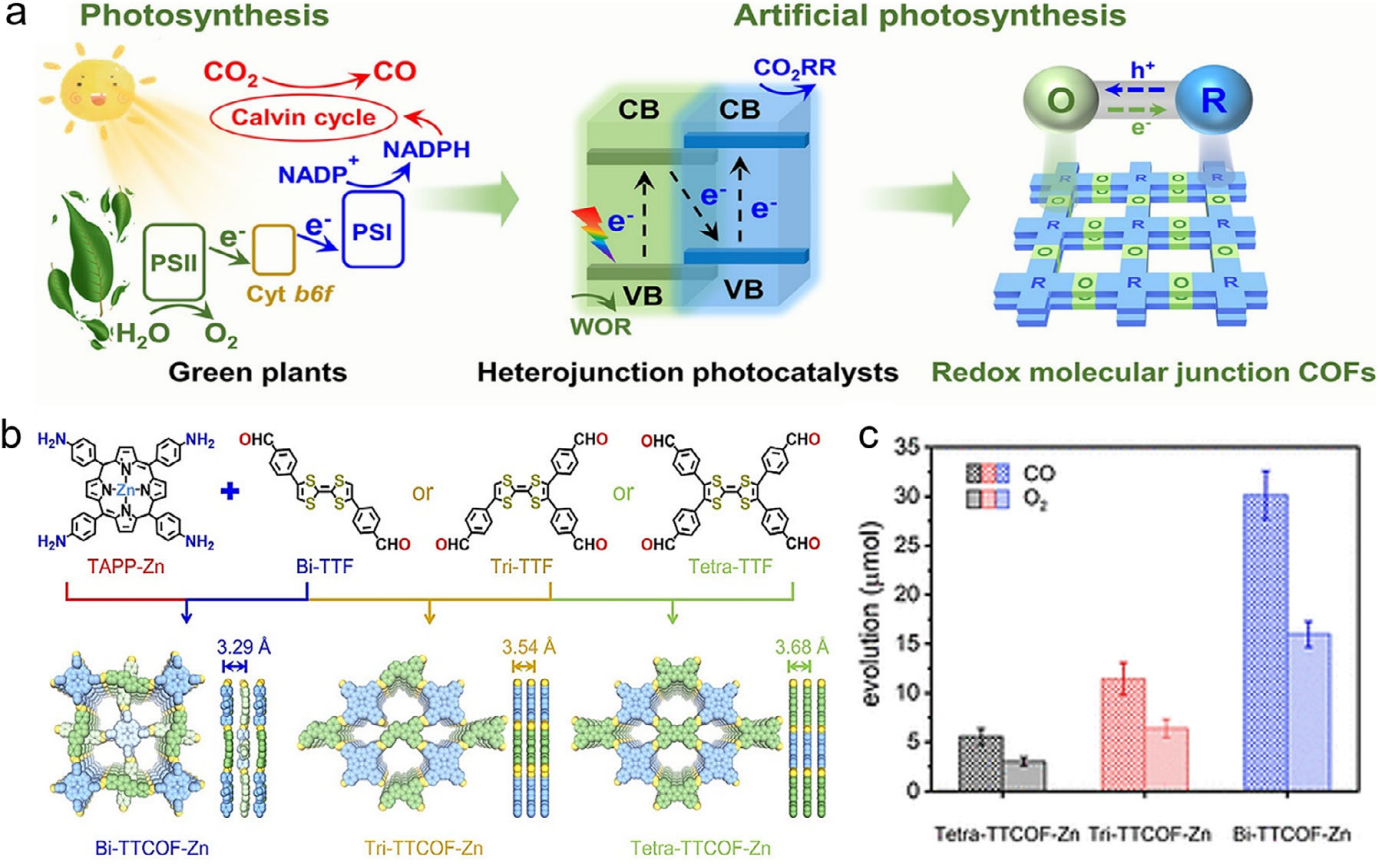
a) Schematic modes of redox units’ induction within COFs‐inspired artificial photosynthesis for the overall reaction. b) Structural description of M‐TTCOF‐Zn (M = Bi, Tri, or Tetra) materials. c) Reaction activity over the different COFs samples.^[^ ^107^ ^]^ Copyright 2023 American Chemical Society.

Coupling redox‐active molecular units into the framework is an effective strategy for facilitating photocatalytic reactions. In artificial photosynthetic systems, these redox‐active units mimic the natural process of photosynthesis by enabling the fast migration of electrons and protons during the photocatalysis. This approach has been reported to increase the overall photocatalytic performance of these polymer‐based systems, especially for CO_2_RR. In a recent study, COFs with different redox‐active molecular junction were synthesized, and their structural features were tailored to promote effective charge transfer during PCO_2_RR.^[^ ^107^ ^]^ The COFs integrated metal‐based molecular junctions such as TAPP‐Zn, Bi‐TTF, Tri‐TTF, and Tetra‐TTF, leading to a well‐defined arrangement of redox units (**Figure** **25**a,b). The distances between the molecular junctions, ranging from 3.29 Å to 3.68 Å, facilitated efficient electron transfer, which is critical for overall reaction kinetics. As shown in Figure 25c, the photocatalytic activity of these COFs was measured by monitoring the evolution of CO and O_2_ products. Among the different COFs studied, the Tetra‐TTFCOF‐Zn exhibited the highest CO and O_2_ production rates, showcasing the importance of redox unit placement in optimizing the performance of polymer‐based photocatalysts. These findings suggest that the incorporation of redox‐active units into the frameworks can significantly enhance the efficiency of solar‐driven CO_2_ reduction, aligning with the broader goals of artificial photosynthesis and sustainable chemical production.

**Figure 26:**
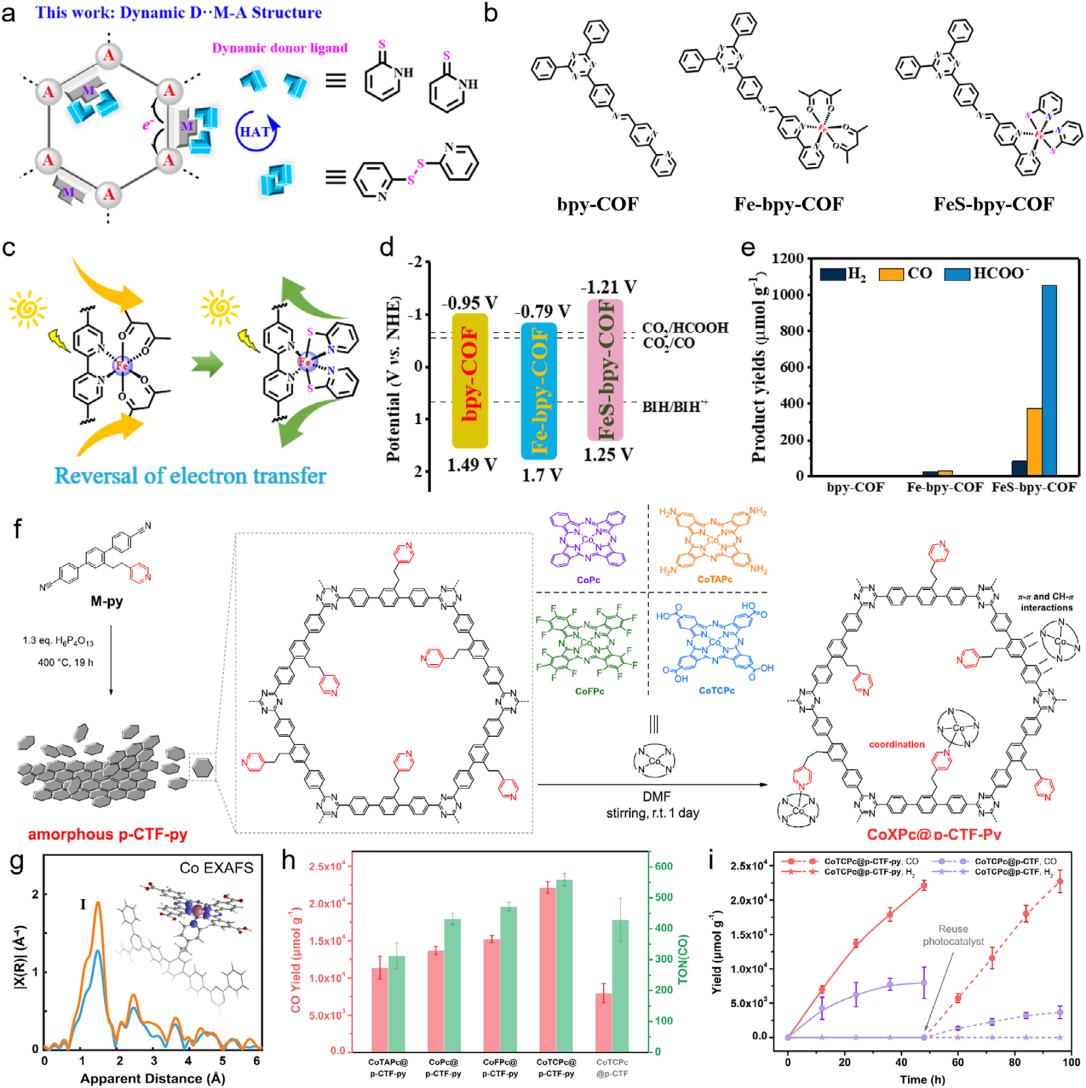
a) Diagram of dynamic D‐M‐A polymers. b) Local molecular‐level structural information from bpy‐COF, Fe‐bpy‐COF, and FeS‐bpy‐COF. c) Electron transfer in Fe‐bpy‐COF reverses after pySH ligand coordination during photocatalysis. d) Band‐edge structure diagrams for facilitating CO_2_. e) Product yields after 4 h of light irradiation.^[^ ^108^ ^]^ Copyright 2025 the Royal Society of Chemistry. f) Synthesis of the pCTF‐py and CoXPc@p‐CTF‐py. g) EXAFS spectra for CoTCPc (blue) and CoTCPc@p‐CTF‐py (orange) samples; inset shows CoTCPc@p‐CTF‐py model. h) Comparison of PCO_2_RR performance among the hybrid photocatalysts. i) Long‐term photocatalytic activity profiles for CO and H_2_ generation.^[^ ^109^ ^]^ Copyright 2024, The Author(s).

To fully elaborate on the emerging role of dynamic coordination in improving charge carrier kinetics, more representative systems were considered in this section, both schematically and spectroscopically summarized. In the first system, pyridinethiol ligands were post‐synthetically introduced into Fe‐bpy‐based frameworks to construct dynamic D–M–A (donor–metal–acceptor) architectures (**Figure** **26**a,b). This reversible ligand coordination reshapes the local electronic structure under light irradiation, resulting in a directional reversal of electron flow from the ligand to the metal (Figure 26c). This behavior facilitates enhanced charge separation and suppresses undesired back electron transfer. As evidenced by product yields, band‐edge shifts induced by ligand exchange optimize redox potentials to promote selective CO_2_‐to‐HCOO^−^ conversion while minimizing H_2_ evolution, (Figure 26d,e).^[^ ^108^ ^]^ The second example involves the immobilization of cobalt phthalocyanine (CoTCPc) complexes onto a porous triazine framework via coordination with pyridyl‐functionalized backbones. A structural model of the resulting CoTCPc@p‐CTF‐py shows homogenous distribution of metal sites within the polymeric matrix (Figure 26f). Analysis derived from the Fourier‐transformed EXAFS spectra further confirmed Co–N coordination interactions, which was distinct from the pristine CoTCPc (Figure 26g), indicating strong interfacial bonding. This architecture enables directed charge flow, leading to superior turnover frequencies (Figure 26h) and stable CO evolution (Figure 26i).^[^ ^109^ ^]^ These examples highlight complementary approaches to modulating carrier behavior: through either dynamic ligand shuttling or stable coordination‐induced rectification. These strategies open new directions for precise control over charge migration and long‐lived excited states in heterogeneous PCO_2_RR systems.

#### 3.6.4 Activating Broad‐Spectrum Capture

**Figure 27:**
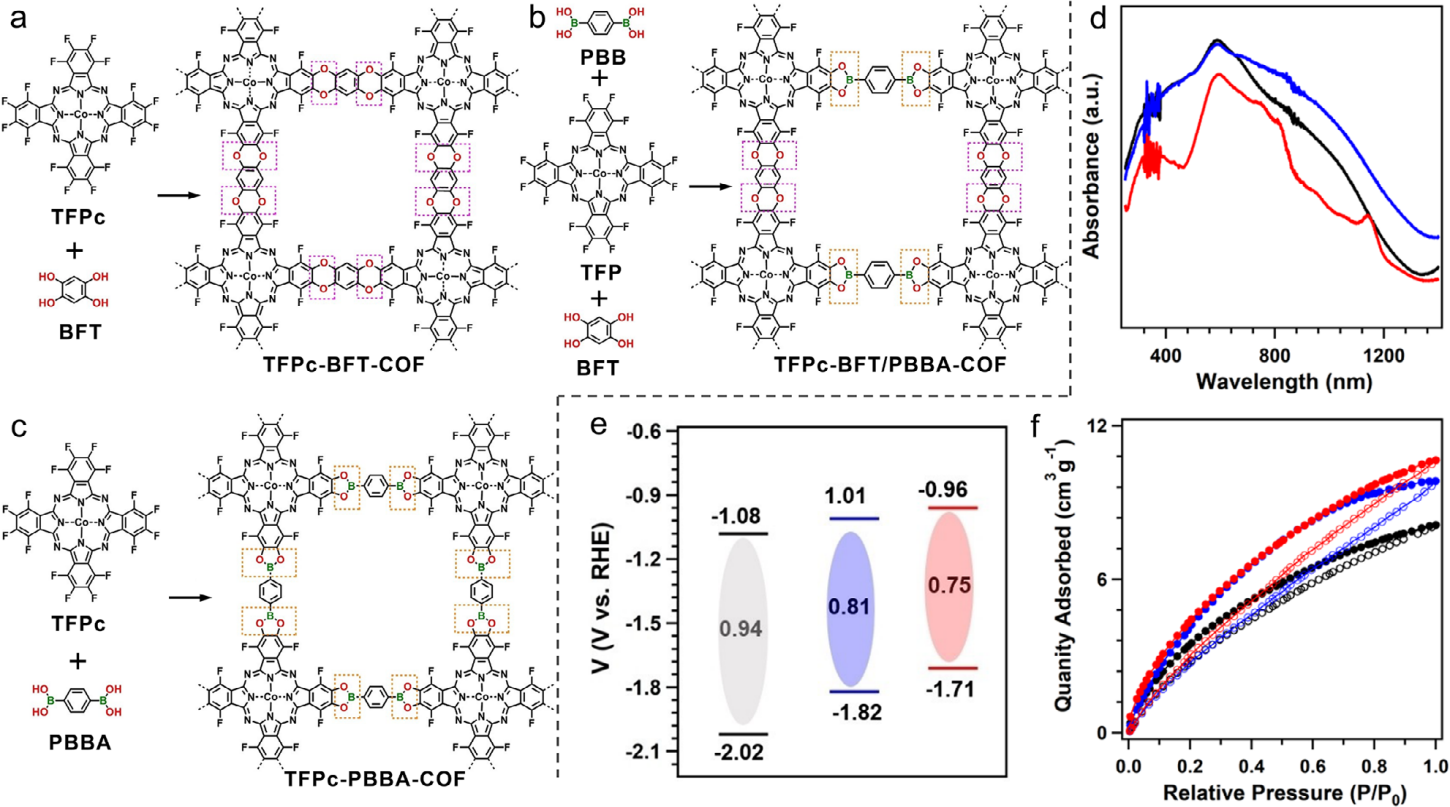
a–c) Description of the construction of TFPc‐BFT‐COF, TFPc‐BFT/PBBA‐COF, and c TFPc‐PBBA‐COF materials. d) UV–Vis spectra, e) Band structure diagram and f) CO_2_ sorption over the samples of TFPc‐BFT‐COF (black), TFPc‐BFT/PBBA‐COF (blue), and TFPc‐PBBA‐COF (red).^[^ ^114^ ^]^ Copyright 2023 Wiley‐VCH GmbH.

Over the past few decades, numerous strategies have been developed to achieve multielectron‐driven CO_2_ reduction under broad‐spectrum light.^[^ ^110^, ^111^ ^]^ However, realizing optimal performance remains a major challenge. Option 1 is to integrate hybrid composites on the organic photocatalysts with the materials that can extend light absorption, such as graphene, black phosphorus, and metal‐based nanoparticles. These materials have demonstrated excellent optical properties—graphene and black phosphorus can absorb a broader range of visible and even near‐infrared light, compensating for the spectral limitations of traditional semiconductors.^[^ ^112^ ^]^ Metal‐based nanoparticles is useful to obtain the plasmonics that exhibit strong localized surface plasmon resonance effects. This design not only enable the generation intense local electromagnetic fields that enhance light absorption but also result hot carriers that further facilitate charge separation and transfer.^[^ ^85^ ^]^ Option 2 is to tailor the chemical structure of the framework, the electronic structure of the catalyst can be fine‐tuned to enhance light capture across a broad spectrum. For example, constructing COFs with low‐bandgap structures that enable carrier excitation under visible and near‐infrared (NIR) light. Incorporating photoactive units such as triazine and porphyrin has shown great potential in this regard. Wang developed a series of COFs with triazine‐imide‐triazine motifs and found that reducing the number of triazine units narrowed the bandgap.^[^ ^55^ ^]^ As estimated by the Kubelka–Munk function, the resulting 2N‐COF, 1N‐COF, and 0N‐COF membranes exhibited absorption edges at 525, 552, and 618 nm, respectively, with corresponding optical bandgaps of 2.44, 2.31, and 2.09 eV. Materials with narrower bandgaps can absorb longer wavelengths which is more easily excited by low‐energy photons, thus increasing the number of photo‐generated carriers. For another example, Jiang et al. constructed COFs using porphyrin and phthalocyanine units. These COFs were synthesized using topological strategies like [C_4_ + C_4_] and [C_4_ + C_2_], yielding microporous and mesoporous materials. Two‐photon absorption (TPA) monomers integrated into the skeletons were essential for converting low‐energy NIR photons into high‐energy visible photons for chemical reactions.^[^ ^113^ ^]^ Recently, Zeng et al. synthesized the COFs with varying boron (B) content using a nucleophilic substitution method. As shown, TFPc‐BFT‐COF was created with Co‐hexadecafluorophthalocyanine (TFPc) as the node and benzene‐1,2,4,5‐tetraol (BFT) as the linker (**Figure** **27**a–c). A mixed‐linkage COF, TFPc‐BFT/PBBA‐COF, was obtained by combining TFPc nodes with both BFT and 1,4‐phenylenediboronic acid (PBBA) linkers. Lastly, TFPc‐PBBA‐COF, constructed using TFPc and PBBA exclusively, exhibited strong light absorption across a broad range (250–1500 nm, Figure 27d). Correspondingly, the highest occupied molecular orbital (HOMO) position of TFPc‐PBBA‐COF was recorded at –1.71 V (vs. RHE), higher than TFPc‐BFT/PBBA‐COF (–1.82 V) and TFPc‐BFT‐COF (–2.02 V), reflecting superior reductive properties (Figure 27e). Considering the importance of CO_2_ sorption for PCO_2_RR catalysts, CO_2_ sorption capacity was evaluated at 273 K. TFPc‐PBBA‐COF displayed the highest CO_2_ uptake at 18.6 m^2^ g^−1^, compared to TFPc‐BFT‐COF's 15.2 m^2^ g^−1^ and TFPc‐BFT/PBBA‐COF's 16.7 m^2^ g^−1^ (Figure 27f). Additionally, TFPc‐PBBA‐COF also showed a larger exchange loop, likely due to stronger CO_2_ interactions. The results indicate that the B atoms in the linkages significantly affect electronic conductivity and CO_2_ binding strength. It should be emphasized that although the polymeric COFs introduced in this work are suitable for PCO_2_RR, the experiments were only used to evaluate electrocatalytic CO_2_ reduction.^[^ ^114^ ^]^ So far, the photocatalysis under near‐infrared light region, especially for PCO_2_RR, remains inadequate, making it a recent focus of research in this field.

#### 3.6.5 Branched Skeleton via Covalent Grafting Strategies

**Figure 28:**
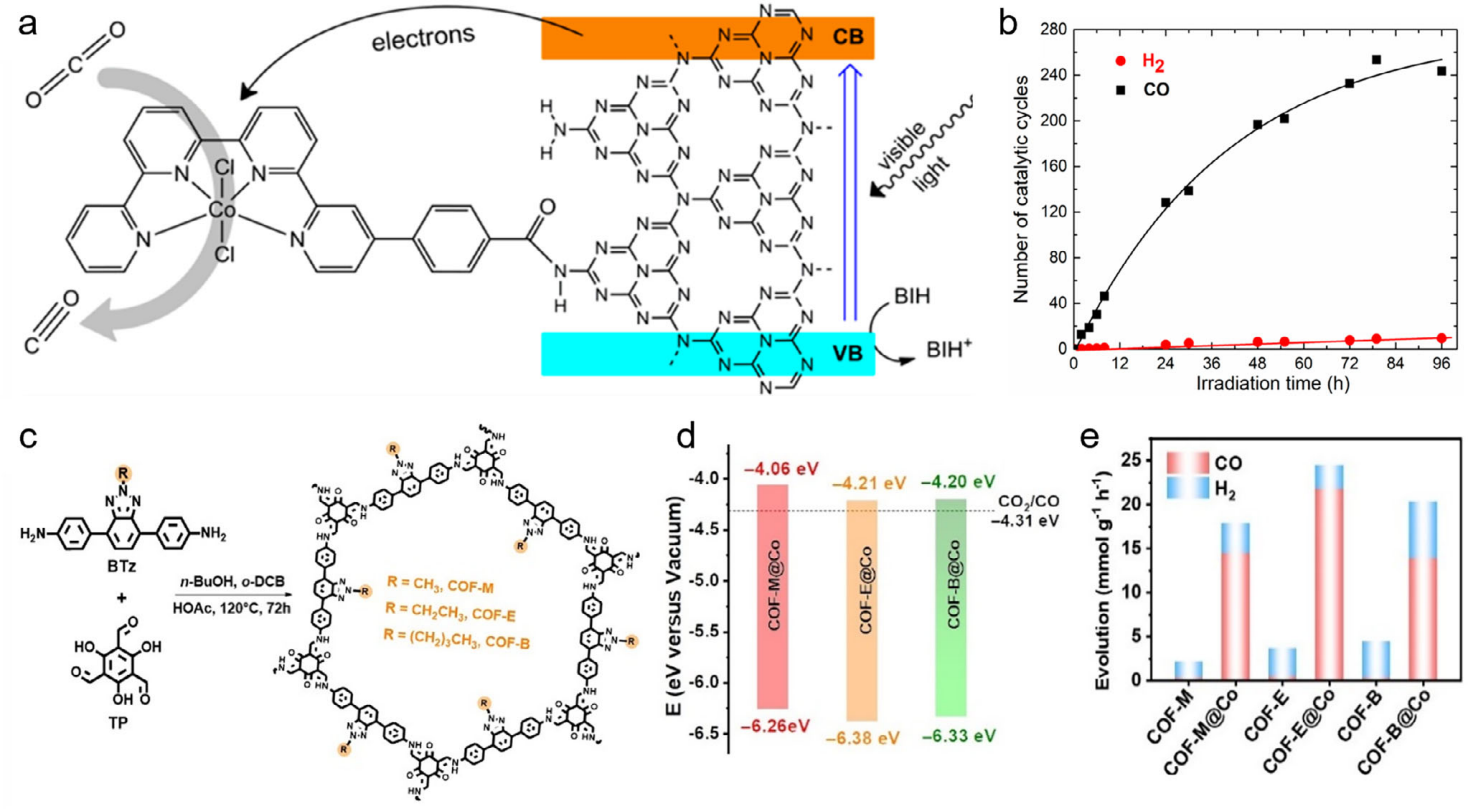
a) Schematic of CO_2_ conversion over Coqpy@mpg‐C_3_N_4_. b) PCO_2_RR test under a CO_2_‐saturated ACN solution.^[^ ^115^ ^]^ Copyright 2020 American Chemical Society. c) Synthetic route of three types of COFs tailored by side chains. d) Band alignment over the samples. e) Performance comparison over the Co loaded COFs catalysts.^[^ ^116^ ^]^ Copyright 2025 Wiley‐VCH GmbH.

Unlike the commonly pursued modifications of the COF backbone, tailoring side functionalities enables precise tuning of the electronic environment, surface polarity, and catalytic microenvironment, thereby enhancing charge transfer dynamics and CO_2_ activation. Herein, Covalent grafting of organic polymer semiconductors via side‐chain engineering represents a powerful yet underexplored strategy to optimize PCO_2_RR. In a representative study, a cobalt quaterpyridine complex bearing a carboxylic acid anchoring group was covalently linked to the surface of mesoporous carbon nitride (mpg‐C_3_N_4_) through amide bond formation via EDC/HOBt coupling chemistry.^[^ ^115^ ^]^ As illustrated in the **Figure** **28**a, this approach enables strong immobilization of the Co‐based materials within mpg‐C_3_N_4_ matrix, ensuring efficient electron flow from the photoexcited C_3_N_4_ to the metal Co centers. The hybrid catalyst exhibited excellent photocatalytic CO_2_‐to‐CO conversion under visible light irradiation, with high turnover cycles and minimal H_2_ production (Figure 28b). This work exemplifies how covalent binding between light‐harvesting semiconductors and catalytic sites can not only create stable configuration but facilitate directional charge migration for PCO_2_RR. Recently, Li reported that alkyl side chains with different lengths can be grafted to benzo[d,1,2,3]triazole‒based β‒ketoenamine COFs.^[^ ^116^ ^]^ It is found that alkyl side chains can alter the properties of the as‒synthesized COFs (COF‐M, COF‐E and COF‐B), including interlayer stacking, crystallinity, specific surface area, light harvesting and charge transfer behavior (Figure 28c). The conduction bands of COF‐M@Co, COF‐E@Co, and COF‐B@Co were estimated to be −0.78, −0.63, and −0.64 eV vs NHE at a solution pH of 6.8. Based on their optical bandgaps, the analysis of band alignments confirm their thermodynamic capability to realize CO_2_‐to‐CO process (Figure 28d). Besides, after loading Co ions, all these materials exhibit significantly enhanced activity compared to their metal‐free counterparts. Being the most efficient catalyst in this study, COF‐E@Co achieves a CO production rate of 21.74 mmol g^−1^ h^−1^ and an apparent quantum yield of 13.3%, ranking among the highest reported for COFs‐based systems (Figure 28e).

## 4 Challenges and Future Perspectives for Organic Polymeric Materials

### 4.1 Ongoing Challenges to Enhancing Photoactive Organic Materials for CO_2_ Reduction

For solving the ongoing challenges to enhancing photoactive organic materials for CO_2_ reduction, the achievement of effective light absorption across the solar spectrum, especially in the visible and NIR regions, is crucial for achieving efficient photocatalysis. Thus, advanced material engineering, including band gap modulation and heteroatom doping, enhances light capture. Low solar‐to‐chemical conversion efficiencies, usually caused by poor charge mobility, suboptimal band gaps, and particularly complex and sluggish reaction kinetics during PCO_2_RR, remain a significant challenge. Therefore, developing efficient PCO_2_RR catalysts faces numerous key challenges due to the intrinsic complexity of the photochemical CO_2_ conversion process, requiring simultaneously addressing light capture, charge generation and separation, CO_2_ adsorption affinity, multi‐electron transfer steps, and the integration of catalytic sites. Among these factors, the energy coupling between CO_2_ adsorption, activation, and charge transfer is particularly vital for overcoming thermodynamic and kinetic barriers in PCO_2_RR. In this regard, evaluating the isosteric heat of adsorption (Q_st_) is crucial, as it reflects a photocatalyst's thermodynamic ability to adsorb and activate CO_2_ molecules and intermediates.

Accordingly, we compiled Q_st_ values of representative CNs, CTFs, and COFs photocatalysts from the literature for comparative analysis (**Table** **3**).^[^ ^79^, ^101^, ^117^, ^118^, ^119^, ^120^ ^]^ It is known that the stable formation of CO_2_^−^ intermediates on the catalyst surface relies on strong CO_2_ adsorption, efficient electron donation from the photocatalyst, and rapid charge transfer to generate stable adsorbed anionic species. Appropriate strong adsorption facilitates both electron injection and intermediate stabilization. For example, the constructed 1D/2D polyheptazineimide‐based crystalline CN/graphene (CNNA/rGO) heterojunctions can strongly adsorbs CO_2_ molecules.^[^ ^121^ ^]^ With the modulated environment, the fast charge transfer behavior from rGO to CNNA were induced by charge redistribution and enhanced polarity and polarizability, which were beneficial for increasing CO_2_ adsorption strength. Using the Clausius‐Clapeyron equation to calculate the isosteric heat of CO_2_ adsorption, the 1D/2D heterojunctions not only exhibited a high CO_2_/N_2_ selectivity of 44, but also possessed a Q_st_ as high as 55.2 kJ mol^−1^, much greater than those of CNNA (15.7 kJ mol^−1^) and rGO (18.2 kJ mol^−1^). The CO_2_ adsorption capacity increases significantly with pressure, while N_2_ adsorption changes little, indicating strong CO_2_ adsorption and excellent selectivity. As the results, this material shows superior PCO_2_RR performance under pure water conditions, with production rates of CH_4_ at 4.30 µmol·g^−1^·h^−1^, CO at 6.65 µmol·g^−1^·h^−1^, CH_3_OH at 0.53 µmol·g^−1^·h^−1^, and C_2_H_5_OH at 1.15 µmol·g^−1^·h^−1^, resulting in a total CO_2_ conversion rate of 12.63 µmol·g^−1^·h^−1^. In this system, moderately strong adsorption heat can more effectively activate CO_2_ molecules, stabilize reaction intermediates, and facilitate multi‐electron transfer and C–C coupling, thereby promoting the formation of the multi‐carbon products. However, it is important to note that excessively strong adsorption is not always beneficial, as it may cause intermediate species to be trapped on the surface, hindering product release. In order to drive the reaction toward the desired products, it is necessary to develop catalysts with abundant active sites, optimized band structure, and tunable surface properties. Also, stability is another issue that needs to be solved as polymer‐based photocatalysts are inclined to degrade under light exposure, leading to loss of activity. To improve durability, strategies like self‐healing designs, protective coatings, and robust composite structures are under development.^[^ ^111^, ^113^, ^122^ ^]^

**Table 3:** Reported Q_st_ values of representative CNs, CTFs, and COFs photocatalysts from the literature for comparative analysis.

Catalysts	Textural features		O_st_ [kJ mol^−1^]	PCO_2_RR measurements		Ref.
	SSA [m^2^ g^−1^]	PV [cm^3^ g^−1^]		Product [µmol g^−1^ h^−1^]	Selectivity	
g‐C_3_N_4_	47.1	0.08	17.5	CO, 2.89	N.A.	[79]
Nv‐rich‐CN	293.1	0.43	41.8	CO, 6.61	N.A.	
CN	N.A.	N.A.	25.2	CO, 1.1	73.3%	[117]
Ni5‐CN	N.A.	N.A.	49.5	CO, 8.6	81.1%	
TpPa	779.62	57.47	33.61	CO, 95.17	N.A.	[118]
TpPa‐SO_3_H	63.61	34.28	34.8	CO, 104.15	N.A.	
PI‐COF‐TT	825	N.A.	29.76	CO, 483	93%	[119]
NUST‐5	680	N.A.	24.32	CO, 5.5	76%	[101]
NUST‐6	N.A.	N.A.	25.86	CO, 7.6	86%	
CTF‐TT	377	0.31	32.3	HCOOH, ∼30.6	N.A.	[120]
CTF‐TT‐Ir	111	0.16	18.9	HCOOH, 245	97%	

SSA – specific surface area, PV – pore volume, Q_st_ – isosteric heat of adsorption, N.A. – means not applicable.

### 4.2 Other Light‐Responsive Organic Materials for PCO_2_RR

**Figure 29:**
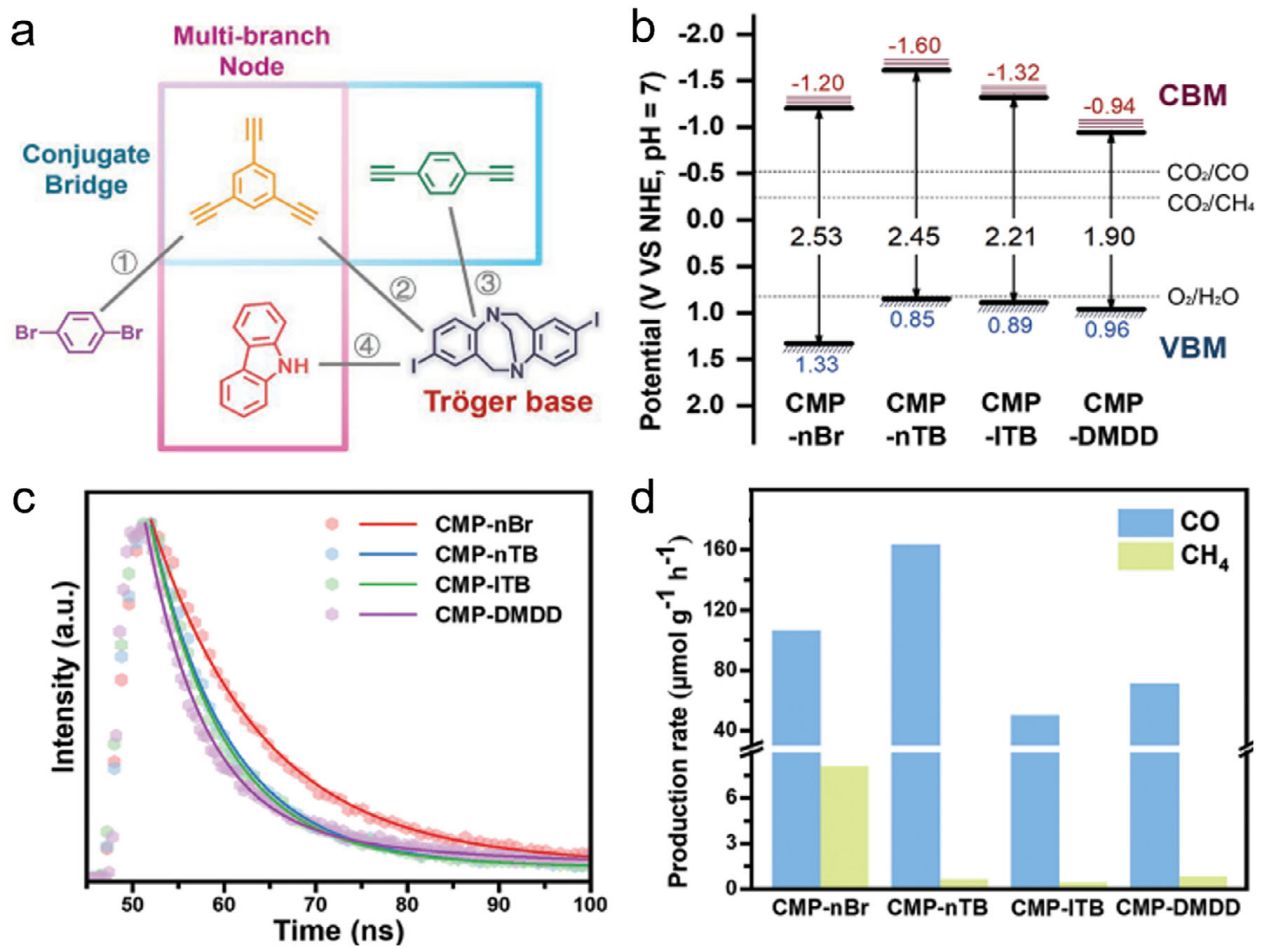
a) Diagram of the precursors for synthesizing Tröger's‐base‐derived conjugated microporous polymers. b) Band‐edge potential diagrams for facilitating light‐driven CO_2_ and H_2_O conversion. c) Time‐resolved photoluminescence spectroscopy. d) Evolution of CO and CH_4_ products.^[^ ^123^ ^]^ Copyright 2023 Wiley‐VCH GmbH.

To advance photocatalytic CO_2_ reduction systems aimed at carbon neutrality, recent studies have highlighted a new class of organic photocatalysts, conjugated microporous polymers (CMPs). For example, CMPs derived from Tröger's base have been reported to effectively facilitate overall CO_2_ and water conversion without the use of sacrificial agents.^[^ ^123^ ^]^ In this work, four CMP variants (CMP‐nBr, CMP‐nTB, CMP‐lTB, and CMP‐DMDD) were synthesized (**Figure** **29**a). Their optical properties and energy band diagrams confirm their ability to thermodynamically support the overall CO_2_ and H_2_O conversion (Figure 29b). This work pay attention to tailoring donor–acceptor conjugated structures to enhance O_2_ evolution and reduce competing reactions toward PCO_2_RR. Time‐resolved photoluminescence and free energy landscape studies have identified trap sites and energetically favorable oxygen evolution pathways (Figure 29c). Notably, CMP‐nTB catalysts demonstrated appropriate band‐edge alignment and robust charge separation, achieving high CO production rates (163.53 µmol g^−1^ h^−1^) under visible light (Figure 29d). In addition to conjugated microporous polymers (CMPs), several related conjugated organic catalysts are playing an increasing role in PCO_2_RR—such as linear conjugated polymers (LCPs), hypercrosslinked polymers (HCPs), covalent porphyrin polymers (COPs), porous organic polymers (POPs), and porous polymerized organic materials (POMs)—have recently emerged as promising organic photocatalysts.^[^ ^124^, ^125^, ^126^, ^127^, ^128^, ^129^, ^130^ ^]^ As such, more attention and prospects should be directed toward these organic semiconductors toward PCO_2_RR in the future (**Table** **4**).

**Table 4:** Reported conjugated materials for PCO_2_RR photosystems.

Catalyst	Types	System types temperature	Light source	Esa/Ps/Ca	Output [µmol g^−1^ h^−1^]	Selectivity	Refs.
TDO‐T	LCPs	Solid‐gas system N.A.	Xe lamp, 300W > 420 nm	no/no/no	CO 229.36	78.4%	[124]
TDO‐2T					CO 379.35	79.5%	
CLP‐CS	LCPs	Solid‐liquid system N.A.	Xe lamp, 300W	TEOA/no/[Co(bpy)_3_]^2+^	CO 1613	90%	[125]
HCP‐3	HCPs	Solid‐gas system Ambient temperature	Xe lamp, 400W UV–Vis	no/no/no	CO 15.6	78%	[126]
PEosinY‐1	COPs	Solid‐gas system 25 °C	Xe lamp, 300W > 420 nm	no/no/no	CO	33%	[127]
AQB	COPs	Solid‐gas system N.A.	Xe lamp, > 420 nm	no/no/no	CO	∼70%	[128]
TPA‐DPA PPK	POMs	Solid‐gas system Room temperature	Xe lamp, 100W AM 1.5G	no/no/no	CH_4_	152.65%	[129]
TAT‐PYTA	POPs	Solid‐gas system 22 °C	Xe lamp, 420W	no/no/no	CO 77.8	98%	[130]

Esa (electron sacrificial agent); Ps (photosensitizer); Ca (cocatalyst); N.A. (not applicable).

### 4.3 Excluding Carbon Contamination During PCO_2_RR

Understanding the sources of carbon contamination and eliminating potential pollution before conducting experiments are critical to ensuring that PCO_2_RR experiments are carried out with due care to obtain reliable data. Carbon pollution refers to the introduction of carbon by the reaction products from sources other than CO_2_, such as the degradation of the photocatalyst itself. In PCO_2_RR process, products (like CO, CH_4_, or other hydrocarbons) are carbon‐based, making it crucial to differentiate the carbon derived from the CO2 feedstock or not. In this review, we divide the carbon contamination problem into two categories according to the sources of carbon pollution: carbon pollution from the catalysts and carbon pollution from experimental operations. 1) Carbon contamination can arise either from the self‐photodecomposition of the catalysts or from residual pollutants on the photocatalyst surface after synthesis. COFs, PCNs, and CTFs are constructed from organic compounds containing rich carbon within their frameworks. This creates a higher risk of structural breakdown from their photodecomposition process during the catalysis, which might interfere with the accurate quantification of CO_2_ reduction products, leading to false‐positive results.^[^ ^131^, ^132^ ^]^ Carbon contamination on the surface of photocatalysts, originating from organic reagents such as solvents, reactants, and surfactants used during synthesis, can be removed by several methods. Cleaning the catalysts with ethanol/water and further treating the catalysts in a vacuum oven at a slightly higher temperature for long hours can eliminate residual alcohol solvents and other organic agents.^[^ ^133^ ^]^ 2) Details regarding carbon pollution from experimental operations can be analyzed from several aspects. During the performing the PCO_2_RR process, carbon contamination can be originated from organic reagents that presented in solutions, gases, and device's surfaces (such as gloves, lab coats, and reactors). this kind carbon contamination can be effectively eliminated through rigorous cleaning procedures.^[^ ^131^ ^]^ In the case of CNs, CTFs, and COFs, these carbon‐based photocatalysts are particularly susceptible to carbon contamination. Considering the factors above, it is essential to perform careful operations—such as blank/control experiments, isotopic labeling tests (e.g., using ^13^C‐labeled CO_2_), and long‐term monitoring of photocatalyst stability—when working with carbon‐based photocatalysts to prevent carbon contamination.

### 4.4 Developing Sacrificial Agent‐Free Photosystems for Practical Applications

The PCO_2_RR systems comprise various components like photocatalysts, additional photosensitizers (e.g., metal‐based complexes, Ag_2_CrO_4_, and polydopamine, etc.),^[^ ^134^, ^135^, ^136^ ^]^ co‐catalysts (usually nanodots and nanoparticles),^[^ ^137^, ^138^ ^]^ and sacrificial agents (triethanolamine‐TEOA, 1,4‐dihydronicotinamide‐BNAH, triethylamine‐TEA, tributylamine‐Bu_3_N, ethylenediaminetetraacetic acid‐EDTA, and ascorbic acid etc.).^[^ ^8^, ^139^ ^]^ Compared to the complex situation mentioned above, light‐driven CO_2_RR coupled with the WOR (water oxidation reaction) process is a clean and promoting strategy for energy conversion and storation, and has become a hot but challenging research in recent years. In this way, electrons and holes migrate to the reduction and oxidation sites and react with adsorbed CO_2_ and H_2_O molecules. During the catalysis, H_2_O molecules can be oxidized to O_2_ or H_2_O_2_ and provide protons and electrons for reducing CO_2_ to the products (CO, HCOOH, CH_4_, C_2_H_4_, etc.) at the same time. This not only achieves the green conversion of CO_2_ and H_2_O to fossil fuels, but also avoids the drawbacks of the individual CO_2_RR and WOR half‐reaction systems, such as high cost, low atom economy, and potential environmental problems for additional photosensitizers and sacrificial agents. The simplest system for artificial photosynthesis consists of only light, water, CO_2_, and photocatalyst, where the efficient and stable photocatalyst is the core to realize the overall system. In this regard, wide‐bandgap semiconductor photocatalysts are required to ensure there is a large energy difference between electrons and holes and to match the potentials of CO_2_RR and WOR simultaneously. This implies that, under the premise of band alignment, the photocatalytic system not only needs to easily generate electron‐hole pairs under light excitation but also requires excellent charge separation behavior to satisfy the two half reactions. Given the recent surge of interest and the substantial potential in these integrated strategies, there is a growing need for a comprehensive review of this emerging field.

In this section, we have included the recent studies focused on developing sacrificial agent‐free photosystems aimed at practical applications. Furthermore, we have summarized the reported studies on CN, CTFs, and COFs materials in the newly updated tables (**Tables** **5**, **6**, **7**), which include the latest references and highlights the strategies, materials, and performance metrics of each system. The achievements can be represented by four aspects: 1) The majority of the reports have preferentially employed the solid‐gas mode for CO_2_ photoreduction. The solid‐gas system allows the catalysts to be uniformly dispersed on the substrate. In this system, water is dropped inside the reactor, evaporated to become H_2_O vapor by heating, or flowed into the reactor with CO_2_ gas. CO_2_ gas can flow into the reactor as a continuous gas or filled in a closed off‐line system as a saturated gas. The CO_2_ concentration can be adjustable with H_2_O acting as a sacrificial agent to provide protons and electrons which will be fed to CO_2_ for the generation of value‐added products. 2) To date, few single‐component polymeric photocatalysts have been reported to realize the overall paired half‐reactions of CO_2_ reduction and H_2_O oxidation. This type of polymeric materials requires that the combination of different building units within the framework should be perfectly matched, to ensure the obtained catalysts possess the appropriate band energy structure to achieve the overall process. This requires a more precise or demanding design, including the adjustment of the components, and the spatial orientation or connection patterns of the redox units. Therefore, effective strategies such as redox units, D‐A structures, D‐π‐A structures, π‐conjugated systems, and metallization engineers have been used to endow the organic photocatalysts with special qualities.^[^ ^123^, ^127^, ^140^, ^141^, ^142^ ^]^ 3) Benefiting from the strong interaction or synergistic effects between the different components, heterojunctions, formed by combining two or more materials, have achieved impressive results in the overall photocatalytic process. These two‐ or three‐component heterojunctions enable the effective interaction of two half‐reactions and finally realize overall paired half‐reactions. To date, designs such as Z‐scheme, Type II, S‐scheme, and molecular junction, which enhance photoexcited charge separation and fine‐tune electron delivery pathways, have become the main research directions.^[^ ^142^, ^143^, ^144^, ^145^, ^146^, ^147^, ^148^, ^149^ ^]^

**Table 5:** The comparison of sacrificial agent‐free PCO_2_RR photosystems over the reported CNs photocatalysts.

Catalysts	Strategy	System type	Light source	Esa^a)^/Ps^b)^/Ca^c)^	Product [µmol g^−1^ h^−1^]	Ref.
Fe_2_P/NVsCN	N.A.	Solid‐gas system R.T.	Xe lamp, 300W, > 400 nm	None	CO 22.48	[150]
Mn_1_Co_1_/CN	Casting sites	Solid‐gas system N.A.	Xe lamp, 300W, to cut off IR light	None	CO 47	[151]]
Ag‐CVCN	Suitable bandgap Casting sites	Solid‐gas system 10 °C	Xe lamp, 300W, AM 1.5G	None	CO 57.67	[152]
CN_X_/V_Bi_‐BOB	S‑scheme Casting dual sites	Solid‐liquid system 5 °C	Xe lamp, 300W	None	CO 16.89	[153]
NS‐P‐g‐C_3_N_4_‐Ni	Suitable bandgap Coplanarity	Solid‐liquid system N.A.	Xe lamp, 300W	None	HCOOH 18.1	[154]
CN‐CuPt	S‑scheme Casting sites	Solid‐liquid system 25 °C	Xe lamp, 300W, > 420 nm	None	C_2_H_4_ 778.6	[155]
C_6_N_7_	Suitable bandgap	Solid‐gas system 25 °C	Xe lamp, 300W, > 420 nm	None	CO 6.88 CH_4_ 17.21	[156]
C_4.5_N_5_	Suitable bandgap	Solid‐gas system 25 °C	Xe lamp, 300W, > 420 nm	None	CO 8.8 CH_4_ 13.5	[156]
C_3_N_3_	Suitable bandgap	Solid‐gas system 25 °C	Xe lamp, 300W, > 420 nm	None	CO 8.4 CH_4_ 13.1	[156]
PCCN‐10	Suitable bandgap N defect sites	Solid‐gas system 25 °C	Xe lamp, 300W,	None	CO 9.20 CH_4_ 15.07 C_2_H_6_ 99.14	[157]
PCCN‐10	Suitable bandgap Defect sites	Solid‐gas system N.A.	Sun light	None	C_2_H_6_ 43.17	[157]
CN@BiOI/CF	S‑scheme	Solid‐liquid‐gas system N.A.	Xe lamp, 300W, > 400 nm	None	CO 458.0	[158]
N_V_‐C_3_N_4_/β‐Bi_2_O_3_	Z‑scheme	Solid‐gas system 25 °C	Xe lamp, 300W, AM 1.5G	None	CO 30.56	[159]
N_V_‐C_3_N_4_	Suitable bandgap	Solid‐gas system 25 °C	Xe lamp, 300W, AM 1.5G	None	CO 11.43	[159]
g‐C_3_N_4_/β‐Bi_2_O_3_	Z‑scheme	Solid‐gas system 25 °C	Xe lamp, 300W, AM 1.5G	None	CO 16.74	[159]

^a)^ Esa (electron sacrificial agent);

^b)^ Ps (photosensitizer);

^c)^ Ca (cocatalyst);

R.T. (room temperature); N.A. (not applicable).

**Table 6:** The comparison of sacrificial agent‐free PCO_2_RR photosystems over the reported CTFs photocatalysts.

Catalysts	Strategy	System type	Light source	Esa^a)^/Ps^b)^/Ca^c)^	Product [µmol g^−1^ h^−1^]	Ref.
TiO_2_@CTF‐Py	Z‐scheme	Solid‐liquid system 25 °C	Xe lamp, 300W, > 320 nm	CoCl_2_	CO 43.34	[70]
TTCOF/NUZ	S‑scheme	Solid‐gas system R.T.	Xe lamp, 300W, > 420 nm	None	CO 6.56	[143]
CT‐COF	D‐A structure	Solid‐gas system 25 °C	Xe lamp, 300W, > 420 nm	None	CO 102.7	[160]
2D CN/COF‐TD	Type II junction	Solid‐liquid system 20 °C	Xe lamp, 300W,	None	CO 7.08 CH_4_ 2.37	[161]
CTF/Bi_19_S_27_Br_3_	Junction	Solid‐liquid system N.A.	Xe lamp, 300W, 320‐780 nm	None	CO 572.2 CH_4_ 0.38	[162]
Br‐COFs@BiOCl	Z‐scheme	Solid‐gas system 5 °C	Xe lamp, 300W, > 320 nm	None	CO 27.4	[163]
CTF‐IO	Suitable bandgap	Solid‐liquid system 25 °C	Xe lamp, 300W, > 420 nm	None	CO 118.69	[164]
CTF 2	Suitable bandgap	Solid‐gas system 30 °C	Xe lamp, 300W, Full spectrum	None	CO 25.85	[165]
SCTF	Suitable bandgap	Solid‐liquid system N.A.	Xe lamp, 300W, AM 1.5G	None	CO 6.18	[166]
AA‐CTF/TAPT	Z‐scheme	N.A. N.A.	Xe lamp, 300W,	None	CO 7.47	[167]
CTF‐AA/TiO2	Z‐scheme	Solid‐gas system Cooling water	Xe lamp, 300W, 320‐780 nm	None	CO 2.32 CH_4_ 9.19	[27]
CABB/CTF‐1	S‐scheme	Solid‐gas system N.A.	Xe lamp, 300W, > 420 nm	None	CO 30.73 CH_4_ 8.6	[168]
In‐MOF@TP‐TA	Type II junction	Solid‐gas system R.T.	Xe lamp, 300W, N.A.	None	CO 25.0 CH_4_ 11.67	[169]
CTF‐1	Suitable bandgap	Solid‐liquid system 25 °C	Xe lamp, 300W, > 400 nm	None	CO 13.0	[170]
CPB/CTF‐1	Type II junction	Solid‐liquid system 25 °C	Xe lamp, 300W, > 400 nm	None	CO 48.2	[170]
CPB/CTF‐1‐Ni	Type II Junction Casting sites	Solid‐liquid system 25 °C	Xe lamp, 300W, > 400 nm	Ni^2+^ cations	CO 86.5	[170]

^a)^ Esa (electron sacrificial agent);

^b)^ Ps (photosensitizer);

^c)^ Ca (cocatalyst);

R.T. (room temperature); N.A. (not applicable).

**Table 7:** The comparison of sacrificial agent‐free PCO_2_RR photosystems over the reported COFs photocatalysts.

Catalysts	Strategy	System type	Light source	Esa^a^/Ps^b^/Ca^c^	Product [µmol g^−1^ h^−1^]	Ref.
TTA‐Tz	N.A.	Solid‐liquid system N.A.	Xe lamp, 300W, > 420 nm	None	CO 82	[171]
TCOF‐MnMo_6_	N.A.	Solid‐gas system R.T.	Xe lamp, 300W, 400‐800 nm	None	CO 37.25	[172]
MCOF‐Ti_6_Cu_3_	Casting sites	Solid‐liquid system 25 °C	Xe lamp, 300W, AM 1.5G	None	HCOOH 169.8	[106]
Bi‐TTCOF‐Zn	Redox units	Solid‐liquid system 20 °C	Xe lamp, 300W, 420‐800 nm	None	CO 11.56	[107]
BTE‐TBD‐COF	π conjugation	Solid‐gas system N.A.	Xe lamp, 300W, 320‐780 nm	None	CO 382.03	[141]
TiO_2_‐INA@CuP‐Ph	Z‐scheme	Solid‐gas system R.T.	Xe lamp, 300W	None	CO 50.5	[146]
TpPa@IEF	N.A.	Solid‐gas system N.A.	LED lamp	None	HCOOH	[173]
Ga_2_O_3_/COF	Junction	Solid‐liquid system N.A.	Xe lamp, 300W, > 420 nm	None	CO	[174]
TpPa/ZIF‐8‐6G	Junction	Solid‐gas system 30 °C	Sun light	None	CO	[175]
HB‐TAPT+Co	D‐A structure	Solid‐liquid system N.A.	Xe lamp, 400 mW/cm^2^	None	CO	[176]
N_3_‐COFs	N.A.	Solid‐gas system 80 °C	500 W Xe lamp, 420‐800 nm	None	CH_3_OH	[177]
TTCOF‐Zn	N.A.	Solid‐ liquid system 25 °C	Xe lamp, 300W, 420‐800 nm	None	CO	[178]
CONs	N.A.	Solid‐gas system 25 °C	Xe lamp, 300W, 420‐750 nm	None	CO 132.2	[99]
COF‐318/TNF‐15	N.A.	Solid‐gas system N.A.	Xe lamp, 300W, > 420 nm	None	CO 70.1	[179]
TAPBB‐COF	Suitable bandgap	Solid‐gas system 80 °C	500 W Xe lamp, 200‐1000 nm	None	CO 24.6	[180]
Mo‐COF	Casting sites	Solid‐gas system constant temperature	Xe lamp, 300W, > 420 nm	None	C_2_H_4_	[181]
Pd_x_In_y_@N_3_‐COF	N.A.	Solid‐liquid system N.A.	Xe lamp, 300W, > 400 nm	None	alcohols	[182]
viCOF‐bpy‐Re	Casting sites Redox units	Solid‐gas? system Cons?e	Xe lamp, 300W, > 420 nm	None	CO 190.6	[183]
TFPT‐BDF‐COF	Suitable bandgap Low exciton binding	Solid‐gas system constant temperature	Xe lamp, 300W, > 420 nm	None	CO 158.1	[184]
COF C	D‐A‐D	Solid‐ liquid system R.T.?	Xe lamp, 300W, > 420 nm?	None	CO 25 CH_4_ 120	[185]

^a)^ E_sa_ (electron sacrificial agent);

^b)^ P_s_ (photosensitizer);

^c)^ C_a_ (cocatalyst); R.T. (room temperature); N.A. (not applicable).

**Figure 30:**
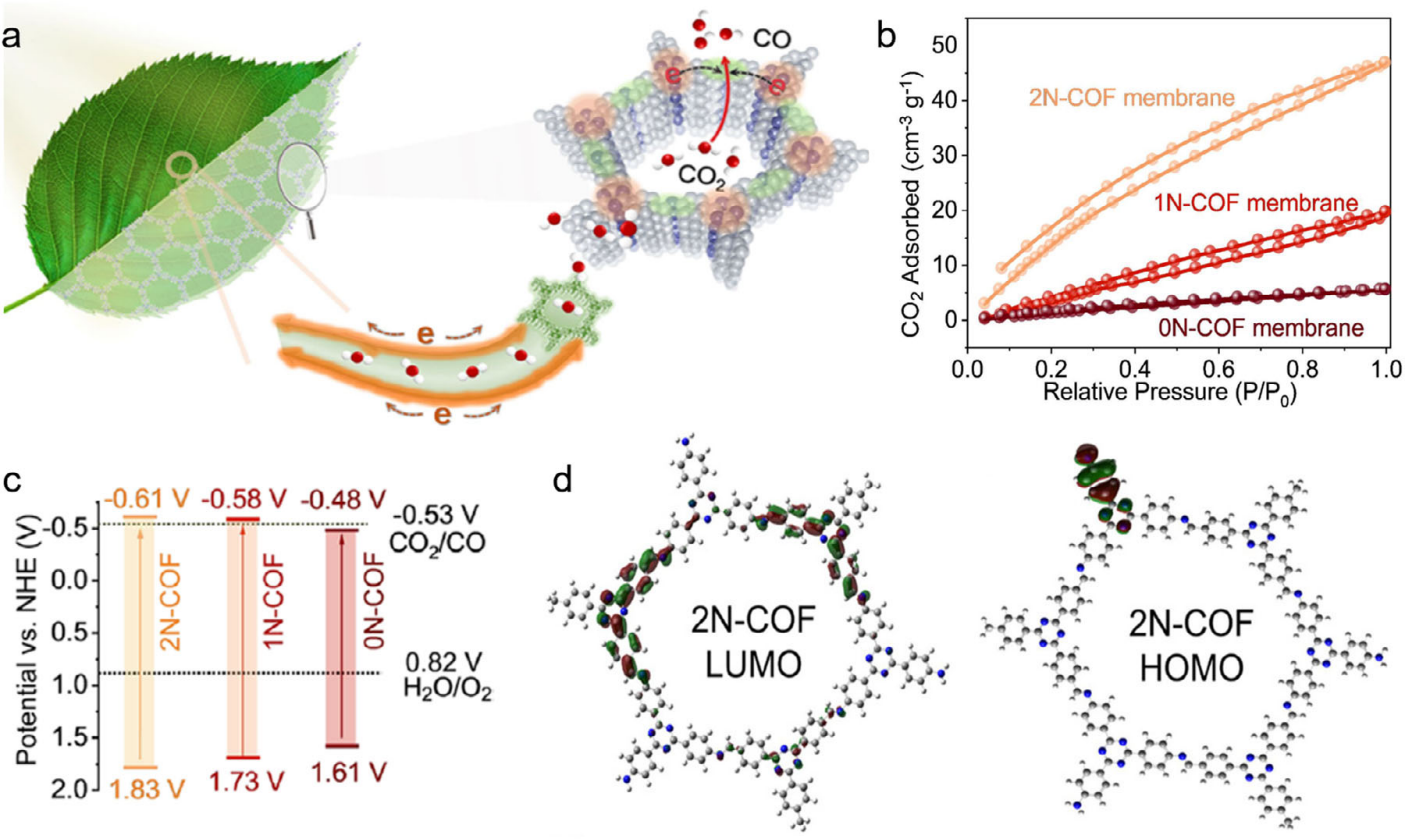
a) schematic illustration of 2N‐COF membrane‐based photocatalyst for PCO_2_RR in pure water. b) CO_2_ adsorption isotherms of the 2N‐COF membranes measured at room temperature. c) Optical band structure diagrams for facilitating PCO_2_RR‐WOR. d) The LUMO and HOMO levels of the catalyst.^[^ ^55^ ^]^ Copyright 2023 American Chemical Society.

For example, inspired by the architecture of natural leaves, Wang constructed a 2N‐COF‐based homogeneous COF membrane using triazine building blocks, achieving photocatalytic CO_2_ reduction in pure water (**Figure** **30**a).^[^ ^55^ ^]^ Resembling as an artificial leaf, CO_2_ is photoreduced to CO with the source of protons derived from gaseous water, while H_2_O have been photooxidized to O_2_. This membrane integrates stable light‐harvesting sites, efficient catalytic centers, and fast charge/mass transport channels within one 2N‐COF membrane‐based architectures. Porosity was assessed by nitrogen adsorption‐desorption, showing a BET surface area of 1031 m^2^ g^−1^. NLDFT analysis indicated a pore size of ∼2.1 nm and pore volume of 0.72 cm^3^ g^−1^. As shown in Figure 30b, the high porosity and surface area enable effective mass transfer, evidenced by strong CO_2_ uptake—a prerequisite for efficient catalysis. The optical bandgap, estimated via the Kubelka–Munk function, was 2.44 eV. The conduction band (CB) and the valence band (VB) lie at −0.61 and 1.83 V, respectively, which are sufficient for driving the CO_2_ reduction and H_2_O oxidation reactions in the meanwhile (Figure 30c). Under visible light, triazine units get photoexcited, transferring electrons through phenyl rings to imine sites. A record CO_2_ conversion rate of 310 µmol g^−1^ h^−1^ was achieved, with a peak apparent quantum efficiency (AQE) of 0.36% at 420 nm. DFT results show that the LUMO is distributed on the imine units and π‐system, suggesting imine bonds as electron acceptors. The HOMO, acting as the donor, localizes on triazine and phenyl rings. In the triazine–imine–triazine motif, triazine acts as an electron reservoir, donating electrons to imine units (Figure 30d). Additionally, we have introduced a conceptual roadmap outlining six key optimization principles for the design of efficient tandem CO_2_RR‐WOR photocatalysts: (1) maximizing light absorption via molecular conjugation, (2) precise band‐edge alignment, (3) creation internal charge‐separation field, (4) accumulation and control of reactants, (5) engineering of reactive sites, and (6) additional functionalization for performance tuning.

### 4.5 Promoting the Scalability of Applications Based on CO_2_ Conversion

**Figure 31:**
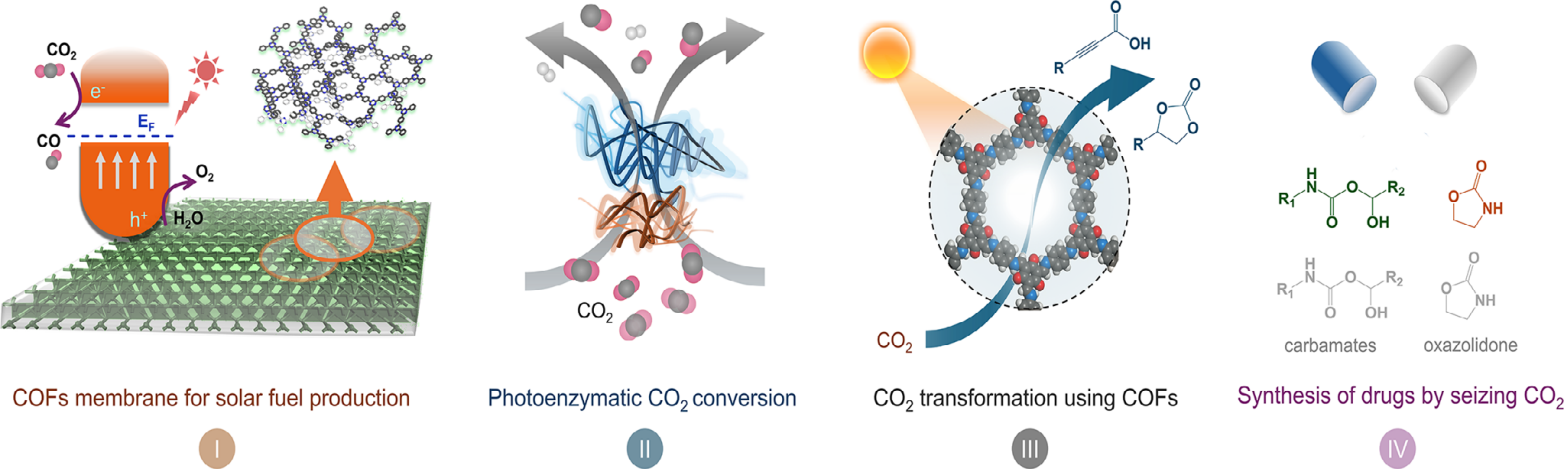
Schematic for enhancing scalable applications of CO_2_ conversion using polymeric photocatalysts.

Promoting scalable applications toward CO_2_ conversion via polymer‐based photocatalysts involves several innovative approaches in the future.^[^ ^122^, ^182^, ^186^, ^187^ ^]^ Recent research has highlighted polymeric materials as potential catalysts not only for solar fuel production but also for biochemical and pharmaceutical synthesis, presenting a promising pathway for sustainable chemical applications (**Figure** **31**). These avenues include: (I) Manufacturing film for solar fuel production: Baeg et al. fabricated an inexpensive and flexible film photocatalyst by using the 2D CTFs materials for the applicaiton of CO_2_ conversion.^[^ ^188^ ^]^ The photocatalytic activity was evidenced by the high formic acid formation rate, resulting from the photocatalytic properties of the COF‐based film that target selective solar fuel production from CO_2_. The material's efficiency is linked to its structural design, which supports photon absorption and charge transfer, both critical for scalable, sustainable solar‐driven CO_2_ reduction. (II) Photoenzymatic CO_2_ reduction: the photoenzymatic reduction of CO_2_ offers a valuable route for creating specialized biochemicals. For this process, Zhao et al. assembled photocatalyst‐enzyme systems for CO_2_ reduction using the COFs materials as the porous solid carrier to immobilize enzymes.^[^ ^54^, ^189^ ^]^ The engineered COFs with enzymatic functionalities promote CO_2_ reduction, combining catalytic stability with bio‐selectivity. Such assembled systems enable unique photochemical reactions suitable for scalable applications, particularly when considering the COFs’ modularity to be tailored to specific chemical transformations. The integration of enzymatic features in COFs for CO_2_ reduction expands the potential applications of these organic frameworks, positioning them as a bridge between synthetic and natural catalytic environments. (III) CO_2_ organic transformations in COFs: In recent years, COFs catalysts have shown exceptional versatility for catalyzing various CO_2_ organic transformations, supporting a range of applications in the chemical industry. CO_2_ fixation with epoxides to create carbonates has been well‐documented,^[^ ^189^, ^190^ ^]^ while the cyclization of alkenyl and alkynyl amines with CO_2_ to synthesize carbamates and oxazolidones has been discussed and studied.^[^ ^191^, ^192^ ^]^ For example, Islam et al. demonstrated that Pd(II)‐loaded COFs can catalyze the formation of oxazolidinone products from CO_2_ under sunlight. This work not only provides a new environmentally friendly route for oxazolidinone synthesis but also meets the demands for industrial‐scale CO_2_ capture and conversion using polymeric catalysts under mild conditions.^[^ ^193^ ^]^ At the same time, researchers have also explored CO_2_ conversion with alkynes and other substrates, demonstrating COFs’ role as the catalyst in the synthesis of diverse organic compounds.^[^ ^194^ ^]^ (IV) Drug synthesis using CO_2_ molecules: As noted, CO_2_ conversion has been employed in the synthesis of valuable drugs including carbamates and oxazolidones. For example, Velty et al. and Sengupta et al. have demonstrated catalytic encapsulation techniques using COFs to enable efficient CO2 fixation for drug synthesis under mild conditions.^[^ ^195^, ^196^ ^]^ Additionally, Zhao et al. and Khatun et al. described the role of polymeric catalysts in harnessing CO_2_ to synthesize small‐molecule drugs, emphasizing their tunability and potential for scaled‐up pharmaceutical applications.^[^ ^193^, ^197^ ^]^ The scalability of CO_2_ conversion for drug synthesis represents a significant advancement in “green chemistry” approaches within the pharmaceutical industry, reinforcing COFs as valuable materials for industrial drug production.

Moving forward, COFs and related photocatalytic frameworks are positioned to play an essential role in scalable CO_2_ conversion technologies. Their customizable structures, combined with high catalytic efficiency and the potential for integration with other catalytic systems, make them ideal candidates for broader applications in harnessing renewable energy, sustainable chemical manufacturing, and pharmaceuticals. Efforts to enhance the scalability of COFs by improvements in structural stability, efficiency under ambient conditions, and adaptability to industrial processes will be critical. By continuing to refine these materials’ catalytic performance and exploring their full potential across industries, COFs can become cornerstones in the global effort to reduce CO_2_ emissions and harness CO_2_ as a valuable resource for sustainable development.

### 4.6 Data‐Driven Insights for CO_2_ Capture Using Polymeric Photocatalysts

**Figure 32:**
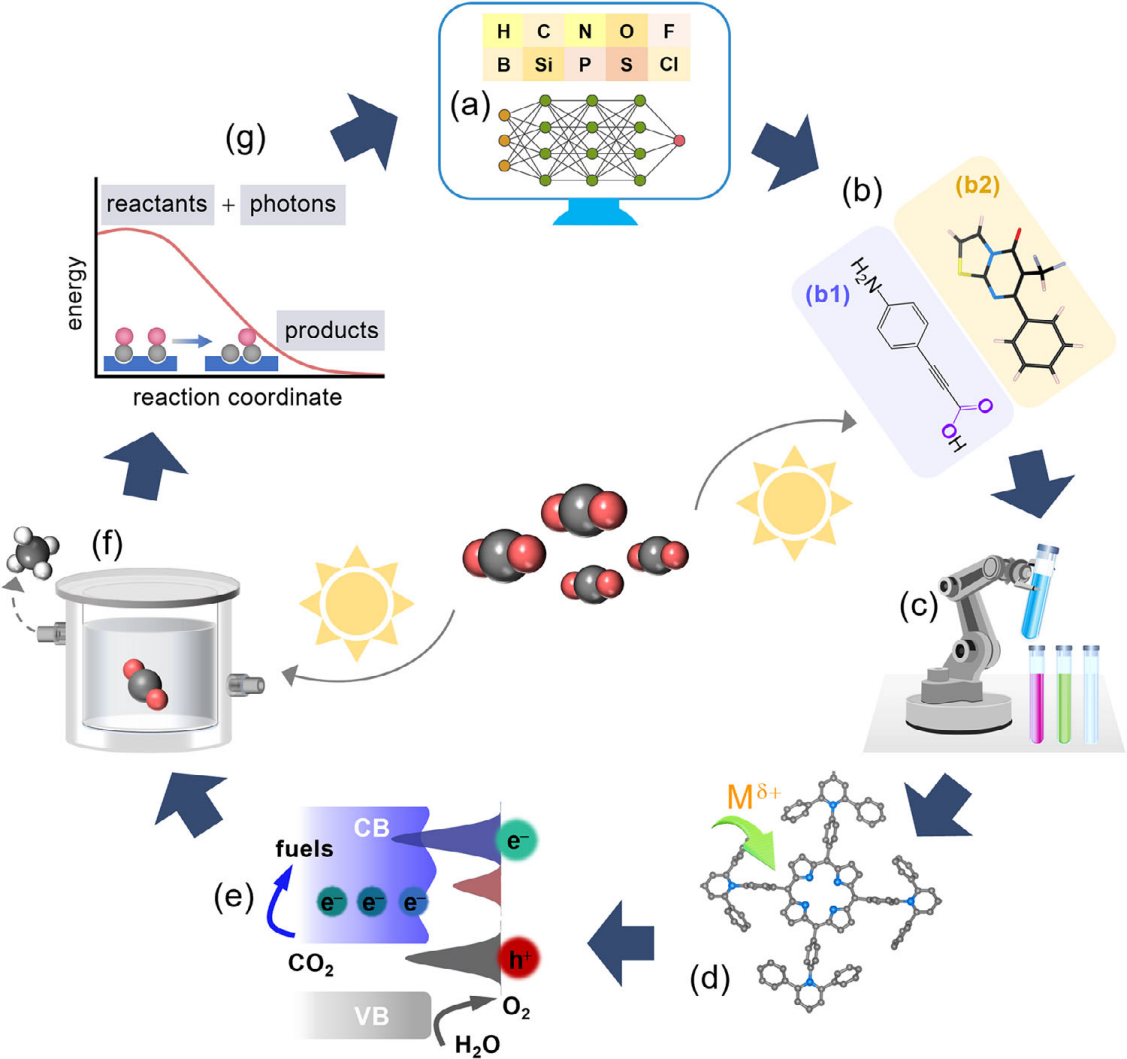
Data‐driven insights for designing organic photocatalysts for light‐driven CO_2_ fixation.

There are two main modes by which experiments and theory collaborate to advance scientific research. One approach is experiment‐led, where theoretical simulations are used to validate experimental results. The other is theory‐led, using computational models to guide material synthesis or material application. In recent years, we've entered a new era where human‐assisted material synthesis is increasingly integrated with theoretical design, synergistically advancing catalytic science.^[^ ^198^ ^]^ Polymeric materials have emerged as promising photocatalysts for CO_2_ conversion, offering vast synthetic possibilities but also challenges. At this juncture, by leveraging large language models to collect and analyze organic framework property datasets, we can accelerate the process to discover the photocatalysts toward PCO_2_RR. As shown in **Figure** **32**a–c, organic photocatalysts are typically composed of light elements (H, B, C, N, O, F, S, Cl, Br) connected via covalent bonds to form extended frameworks. To expedite the development of these materials, machine learning, and data‐driven models can be applied to screen organic monomers through high‐throughput computational predictions, assessing both their feasibility and synthesis conditions. This enables the rational pre‐design of organic photocatalysts to guide experimental synthesis and optimization. For example, Yaghi et al. recently developed an AI‐assisted platform that efficiently optimized COF‐323 synthesis. Through Bayesian optimization, the system identified optimal conditions within only 24 iterations, producing highly crystalline COF‐323.^[^ ^199^ ^]^ Following this strategy, data‐driven insights can integrate robotic experiments and high‐throughput computations to explore a wide structure‐property space across thousands of polymers.

Additionally, during the synthesis of COFs, CO_2_ itself can serve as a feedstock to produce organic monomers (See b1 part in Figure 32b). Several methods already exist to convert CO_2_ into small organic molecules, some of which are viable monomers for COF synthesis. In certain cases, these monomers are produced under light‐driven conditions.^[^ ^200^ ^]^ For instance, carboxylic acids generated via direct photocarboxylation of CO_2_ can serve as monomers to form ester‐ or amide‐linked COFs. Likewise, CO_2_ can react through amination or electrocatalytic processes to yield amines, another common COF monomer. Dicarboxylic acids obtained from carboxylation reactions can also be applied to synthesize amide‐ or ester‐linked COFs. Theoretical evaluations can validate these routes, and further optimization can be achieved using established organic condensation strategies. Robotic experimentation can facilitate large‐scale screening of reaction conditions, reducing both time and labor costs.^[^ ^201^ ^]^

Moreover, theoretical calculations, particularly DFT, are essential for evaluating the thermodynamic feasibility of materials and active sites in PCO_2_RR. This involves two key aspects: identifying highly active catalytic sites and analyzing the electronic structures of materials. The geometry and electronic structure of catalysts are critical in determining their performance, and optimization of these factors enhances efficiency and selectivity of CO_2_RR (Figure 32d). For example, in metallo‐covalent organic frameworks (M‐COFs), determining which metal‐site structure favors CO_2_ adsorption and activation is crucial. Zhang et al. designed dual‐site M‐COFs for efficient CO_2_ photoreduction to C_2_H_4_ based on theoretical insights.^[^ ^202^ ^]^ Additionally, DFT calculations of electronic band structures—such as band edges and band gaps—help assess the photocatalysts’ thermodynamic viability to drive the reactions. For instance, the HOMO‐LUMO transitions in LaNi‐Phen/COF‐5 were analyzed to elucidate La's role in enhancing CO_2_ conversion (Figure 32e).^[^ ^96^ ^]^ To fully understand the PCO_2_RR mechanism, it is essential to combine in situ experiments with theoretical simulations. Capturing reaction intermediates and identifying products helps infer reaction pathways (Figure 32f,g). Theoretical modeling of photophysical processes validates these pathways and enhances comprehension of the link between material structure and catalytic performance. Ultimately, combining experimental and theoretical insights enables data‐driven models to optimize the design of highly efficient catalytic materials for CO_2_ chemical fixation.

## 5 Conclusion

This review comprehensively discusses the fundamental aspects and development progress in PCO_2_RR by using the carbon‐nitrogen‐based polymeric photocatalysts. We outlined the mechanisms, historical milestones, and the urgent demand for efficient CO_2_ conversion technologies. Strategies for enhancing PCO_2_RR performance have been summarized, including advanced junction design, nonmetallic site engineering, crystallinity improvements, and interactions with metal nanoparticles, and metal complexes. We also addressed the challenges of optimizing structural topography and linkage designs to improve the CO_2_ conversion performance. Despite significant advancements, barriers to light absorption, energy transfer efficiency, product selectivity, and stability remain. Overcoming above issues requires continued innovation in design of polymeric materials and hybrid photocatalytic systems, paving the way for scalable and economically viable CO_2_ reduction technologies. Future work should emphasize reducing carbon contamination, promoting sacrificial agent‐free systems, developing scalable CO_2_ conversion technologies, and holding data‐driven chances. By bridging these gaps, polymeric materials‐based photocatalysis holds promise for a sustainable approach to CO_2_ fixation and energy conversion solutions. In conclusion, while substantial progress has been made in developing polymeric materials for CO_2_ reduction, achieving commercial viability and environmental sustainability will require ongoing efforts to overcome existing issues. By doing so, this review aims to unlock the full potential of photocatalysis as a transformative technology, inspiring more researchers to contribute to global efforts in mitigating climate change and advancing the transition to a low‐carbon economy.

## Conflict of Interest

The authors declare no conflict of interest.
